# Microglial IRF7-induced lipophagy impairment aggravates lipid droplet overload and impedes neurological recovery after ischemic stroke

**DOI:** 10.1186/s12974-026-03902-3

**Published:** 2026-06-17

**Authors:** Jing Yin, Yan Li, Yu-Lu Miao, Xue-Fang Song, Yao Cheng, Yu-Jie Zhai, Jia Ren, Cai-Hong Yang, Hui-Feng Zhang, Xiang-Nan Zhang, Li-Jun Yang, Yan-Ying Fan

**Affiliations:** 1 Department of Pharmacology, Shanxi Key Laboratory of Metabolic-Immune Homeostasis and Drug Innovation, Shanxi Medical University, Jinzhong, 030600 China; 2 Institute of Pharmacology & Toxicology, College of Pharmaceutical Sciences, Zhejiang University, Hangzhou, 310058 China; 3 Department of Pharmacology, Shanxi University of Medicine, Fenyang, 032200 China

**Keywords:** Stroke, Microglia, Lipophagy impairment, Lipid droplet, IRF7, STING

## Abstract

Lipid droplet (LD) accumulation in microglia results in a dysfunctional and proinflammatory state after ischemic stroke and worsens neurological outcomes; yet how this accumulation is regulated remains unclear. Interferon regulatory factor 7 (IRF7) is an immune regulatory factor whose role in lipid metabolism and autophagy has been increasingly studied in peripheral tissues. However, the role of IRF7 in microglial lipophagy (a selective autophagic process that targets LDs) and poststroke functional recovery remains unexplored. In this study, using a mouse photothrombotic ischemia (PTI) model, we observed that microglia in the peri-infarct region displayed persistent lipophagy impairment and LD accumulation for up to 21 days. Reanalysis of the single-cell RNA sequencing (scRNA-seq) dataset revealed that an *Irf7*^*high*^ microglial MG1 subcluster (disease-associated microglia) was significantly associated with autophagy and lipid metabolism poststroke. Furthermore, microglial *Irf7* conditional knockout (*Irf7* cKO) mice exhibited a significant rescue of lipophagy impairment and an alleviation of the ensuing LD accumulation in microglia, accompanied by enhanced synaptic plasticity and motor functional recovery during the subacute phase poststroke. Consistently, in the 15-month-old distal middle cerebral artery occlusion (dMCAO) model, *Irf7* cKO mice also displayed similar improvements. Similar results were also observed in vitro. Mechanistically, *Gnai2* was identified as a positively regulated transcriptional target of IRF7. In BV2 cells and primary microglia, *Gnai2* knockdown mitigated lipopolysaccharide (LPS)-induced lipophagy impairment, thereby reducing LD accumulation. This treatment also increased the level of phosphatidylcholine (PC), a key lipid for stabilizing small LDs as well as promoting autophagosome formation and autophagic flux. Consistently, microglial *Irf7* deletion or knockdown attenuated stroke- or LPS-induced PC reduction both in vivo and in vitro. Furthermore, exogenous supplementation with CDP-choline, an intermediate in PC synthesis, alleviated LD accumulation and lipophagy impairment, thereby improving motor function. Additionally, delayed administration of an inhibitor of stimulator of interferon genes (STING, an upstream target of IRF7) replicated the beneficial effects observed in *Irf7* cKO mice, and its effects were not further enhanced by microglial *Irf7* deletion. Taken together, these novel findings reveal that persistent impairment of microglial lipophagy is a key contributor to poststroke LD accumulation, and that IRF7 is involved in this process through direct transcriptional activation of *Gnai2*, which reduces the PC levels. Suppressing IRF7 with a STING inhibitor is a potential strategy for modulating microglial lipid metabolism and promoting functional recovery following stroke.

**Supplementary Information:**

The online version contains supplementary material available at 10.1186/s12974-026-03902-3.

## Background

Ischemic stroke, a cerebrovascular disorder caused by the obstruction of cerebral blood flow, is characterized by high mortality and disability rates [1, 2]. Even after successful recanalization during the acute phase, intense and persistent neuroinflammation extends into the subacute-to-chronic phases, where it hinders neural repair and ultimately results in poor functional recovery in most patients [3–6]. As the resident innate immune cells of the central nervous system (CNS), microglia act as both regulators and effectors of poststroke neuroinflammation [7]. Thus, microglial modulation is a promising therapeutic strategy to improve functional outcomes poststroke, underscoring the need to elucidate the underlying regulatory mechanisms.

In 2020, Marschallinger et al. identified a distinct subset of microglia in the aging brain characterized by excessive lipid droplet (LD) accumulation, termed lipid droplet-accumulating microglia (LDAM) [8]. These cells display a dysfunctional phenotype marked by heightened proinflammatory activity, impaired phagocytic capacity and excessive production of reactive oxygen species. Subsequent studies have reported the presence of LDAM in various CNS disorders, including Alzheimer’s disease, diabetes-associated cognitive impairment, demyelination and stroke [9–12]. LD, which are primarily composed of neutral lipids such as triglycerides and cholesterol, are specialized lipid storage organelles that participate in providing substrates for energy metabolism, metabolic stress responses and inflammatory mediator release [13, 14]. Recently, it was reported that LDAM exhibit a pronounced proinflammatory phenotype during the subacute phase poststroke, whereas pharmacological inhibition of LD formation using triacsin C (TrC, an acyl-CoA synthetase inhibitor) shifts oxygen-glucose deprived (OGD) or lipopolysaccharide (LPS)-stimulated microglia toward an anti-inflammatory state in vitro [12]. Thus, LD degradation is essential for restoring lipid metabolic homeostasis and preventing the amplification of neuroinflammation. Lipophagy, a selective autophagic process that targets LDs, is critical for LD degradation [15]. Emerging evidence suggests that impaired microglial lipophagy leads to excessive LD accumulation, thereby enhancing neuroinflammatory responses, aggravating neuronal injury and decreasing synaptic plasticity in CNS disorders [10, 16]. Consequently, modulating microglial lipophagy may represent a promising strategy for treating CNS disorders. However, the dynamic changes and regulatory mechanisms of microglial lipophagy following ischemic stroke remain poorly understood.

Interferon regulatory factor 7 (IRF7), a member of the interferon regulatory factor family, plays a pivotal role in multiple biological processes, including viral infection, innate immunity and inflammatory responses [17, 18]. Accumulating evidence indicates that IRF7 expression is upregulated in adipocytes and adipose tissues of obese humans and mice, whereas genetic deletion or suppression of IRF7 promotes thermogenesis and alleviates obesity [19–21]. More importantly, IRF7 is highly expressed in macrophages within atherosclerotic plaques in both humans and mice, and its downregulation mitigates macrophage-mediated inflammation and enhances cholesterol efflux, thereby contributing to disease regression [22]. In addition, IRF7 deficiency has been shown to promote autophagy, thereby alleviating inflammatory responses in pulmonary and kidney tissues [23, 24]. These findings suggest that IRF7 serves as a key regulatory factor linking inflammation to perturbations in lipid metabolism and autophagy. However, its specific role in the regulation of lipophagy remains unexplored.

Moreover, IRF7 is upregulated under various pathological conditions in the CNS, including aging [25], Alzheimer’s disease [26], stroke [27], subarachnoid hemorrhage [28] and traumatic brain injury [29, 30]. Meanwhile, elevated expression of IRF7 has been detected in microglia [28, 31–34], which facilitates their polarization toward an M1-like proinflammatory phenotype [28, 31]. However, its biological function in CNS disorders remains largely unclear, particularly the causal relationship between IRF7 and functional outcomes poststroke. Here, we hypothesize that microglial IRF7 aggravates LD accumulation and hinders functional recovery after ischemic stroke by exacerbating lipophagy impairment.

In this study, we report for the first time that ischemic stroke induces an impairment of microglial lipophagy that persists for up to 21 days in mice. Furthermore, we systematically investigated the role and underlying mechanisms of microglial IRF7 in regulating lipophagy poststroke. By integrating the single-cell RNA sequencing (scRNA-seq) datasets of ischemic brain tissue, the photothrombotic and distal middle cerebral artery occlusion stroke model, microglial *Irf7* conditional knockout (*Irf7* cKO) mice and in vitro gene silencing, we demonstrated that the suppression of microglial *Irf7* alleviated lipophagy impairment and LD accumulation, and subsequently enhanced synaptic plasticity and improved functional outcomes poststroke. Furthermore, *Gnai2* was identified as a direct downstream target of IRF7, which is involved in this process through the downregulation of phosphatidylcholine (PC). Additionally, pharmacological inhibition of stimulator of interferon genes (STING, an upstream target of IRF7) replicated the benefits observed upon microglial *Irf7* deletion. Taken together, these findings establish IRF7 as a promising target for modulating poststroke microglial lipid metabolism, and provide a novel therapeutic strategy for stroke rehabilitation.

## Materials and methods

### Animals

Male adult mice (aged 8–11 weeks, body weight 20–23 g) and 15-month-old mice, both wild-type (WT) and genetically modified strains, were used in this study. C57BL/6 mice were obtained from the Laboratory Animal Center of Shanxi Medical University. *Irf7*^*fl/fl*^ mice and *Cx3cr1-Cre* mice, both maintained on a C57BL/6 background, were obtained from Cyagen Biosciences, Inc. All the animals were maintained in a specific pathogen-free facility at the Laboratory Animal Center of Shanxi Medical University under strictly controlled environmental conditions (12 h light/dark cycle, 22 ± 2 °C), with unrestricted access to food and water throughout the experimental period. To generate microglial *Irf7* conditional knockout mice, homozygous *Irf7*^*fl/fl*^ mice were crossed with heterozygous *Cx3cr1-Cre* mice, yielding *Cx3cr1-Cre; Irf7*^*fl/fl*^ mice (referred to as “*Irf7* cKO”) and their littermate controls (referred to as “*Irf7*^*fl/fl*^”). The breeding strategy and genotyping results are shown in Fig. S1, and the sequences of primers used for genotyping are provided in Table S1. All experimental procedures strictly complied with the National Institutes of Health (NIH) Guide for the Care and Use of Laboratory Animals and the ARRIVE 2.0 guidelines and were approved by the Institutional Animal Care and Use Committee of Shanxi Medical University (Approval No. SYDL2025040).

### Photothrombotic ischemia (PTI) model

As previously described [35], the mice were anesthetized with isoflurane and placed in a stereotaxic frame. The skull was exposed, and Rose Bengal solution (10 mg/mL dissolved in 0.9% saline, 100 mg/kg, i.p.; Cat# 330000, Sigma, USA) was administered. After 5 min, the skull was illuminated with a 2 mm-diameter cold light source (26,000 lx; OPLENIC, China) for 20 min to induce photothrombosis in the right hemisphere. The illumination site was positioned 1.5 mm lateral to the bregma. Sham-operated animals underwent the same surgical procedure but were administered only the vehicle solution. The mice were kept at 37 ± 0.5 °C until they fully recovered consciousness and spontaneous movement.

### Distal middle cerebral artery occlusion (dMCAO) model

As previously described [36], male 15-month-old mice were used to establish the dMCAO model. Mice were anesthetized with isoflurane, and a midline cervical incision was made to expose and ligate the left common carotid artery. Subsequently, a second skin incision was made between the left eye and left ear. Under a stereomicroscope, the temporalis muscle was bluntly separated, and the skull was carefully thinned using a microdrill to expose the distal portion of the middle cerebral artery (MCA). The exposed MCA was then permanently occluded using bipolar electrocoagulation forceps. Regional cerebral blood flow was monitored using a laser Doppler flowmeter (RFLSI Ⅲ, RWD, China). Sham-operated mice underwent the same surgical procedures without MCA coagulation. Finally, all incisions were sutured and disinfected, and body temperature was kept at 37 ± 0.5 °C until the mice recovered consciousness and spontaneous activity.

### Drug administration

The acyl-CoA synthetase inhibitor triacsin C (TrC; Cat# HY-N6707; MedChemExpress, USA) was intranasally administered to mice at a dose of 10 µM in 10 µL 0.9% saline, beginning at day 3 after PTI and then every other day until day 14. The CDP-Choline (Cat# HY-B0739; MedChemExpress, USA) was intraperitoneally administered to mice at a dose of 100 mg/kg, beginning at day 3 after PTI, and then every other day until day 14 [37]. The STING inhibitor C-176 (Cat# HY-112906; MedChemExpress, USA) was intraperitoneally administered to mice at a dose of 12.2 mg/kg, beginning at days 1, 3, or 7 after PTI, and then every other day until day 14 [38]. An equivalent volume of vehicle was administered to sham groups according to the same treatment schedule.

### Gait test

As previously described [39], video recordings of mouse gait were acquired using the DigiGait Imaging (Mouse Specifics, Inc., USA). Before stroke induction, the mice underwent motorized treadmill training for two consecutive days. During testing, each mouse was placed on a transparent treadmill running at 12 cm/s, with a high-speed digital camera positioned underneath to capture paw placement. For each mouse, gait data were collected from three independent trials. During each trial, 5 s of continuous locomotion was recorded. At day 3 after injury, mice were assigned to experimental groups by a blinded investigator according to the severity of behavioral deficits. Animals with excessively mild or severe deficits were excluded from further analysis. All videos were analyzed by investigators who were blinded to the group allocation using DigiGait Analysis software (Mouse Specifics, Inc., USA) to quantify the left forelimb (LF) and left hindlimb (LH) gait parameters, which included stride frequency, stride length, stride width, paw area and ataxia coefficient.

### Neurological deficit score

As previously described [40], neurological deficit scoring was performed to assess motor impairment in mice. Briefly, mice were placed on a flat surface, and the tail was gently lifted until the forepaws were off the surface; the degree of flexion and adduction of the forelimbs and hindlimbs was scored (0–2). Mice were then suspended by the tail, and the extent of C-shaped body bending toward one side was evaluated (0–2). Next, mice were allowed to grasp a 2-mm diameter steel wire with their forepaws to assess grip strength (0–3). Finally, mice were placed on a flat surface, and the hindlimbs were displaced laterally by 1–2 cm to evaluate the placing/reflex response (0–3). All assessments were performed and data were collected by investigators blinded to the experimental groups.

### Infarct volume measurement

Mouse brains were sectioned into 20 μm coronal slices using a cryostat (CM1950; Leica, Germany). For the PTI model, serial sections were collected from + 1.18 mm to − 1.22 mm relative to bregma at 0.48 mm intervals. For the dMCAO model, serial sections were collected from + 1.18 mm to − 2.66 mm relative to bregma at 0.48 mm intervals. Sections were stained with 1% toluidine blue, and images were acquired using a light microscope (DM2500; Leica, Germany). Infarct areas were quantified using ImageJ FIJI. Infarct volume was calculated according to the following formula: infarct volume = Σ infarct area of each slice × 0.48 mm.

### Real‑time quantitative PCR (RT‒qPCR)

Total RNA was extracted with an RNA extraction kit (Cat# B5113121; Sangon, China), and the RNA concentration was quantified using a microplate reader. The isolated RNA was reverse-transcribed using a reverse transcription kit (Cat# RK20433; ABclonal, China), after which RT‒qPCR analysis was performed with SYBR Green Master Mix (Cat# 11184ES08; Yeasen, China) on a CFX96™ Real-Time System (Bio-Rad, USA). The primer sequences are provided in Table S2.

### Western blot

Total protein was extracted with RIPA lysis buffer (Cat# AR0102; Boster, China) supplemented with protease inhibitor (PMSF; Cat# AR1178; Boster, China) and phosphatase inhibitor (Cat# AR1183; Boster, China). Equal amounts of protein were separated, transferred, blocked, and incubated with primary antibodies overnight at 4 °C. On the following day, the membranes were washed three times and incubated with secondary antibody for 2 h. Protein bands were detected using enhanced chemiluminescence (Cat# MA0186; MeilunBio, China) reagents on a Bio-Rad ChemiDoc imaging system, and were quantified using Image Lab software. All antibodies are provided in Table S3.

### Immunofluorescence staining

For tissue immunofluorescence, mouse brains were cut into 20 μm sections using a cryostat (CM1950; Leica, Germany). After antigen retrieval, permeabilization and blocking, the brain sections were incubated with primary antibodies overnight at 4 °C. On the following day, the brain sections were incubated with secondary antibodies for 2 h. Finally, the sections were stained with DAPI solution (Cat# AR1176; Boster, China) for 10 min and mounted using an anti-fluorescence quencher (Cat# AR1109; Boster, China). Whole-brain images of Iba-1 and GFAP staining were acquired using the TissueFAXS Spectra imaging system (TissueGnostics, Austria). Fluorescence images were captured using a confocal microscope (FV3000; Olympus, Japan). Four to six mice were included in each experimental group. For each mouse brain sample, the mean value from four random fields within the peri-infarct region was used for statistical analysis.

For cell immunofluorescence, the cells were permeabilized and blocked, followed by incubation with primary antibodies overnight at 4 °C. The following day, the cells were incubated with secondary antibody for 2 h. Finally, BODIPY and DAPI were used to stain the LDs and nuclei. Fluorescence images were captured using a confocal microscope. Three to six coverslips were included in each experimental group. For each coverslip, the mean value from five random fields was used for statistical analysis. All antibodies are provided in Table S4.

### Golgi-Cox staining

Golgi-Cox staining was conducted by using the FD Rapid GolgiStain™ Kit (Cat# PK401, FD Neuro Technologies, USA). Briefly, harvested brains were immersed in a solution A/B mixture for 14 days and subsequently transferred to solution C for 3 days. The brains were coronally sectioned at a thickness of 80 μm and stained. Golgi-stained neurons were imaged from the peri-infarct cortex. High-resolution images were captured using a confocal microscope and imported into ImageJ FIJI for quantitative analysis. In detail, neuronal complexity was evaluated using the Simple Neurite Tracer (SNT) plugin in ImageJ FIJI. Individual neurons were manually reconstructed by tracing dendritic processes from their origin at the soma to their terminal endpoints to obtain the total dendritic length. Sholl analysis was subsequently performed by placing concentric circles centered on the soma, starting at an initial radius of 3 μm with a fixed radial interval of 8 μm, and the number of dendritic intersections at each radius was quantified. For dendritic spine analysis, secondary or tertiary dendritic branches were selected, and spine density was determined by manually counting the number of spines along dendritic segments of defined length. Spine density was expressed as the number of spines per 10 μm of dendrite. In addition, spines were classified into different morphological subtypes, and subtype-specific spine densities were further quantified. All analyses were performed by investigators blinded to the experimental groups. Six mice were used in each experimental group. For each mouse brain sample, six neurons or six dendrites (from four fields of view) were analyzed from the peri-infarct cortical region, and the average values were included for statistical analysis.

### Confocal imaging and 3D reconstruction

Confocal images were acquired using a laser-scanning confocal microscope (FV3000; Olympus, Japan), and the samples were scanned using ×40 or ×100 oil immersion objectives. Z-stack images were collected at 2-µm intervals throughout the entire thickness of the sample. For 3D reconstruction, the Z-stack datasets were processed using Imaris 9.9 software (Bitplane, Switzerland). Thresholding and surface rendering were applied uniformly across groups. Quantitative morphometric analyses, including PLIN2 volume, p62 volume, Iba-1⁺ volume and GFAP⁺ volume were conducted on the basis of the reconstructed 3D images.

### Transmission electron microscope (TEM)

Mouse brains and cell samples were postfixed with 1% osmium tetroxide, dehydrated in acetone, and embedded in Epon-812 resin. Ultrathin Sects.  (60–90 nm) were prepared, stained with uranyl acetate and lead citrate, and imaged using a transmission electron microscope (JEM-1400FLASH, JEOL, Japan). Three samples were used in each experimental group. For each sample, three random fields were captured, and a total of nine fields were used for statistical analysis.

### Primary microglia culture

Primary microglia were isolated from the cerebral cortices of neonatal mice (within 24 h post-natal). After the cerebellum, meninges, and blood vessels were removed, the remaining cortical tissue was digested with prewarmed 0.25% trypsin (without EDTA) for 15 min. The dissociated mixed glial cells were cultured in MEM supplemented with 10% fetal bovine serum and 1% penicillin/streptomycin for 7–10 days. To obtain highly purified microglia, the culture flasks were shaken to detach the microglia from the adherent glial layer. Immunofluorescence staining confirmed that the purity of the cultured microglia exceeded 90%.

### BV2 cell line culture

BV2 (Cat# CL-0493; Procell, China), a murine microglial cell line, was cultured in DMEM supplemented with 10% fetal bovine serum and 1% penicillin/streptomycin. The cells were enzymatically dissociated using 0.25% trypsin-EDTA at approximately 90% confluence and passaged for subsequent experiments.

### Cell treatment

Cells were exposed to varying concentrations of lipopolysaccharide (Cat# L2630; Sigma, USA) for varying durations, and 1000 ng/mL for 24 h was selected for use in subsequent experiments. Rapamycin (10 nM; Cat# HY-10219; MedChemExpress, USA) was administered for 24 h [41], and the STING inhibitor C-176 (1 µM; Cat# HY-112906; MedChemExpress, USA) was added 30 min before LPS stimulation [38].

### siRNA transfection

Cells were transfected for 48 h prior to treatment using the riboFECT™ CP Transfection Kit (Cat#C10511; RiboBio, China). BV2 cells and primary microglia were transfected with 50 nM *Irf7* siRNA, 50 nM *Gnai2* siRNA, or the corresponding negative control (NC) siRNA. The siRNA target sequences are detailed in Table S5.

### mCherry-EGFP-PLIN2 and mCherry-EGFP-LC3 adenovirus transfection

BV2 cells were transfected for 24 h with a tandem fluorescent adenovirus at a multiplicity of infection of 100 [41]. For the mCherry-EGFP-PLIN2 adenovirus, yellow fluorescence (mCherry and EGFP colocalization) indicates that PLIN2 is in a neutral environment and has not entered the autolysosome; red fluorescence (mCherry only) indicates that the EGFP signal has been quenched by the acidic environment, suggesting that the LD has fused with the lysosome and entered the autophagic degradation stage. For the mCherry-EGFP-LC3 adenovirus (Cat# HBAD-1006; HANBIO, China), yellow fluorescence (mCherry and EGFP colocalization) represents autophagosomes, whereas red fluorescence (mCherry only) represents autolysosomes, because EGFP is quenched in the acidic environment. The data from 30 cells per group from three independent experiments were quantified using ImageJ FIJI software.

### BODIPY staining

BODIPY, a fluorescent dye, is typically used to detect LDs. The cells were incubated with BODIPY 493/503 (1:1,000 dilution from a 1 mg/mL stock solution in DMSO; Cat# D3922; Thermo Fisher Scientific, USA) at 37 °C for 30 min. ImageJ FIJI software was used to analyze the number and size of LDs and the percentage of BODIPY^+^ cells.

### Phosphatidylcholine (PC) determination

The PC levels were quantified using a Phosphatidylcholine Colorimetric Assay Kit (Cat# E-BC-K796-M; Elabscience, China). Briefly, brain tissue or cell samples were homogenized in extraction buffer, and the supernatants were collected after centrifugation. Then the samples were incubated with the working reagent, and the optical density was detected at 550 nm. PC concentrations were calculated on the basis of a standard curve.

### Chromatin immunoprecipitation (ChIP) assay

Chromatin immunoprecipitation was performed using the SimpleChIP^®^ Enzymatic Chromatin IP Kit (Cat# 9003; Cell Signaling Technology, USA). In brief, BV2 cells were crosslinked in 1% formaldehyde for 10 min. The nuclei were subsequently isolated and digested with micrococcal nuclease at 37 °C for 20 min to obtain 150–900 bp chromatin fragments. After enzymatic digestion, the chromatin was further sheared by brief sonication to ensure complete fragmentation. The sheared chromatin was immunoprecipitated overnight at 4 °C with an anti-IRF7 antibody (1:20; Cat# ABF130; Sigma‒Aldrich, USA) or normal rabbit IgG as a negative control, along with the protein G magnetic beads provided in the kit. The protein‒DNA complexes were eluted, the cross-links were reversed, and the purified DNA was analyzed by RT‒qPCR. The results were normalized to 2% input DNA and are presented as percent input. The primer sequences targeting the promoter region of *Gnai2* are detailed in Table S6.

### Single-cell RNA sequencing dataset reanalysis

Publicly available scRNA-seq data (GSE261494, GSE225948) [42, 43] were obtained from the Gene Expression Omnibus. For the GSE261494 dataset, samples included two sham mice and four mice at day 14 after permanent distal middle cerebral artery occlusion (pdMCAO). For the GSE225948 dataset, samples included four sham mice and four mice at day 14 after transient middle cerebral artery occlusion/reperfusion (tMCAO/R). Both datasets were processed using the same analytical workflow. Raw count matrices were processed and analyzed using Scanpy (v1.11.4) in Python 3.11 with default parameters unless otherwise specified.

For each sample, an individual AnnData object was constructed and subjected to quality control (QC) using *sc.pp.calculate_qc_metrics*. Cells deviating by more than five median absolute deviations from the sample-specific median for any metric were removed. After QC, all the samples were merged, data were then normalized on a per-cell basis using *sc.pp.normalize_total* and log-transformed with *sc.pp.log1p* for downstream dimensionality reduction and clustering analyses.

Highly variable genes (HVGs) were identified from the quality-controlled, normalized data and used for principal component analysis (PCA), clustering, and visualization. PCA was performed on the HVGs, retaining the top 50 principal components. Batch-corrected latent representations were generated using an SCVI model (scvi-tools v1.2.1), followed by the construction of a k-nearest neighbor graph. Uniform manifold approximation and projection (UMAP) was applied for dimensionality reduction and visualization, and clustering was performed using the Leiden algorithm (resolution = 0.6). Cell types were annotated using scType and manually refined on the basis of canonical marker genes. Microglia were then subsetted and reanalyzed using the same workflow, with Leiden clustering at a resolution of 0.3.

To examine *Irf7*-associated transcriptional programs, MG1 microglia were stratified into *Irf7*^*high*^ and *Irf7*^*low*^ groups on the basis of mean *Irf7* expression. Differential expression analysis within MG1 was performed using *sc.tl.rank_gene_groups*, with genes meeting log2FC > 1 and adjusted *p* < 0.05 defined as differentially expressed genes (DEGs). Gene ontology (GO) enrichment of the DEGs was performed with *clusterProfiler* (v4.10.0). To account for dropout effects, MAGIC (v3.0.0) was applied to the log-normalized microglial expression matrix prior to correlation analysis. Spearman correlation was then used to assess gene*–Irf7* associations, and genes subjected to Kyoto Encyclopedia of Genes and Genomes (KEGG) pathway enrichment (*q* < 0.05). The relationship between *Irf7* and *Gnai2* expression was visualized using *ggstatsplot* (v0.12.2) with linear regression and 95% confidence intervals.

### Statistical analysis

All the data were collected and analyzed in a blinded manner. Statistical analyses were performed using GraphPad Prism version 9.5.1. Prior to analysis, the data were subjected to the Shapiro‒Wilk test for normality and Levene’s test for homogeneity of variance. Unpaired t test was used for comparisons between two groups. One-way ANOVA was used for experiments with a single independent variable, and two-way ANOVA was applied for those with two independent variables, followed by Tukey’s or Bonferroni’s post hoc correction for multiple comparisons. The data are presented as the mean ± SEM. *P* < 0.05 was considered to indicate statistical significance.

## Results

### Sustained microglial lipophagy impairment following ischemic stroke

**Fig. 1:**
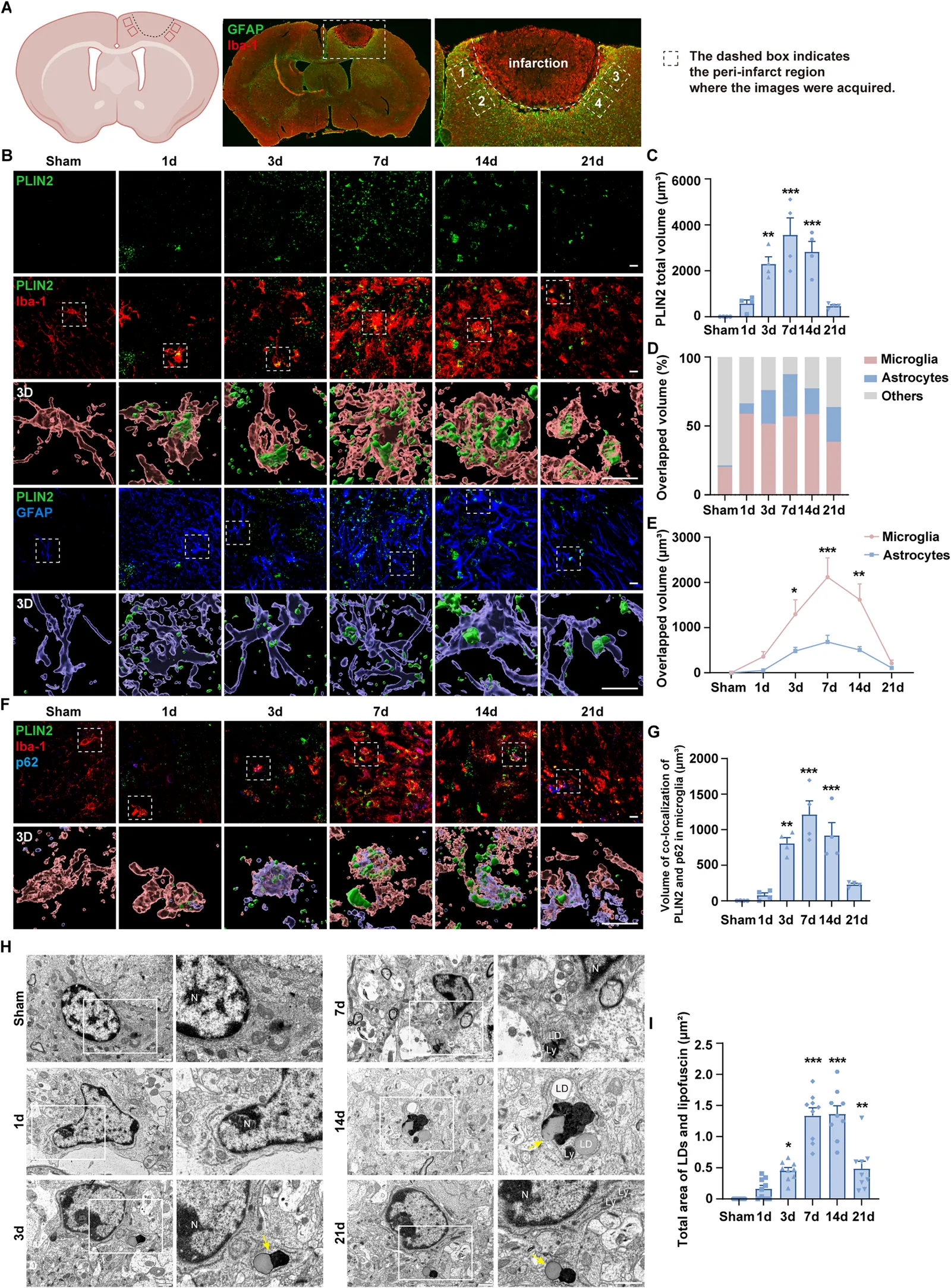
Sustained microglial lipophagy impairment following ischemic stroke. **A** Schematic illustration of the imaging location in the peri-infarct region after PTI. **B** Representative immunofluorescence images showing colocalization of PLIN2 (LDs) with Iba-1 (microglia) and GFAP (astrocytes) in the peri-infarct region from sham and PTI mice at 1, 3, 7, 14 and 21 days after PTI, along with magnified 3D-reconstructed images. Scale bars: 10 μm. **C**-**E** Quantification of PLIN2 total volume, the percentage of its overlap volume, and its volume separately in microglia and astrocytes. *n* = 4 mice per group. **F** Representative immunofluorescence images showing colocalization of PLIN2 and p62, along with magnified 3D-reconstructed images. Scale bars: 10 μm. **G** Quantification of the colocalization volume of PLIN2 and p62 in microglia. *n* = 4 mice per group. **H** Representative TEM images captured at both low and high magnification. Lipofuscin (yellow arrow), lipid droplet (LD) and lysosome (Ly). Scale bars: 1 μm. **I** Quantification of the total area of LDs and lipofuscin. *n* = 9 cells from 3 mice per group. ^*^P < 0.05, ^**^P < 0.01, ^***^P < 0.001 vs. sham group in (**C**), (**G**) and (**I**), one-way ANOVA with Tukey’s post hoc test. ^*^P < 0.05, ^**^P < 0.01, ^***^P < 0.001 vs. astrocytes in (**E**), two-way ANOVA with Bonferroni’s post hoc test. Data are presented as mean ± SEM

To determine whether microglial lipophagy impairment persists in the mouse brain poststroke, we systematically evaluated LD accumulation and lipophagy in the peri-infarct region at 1, 3, 7, 14 and 21 days post-PTI. The schematic diagram of the peri-infarct area is shown in Fig. 1A. First, we assessed the LD distribution by costaining brain sections for perilipin 2 (PLIN2, a major LD protein), the microglial marker Iba-1 and the astrocyte marker GFAP. The results showed that the PLIN2 total volume progressively increased as early as day 1 poststroke, peaked between days 3 and 14, and subsequently decreased (Fig. 1B, C). Moreover, PLIN2 was predominantly distributed in Iba-1⁺ microglia and GFAP⁺ astrocytes, and its expression was markedly higher in Iba-1⁺ microglia than in GFAP⁺ astrocytes at days 3–14 after PTI (Fig. 1B, D, E). To address the potential contribution of infiltrating macrophages, we performed double immunofluorescence staining for TMEM119 (a microglia-specific marker) and Iba-1 to examine the spatiotemporal distribution of microglia and macrophages in the peri-infarct region after PTI. The results showed that macrophages (TMEM119^−^Iba-1^+^) accounted for approximately 50–60% of Iba-1^+^ cells at day 1 after PTI, whereas from days 3 to 14, the majority of Iba-1^+^ cells were microglia (TMEM119^+^Iba-1^+^), accounting for more than 90% (Fig. S2). Therefore, microglia represented the predominant Iba-1^+^ population in the peri-infarct region during the subacute phase, indicating minimal macrophage involvement.

To provide direct evidence linking LDs to functional recovery, TrC (an acyl-CoA synthetase inhibitor) was intranasally administered every other day from days 3 to 14 postinjury to reduce LD formation (Fig. S3A). This treatment significantly reduced the PLIN2 total volume, the number and volume of Iba-1⁺ microglia (Fig. S3B–F), and improved the motor performance of the left forelimb and left hindlimb at days 7–14 after PTI, as evidenced by a decreased stride frequency and ataxia coefficient and increased stride length, stride width and paw area (Fig. S3G, H), suggesting that reducing LD formation is beneficial for functional recovery. Next, we assessed autophagic activity using the autophagy initiation marker beclin 1, the autophagy marker LC3 and the autophagy substrate p62. The results showed that beclin 1 expression increased only modestly, whereas the LC3Ⅱ/Ⅰ ratio and LC3Ⅱ significantly increased between days 3 and 14, and p62 expression markedly increased from day 7 to day 21 after stroke (Fig. S4), indicating a long-term blockade of autophagic flux instead of increased autophagic activity. Then, we performed co-immunostaining of PLIN2 and p62 to assess lipophagy in microglia after PTI. The results showed that the colocalization volume of PLIN2 and p62 in microglia was significantly increased at days 3–14 after PTI (Fig. 1F, G). More importantly, TEM showed that the total area of free LDs and lipofuscin within microglia was significantly increased from days 3 to 21 poststroke (Fig. 1H, I). These results suggest that the accumulation of LDs and impaired lipophagy persist in microglia following ischemic stroke.

### *Irf7*^*high*^ microglia subcluster engages autophagy and lipid metabolism pathways following ischemic stroke

**Fig. 2:**
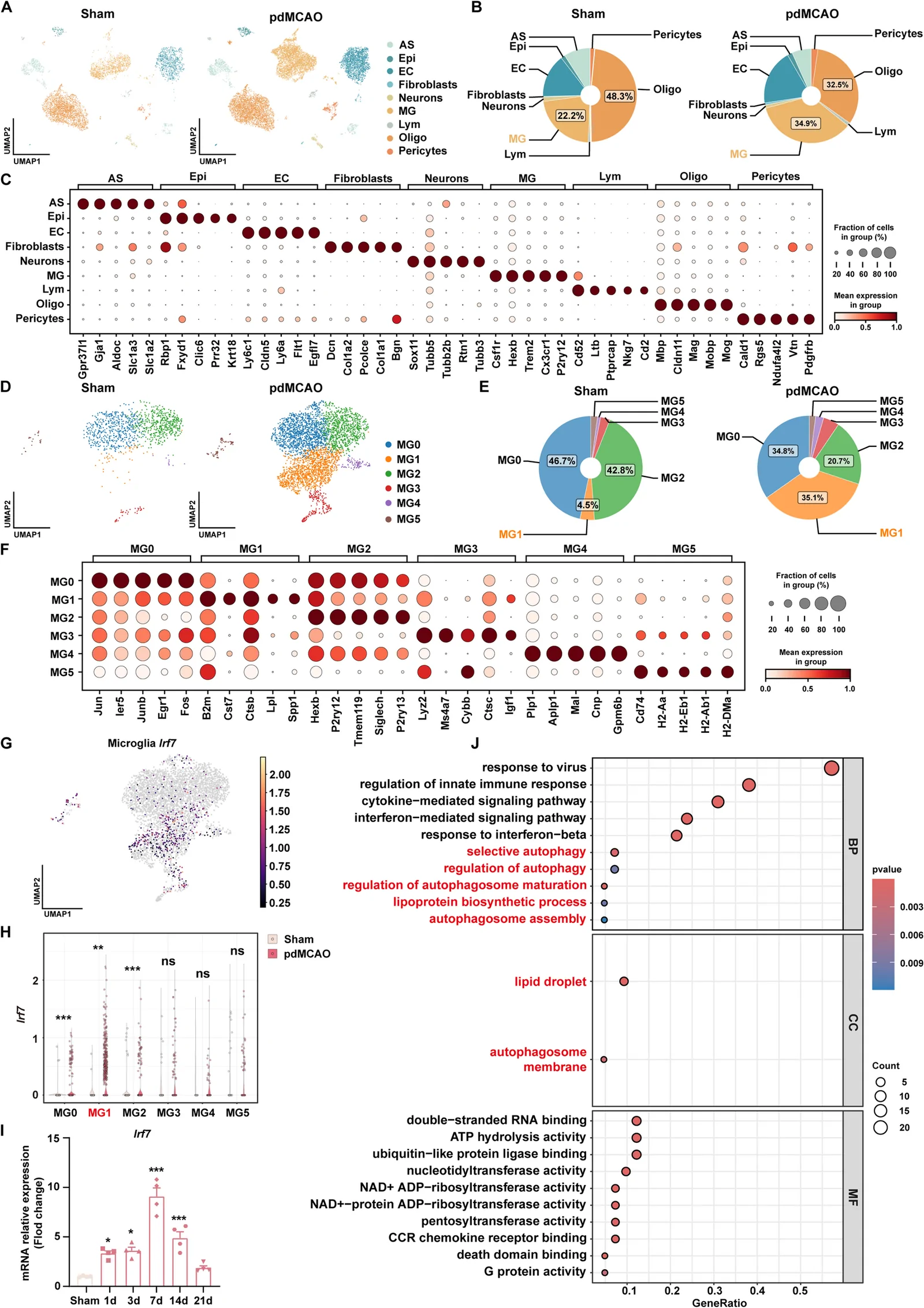
*Irf7*^*high*^ microglia subcluster engages autophagy and lipid metabolism pathways following ischemic stroke. **A** UMAP visualization of major cell types identified by scRNA-seq from sham and pdMCAO groups. **B** Pie plots showing the percentage of each cluster. **C** Dot plot showing the expression of marker genes across cell types shown in (**A**). **D** UMAP visualization of microglia subclusters from sham and pdMCAO groups. **E** Pie plots showing the percentage of each microglia subcluster. **F** Dot plot showing the expression of marker genes across microglia subclusters in (**D**). **G** UMAP visualization illustrating the distribution of *Irf7* expression in microglia. **H** Violin plots showing *Irf7* expression across individual microglia subclusters in sham and pdMCAO groups. **I** RT-qPCR quantification of *Irf7* mRNA expression at different time points after PTI. *n* = 4 mice per group. **J** Dot plot showing GO enrichment analysis comparing *Irf7*^*high*^ vs. *Irf7*^*low*^ microglia within the MG1 subcluster. ^*^P < 0.05, ^***^P < 0.001 vs. sham group in (**I**), one-way ANOVA with Tukey’s post hoc test. Data are presented as mean ± SEM

To explore the potential association between IRF7 and lipophagy, we reanalyzed the publicly available scRNA-seq dataset derived from mouse brain samples at day 14 after pdMCAO following QC (Fig. S5). UMAP analysis of 15,560 cells from six mice revealed nine major cell clusters, including astrocytes (AS), epithelial cells (Epi), endothelial cells (EC), fibroblasts, neurons, microglia (MG), lymphocytes (Lym), oligodendrocytes (Oligo) and pericytes (Fig. 2A, C), on the basis of established marker gene expression. Notably, the relative abundance of each cell cluster changed poststroke, with a marked increase in the proportion of MG from 22.2% to 34.9% (Fig. 2B).

Furthermore, the microglia were reclustered, and six subclusters (MG0–MG5) were identified (Fig. 2D). MG0 was characterized by the expression of immediate early genes (*Jun*, *Ier5*, *Junb*, *Egr1*, and *Fos*). MG1 was enriched for genes associated with disease-associated microglia (DAMs) (*B2m*, *Cst7*, *Ctsb*, *Lpl*, and *Spp1*) [44, 45]. MG2 expressed homeostatic genes (*Hexb*, *P2ry12*, *Tmem119*, *Siglech*, and *P2ry13*), which collectively maintain the resting and immunosurveillance functions of microglia [46]. MG3 expressed genes related to inflammatory regulation (*Lyz2*, *Ms4a7*, *Cybb*, *Ctsc*, and *Igf1*), MG4 expressed genes involved in gliogenesis (*Plp1*, *Aplp1*, *Mal*, *Cnp*, and *Gpm6b*), and MG5 expressed antigen-presentation-associated genes (*Cd74*, *H2-Aa*, *H2-Eb1*, *H2-Ab1*, and *H2-DMa*) (Fig. 2F) [47]. The relative abundance of these microglial subclusters markedly changed after stroke. In the sham group, microglia were predominantly composed of MG0 and MG2. In contrast, the proportions of MG0 and MG2 were markedly reduced in the pdMCAO group, accompanied by a significant increase in the MG1 subcluster, whereas no significant changes were observed in the MG3, MG4, or MG5 subclusters (Fig. 2E).

To characterize the expression pattern of *Irf7* in microglial subclusters poststroke, further analysis revealed that *Irf7* was most abundant in the MG1 subcluster (Fig. 2D, G) and was significantly upregulated in the pdMCAO group within this subcluster (Fig. 2H). Consistently, our results revealed that *Irf7* mRNA levels began to increase as early as day 1 after PTI, peaked at day 7, remained elevated at day 14 and subsequently declined (Fig. 2I). Then, MG1 cells were stratified into *Irf7*^*high*^ and *Irf7*^*low*^ groups, and GO enrichment analysis was performed on the genes that were differentially expressed between the two groups (Additional file 4). *Irf7*^*high*^ cells were enriched in biological processes (BPs), including selective autophagy, regulation of autophagy, regulation of autophagosome maturation, lipoprotein biosynthetic process and autophagosome assembly. Enrichment in cellular components (CCs), such as lipid droplet and autophagosome membrane was also observed (Fig. 2J). Similarly, we obtained consistent results in the reanalysis of another dataset from mouse brain samples collected at 14 days after tMCAO/R (Fig.S6, Additional file 5). Collectively, these results suggest that the *Irf7*^*high*^ microglial subcluster within MG1 is closely associated with poststroke alterations in autophagy and lipid metabolism, providing a rationale for the subsequent investigation of the role of microglial IRF7 in lipophagy following ischemic stroke.

### Microglial *Irf7* deficiency reduces LD accumulation by alleviating lipophagy impairment following ischemic stroke

**Fig. 3:**
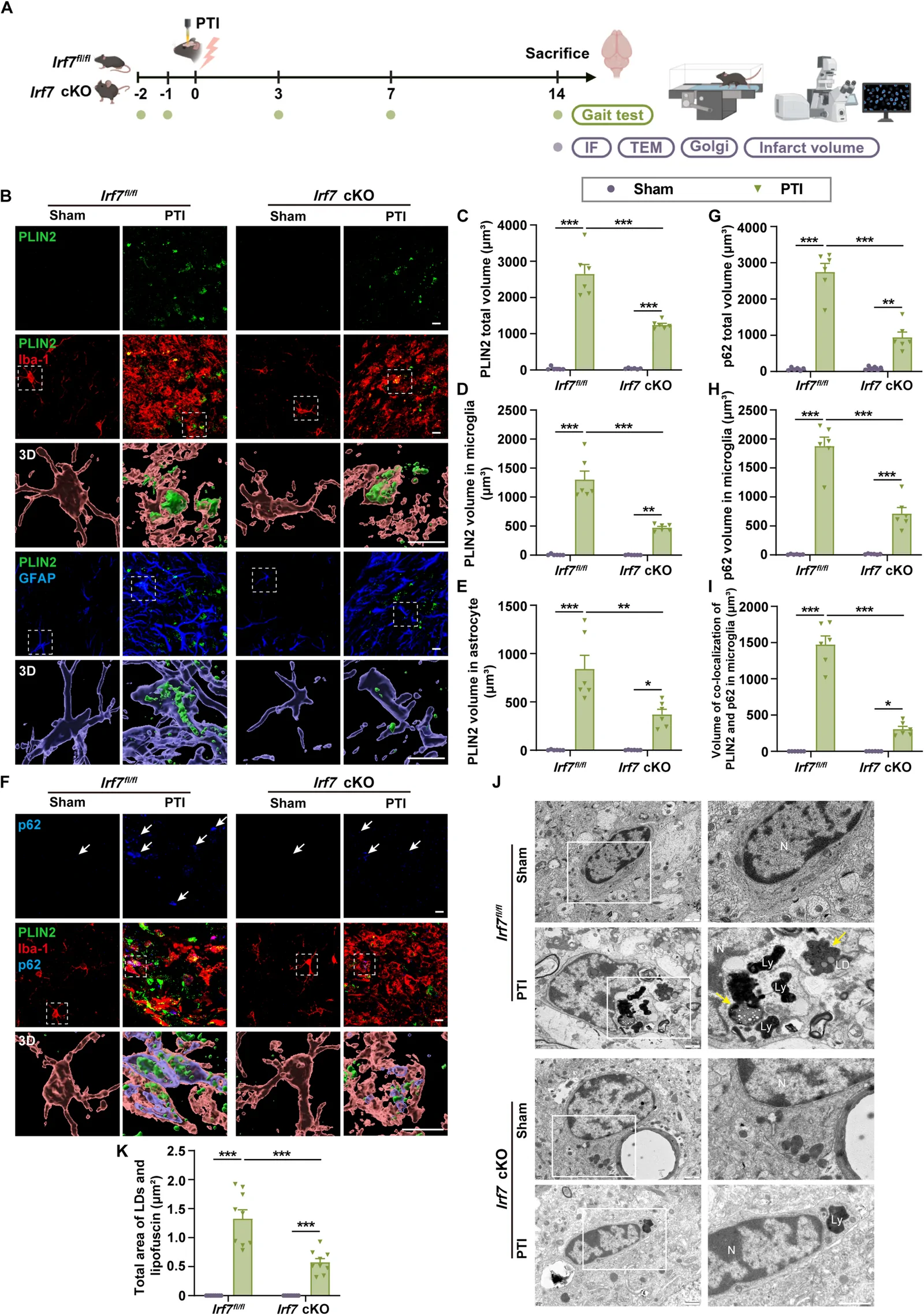
Microglial *Irf7* deficiency reduces LD accumulation by alleviating lipophagy impairment following ischemic stroke.** A** Schematic illustration of the experimental design for *Irf7*^*fl/fl*^ and *Irf7* cKO mice. **B** Representative immunofluorescence images showing colocalization of PLIN2 (LDs) with Iba-1 (microglia) and GFAP (astrocytes) in the peri-infarct region from *Irf7*^*fl/fl*^ and *Irf7* cKO mice at day 14 after PTI, along with magnified 3D-reconstructed images. Scale bar: 10 μm. **C**-**E** Quantification of PLIN2 total volume and its volume separately in microglia and astrocytes. *n* = 6 mice per group. **F** Representative immunofluorescence images showing colocalization of p62 and PLIN2 in microglia, along with magnified 3D-reconstructed images. Scale bars: 10 μm. **G**-**I** Quantification of p62 total volume, p62 volume in microglia, and the colocalization volume of PLIN2 and p62 in microglia. *n* = 6 mice per group. **J** Representative TEM images captured at both low and high magnification. Lipofuscin (yellow arrow), lipid droplet (LD) and lysosome (Ly). Scale bar: 1 μm. **K** Quantification of the total area of LDs and lipofuscin. *n* = 9 cells from 3 mice per group. ^*^P < 0.05, ^**^P < 0.01, ^***^P < 0.001, two-way ANOVA with Tukey’s post hoc test. Data are presented as mean ± SEM. (**A**) was created with BioRender.com

To investigate whether microglial IRF7 is a critical regulator of lipophagy after ischemic stroke, *Irf7*^*fl/fl*^ and *Irf7* cKO were utilized (Fig. 3A). Compared with *Irf7*^*fl/fl*^ littermate controls, *Irf7* cKO mice exhibited a marked reduction in PLIN2 total volume within the peri-infarct cortex at day 14 poststroke (Fig. 3B, C). Moreover, microglial *Irf7* deletion significantly decreased the PLIN2 volume in both Iba-1^+^ microglia and GFAP^+^ astrocytes (Fig. 3B, D, E), and also reduced the number, total volume, and average volume per cell of glial cells (Fig. S7A–F). Furthermore, microglial lipophagy was assessed by the colocalization of the major LD protein PLIN2 with the autophagy substrate p62, where increased colocalization indicates impaired lipophagic flux [48]. *Irf7* cKO mice showed a significant reduction in p62 total volume (Fig. 3F, G), along with decreased p62 accumulation and PLIN2–p62 colocalization in Iba-1^+^ microglia at day 14 poststroke (Fig. 3F, H, I). Consistently, TEM confirmed that the area of free LDs and lipofuscin within microglia was significantly reduced in *Irf7* cKO mice at day 14 poststroke (Fig. 3J, K). These results suggest that microglial IRF7 aggravates LD accumulation by exacerbating lipophagy dysfunction following ischemic stroke, whereas its deficiency alleviates this pathological process.

### Microglial *Irf7* deficiency enhances synaptic plasticity and promotes motor function recovery following ischemic stroke

**Fig. 4:**
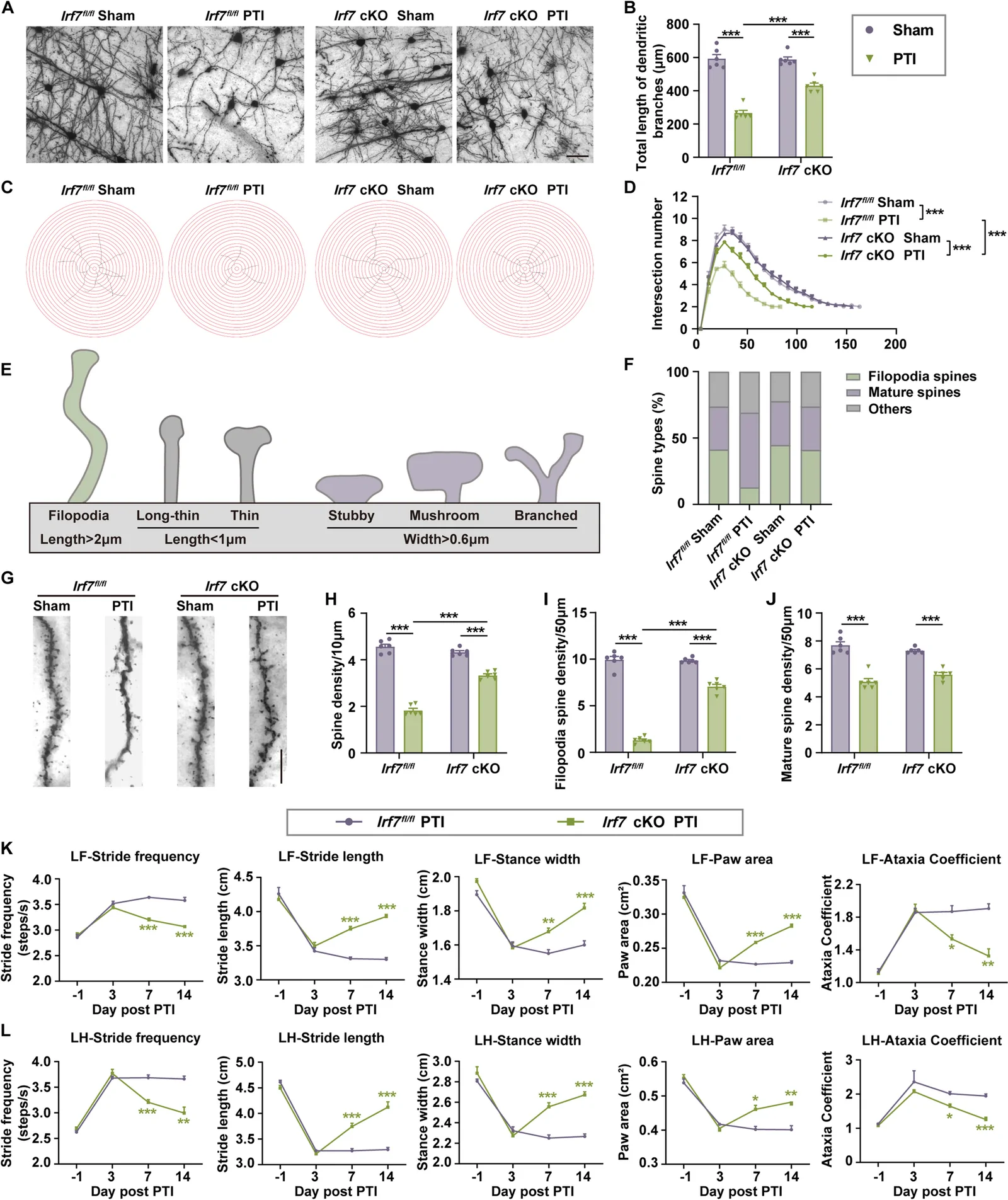
Microglial *Irf7* deficiency enhances synaptic plasticity and promotes motor function recovery following ischemic stroke. **A** Representative images of neuronal dendrites. Scale bar: 50 μm. **B** Quantification of total dendritic length. *n* = 6 mice per group. **C** Representative dendritic tracings used for Sholl analysis. **D** Quantification of intersection number. *n* = 6 mice per group. **E** Schematic illustration of dendritic spine types. **F** The percentage of different spine types. **G** Representative images of dendritic spines. Scale bar: 10 μm. **H**-**J** Quantification of spine density, filopodia-like spine density and mature spine density. *n* = 6 mice per group. **K** Gait test of the left forelimb (LF), including stride frequency, stride length, stance width, paw area and ataxia coefficient. *n* = 6 mice per group. **L** Gait test of the left hindlimb (LH), including stride frequency, stride length, stance width, paw area and ataxia coefficient. *n* = 6 mice per group. ^*^P < 0.05, ^**^P < 0.01, ^***^P < 0.001 in (**B**), (**D**) and (**H**)-(**J**), two-way ANOVA with Tukey’s post hoc test. ^*^P < 0.05, ^**^P < 0.01, ^***^P < 0.001 vs. *Irf7*^*fl/fl*^ PTI group in (**K**) and (**L**), two-way ANOVA with Bonferroni’s post hoc test. Data are presented as mean ± SEM

Given that reduced LD accumulation is beneficial for functional recovery after ischemic stroke, we performed Golgi-Cox staining and gait tests to investigate the effects of microglial IRF7 on synaptic plasticity and motor function. Golgi-Cox staining revealed that compared with sham mice, *Irf7*^*fl/fl*^ mice exhibited significantly reduction in total dendritic branch length (Fig. 4A, B), intersection number (Fig. 4C, D), and dendritic spine density (Fig. 4G, H) within the peri-infarct cortex at day 14 after PTI. However, these parameters markedly increased in *Irf7* cKO mice postinjury. Interestingly, compared with *Irf7*^*fl/fl*^ mice, *Irf7* cKO mice showed no significant change in the density of mushroom-type (mature) spines after stroke (Fig. 4E–G, J), whereas the density of filopodia-like (immature) spines markedly increased (Fig. 4E–G, I), a feature considered indicative of neuronal growth and synaptic remodeling [49, 50]. Moreover, *Irf7* cKO mice exhibited significantly improved motor outcomes of the left forelimb and left hindlimb compared with *Irf7*^*fl/fl*^ mice at days 7–14 after PTI, as indicated by decreased stride frequency and ataxia coefficient and increased stride length, stride width, and paw area, whereas no significant difference was observed at day 3 (Fig. 4K, L). Notably, this functional improvement was accompanied by a smaller infarct volume in *Irf7* cKO mice than in their littermate controls at 14 days after PTI (Fig. S7G). Similarly, we further validated the key findings in 15-month-old mice using the dMCAO model (Fig. S8A, B). The results showed that, compared with littermate controls, microglial *Irf7* deletion significantly alleviated the PLIN2 volume and the colocalization volume of PLIN2 and p62 in microglia within the peri-infarct region at 14 days after dMCAO, improved neurological function, shown as decreased neurological deficit scores and improved gait test performance, and also reduced infarct volume after dMCAO (Fig. S8C–I). These results indicate that microglial IRF7 hinders synaptic plasticity and motor function recovery during the subacute phase of ischemic stroke, which can be attributed, at least in part, to microglial IRF7-mediated impairment of lipophagy and LD accumulation.

### *Irf7* knockdown in microglia alleviates LPS-induced LD accumulation by restoring lipophagy

To substantiate these results, we carried out complementary in vitro experiments in BV2 cells and primary microglia cultures. First, BV2 cells were treated with LPS at various concentrations and time points, and LD accumulation, autophagic activity and IRF7 expression were evaluated. In an LPS inflammation model, LD accumulation (the number and size of LDs and the percentage of BODIPY⁺ cells) (Fig. S9A–D), the expression of autophagy-related proteins (beclin 1, the LC3Ⅱ/Ⅰ ratio, LC3Ⅱ and p62) (Fig. S9F, H–K), and both *Irf7* mRNA and IRF7 protein expression (Fig. S9E–G) increased in a dose- and time-dependent manner. On the basis of these results, 1000 ng/mL LPS for 24 h was selected for subsequent experiments.

**Fig. 5:**
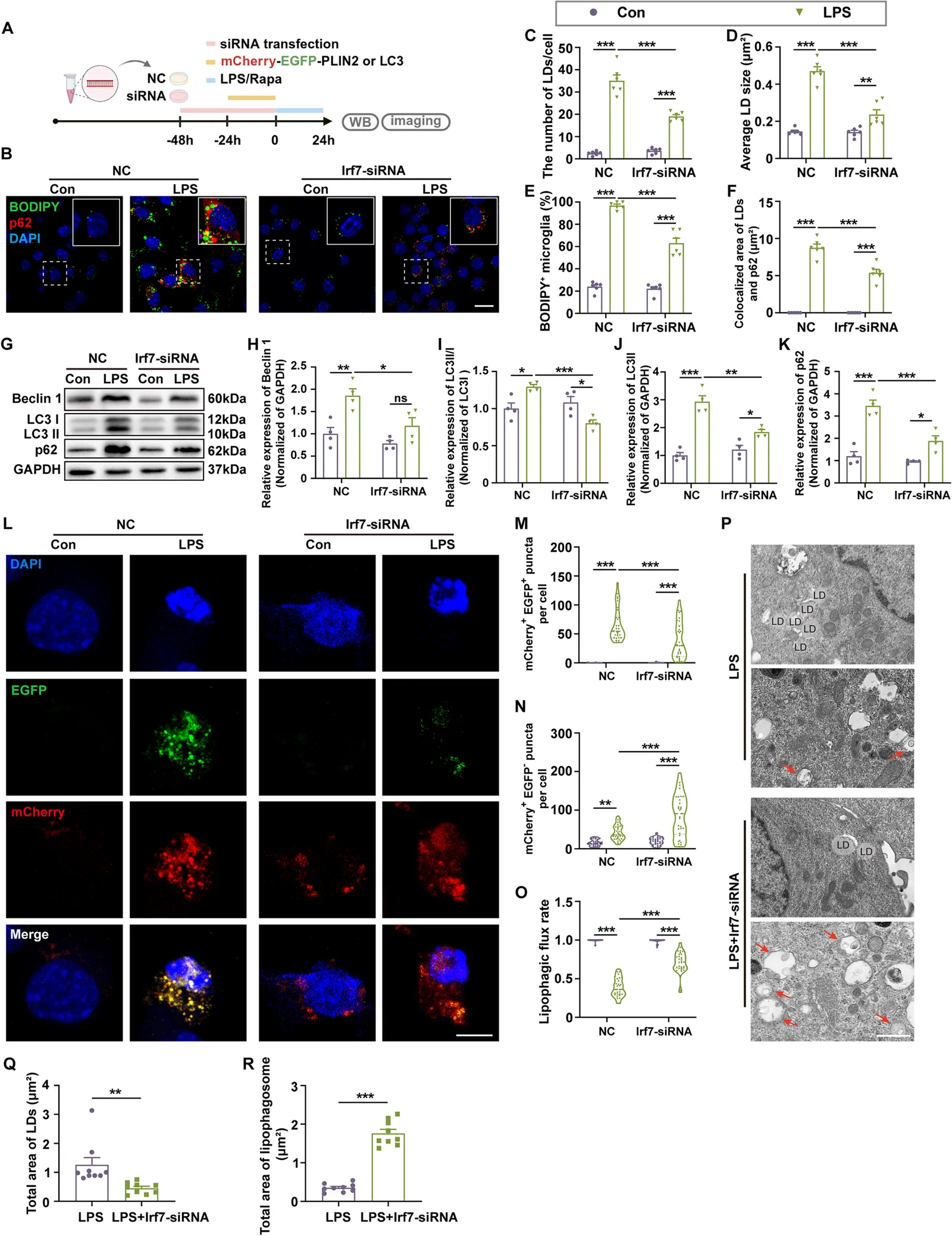
*Irf7* knockdown in microglia alleviates LPS-induced LD accumulation by restoring lipophagy.** A** Schematic illustration of BV2 cell treatments at different time points, as shown in Figs. 5, 6 and Fig. S11. **B** Representative fluorescence images showing the colocalization of BODIPY and p62 in BV2 cells following *Irf7* knockdown. Scale bar: 20 μm. **C**-**F** Quantification of LD number, size, the percentage of BODIPY⁺ cells and the colocalization area of BODIPY and p62. *n* = 6 biologically independent cultures per group. **G** Representative western blot images of beclin 1, LC3Ⅱ/Ⅰ, p62 and GAPDH. **H**-**K** Quantification of beclin 1, LC3Ⅱ/Ⅰ ratio, LC3Ⅱ and p62 levels, normalized to the NC-Con group. *n* = 4 biologically independent cultures per group. **L** Representative images of BV2 cells transfected with mCherry-EGFP-PLIN2. Scale bar: 20 μm. **M**-**O** Quantification of mCherry^+^EGFP^+^ puncta, mCherry^+^EGFP^−^ puncta and lipophagic flux rate. *n* = 30 cells from 3 biologically independent experiments per group. **P** Representative TEM images of LPS-stimulated BV2 cells following *Irf7* knockdown. Lipophagosome (red arrow) and lipid droplet (LD). Scale bars: 1 μm. **Q**,** R** Quantification of the area of LDs and lipophagosome. *n* = 9 from 3 biologically independent cultures per group. ^*^P < 0.05, ^**^P < 0.01, ^***^P < 0.001 in (**C**)-(**F**), (**H**)-(**K**), (**M**)-(**O**), two-way ANOVA with Tukey’s post hoc test. ^**^P < 0.01, ^***^P < 0.001 in (**Q**) and (**R**), unpaired t test. Data are presented as mean ± SEM. (**A**) were created with BioRender.com

The schematic of the BV2 cell treatments is presented in Fig. 5A. Three siRNA sequences targeting *Irf7* were transfected into BV2 cells, among which the si-3 sequence resulted in the most effective knockdown (Fig. S10A–C), and was used for subsequent experiments. We found that LPS stimulation significantly increased LD accumulation and the colocalized area of LDs and p62, whereas *Irf7* knockdown markedly attenuated these changes (Fig. 5B–F). We subsequently investigated the role of IRF7 in the autophagy level. We found that LPS stimulation led to increases in the beclin 1 level, LC3Ⅱ/Ⅰ ratio, LC3Ⅱ and p62 expression, whereas *Irf7*-siRNA treatment significantly reduced these parameters (Fig. 5G–K). Then, BV2 cells were transfected with the PLIN2-mCherry-EGFP adenovirus. In LPS-stimulated BV2 cells, both mCherry⁺EGFP⁺ and mCherry⁺EGFP⁻ puncta markedly increased, accompanied by a significant reduction in the lipophagic flux rate; in contrast, *Irf7*-siRNA treatment decreased mCherry⁺EGFP⁺ puncta, and increased mCherry⁺EGFP⁻ puncta and the lipophagic flux rate (Fig. 5L–O). TEM revealed that LPS stimulation increased the area of free LDs and decreased the area of lipophagosomes, suggesting impaired lipophagy, which was alleviated by *Irf7*-siRNA (Fig. 5P–R). In addition, similar results were observed in isolated primary microglial cultures, where *Irf7*-siRNA alleviated LPS-induced LD accumulation and reduced the colocalized area of LDs and p62 (Fig. 7A–G). Moreover, we transduced BV2 cells with an LC3-mCherry-EGFP adenovirus. The results showed that rapamycin treatment reduced LD accumulation by alleviating autophagic flux blockade, with an effect comparable to that of *Irf7*-siRNA (Fig. S11). These results suggest that IRF7 inhibition alleviates LPS-induced lipophagy impairment and LD accumulation *in vitro*. This finding aligns with the beneficial effects of microglial *Irf7* deficiency following ischemic stroke.

### IRF7 drives *Gnai2* transcription to reduce PC levels and impair lipophagy in microglia

**Fig. 6:**
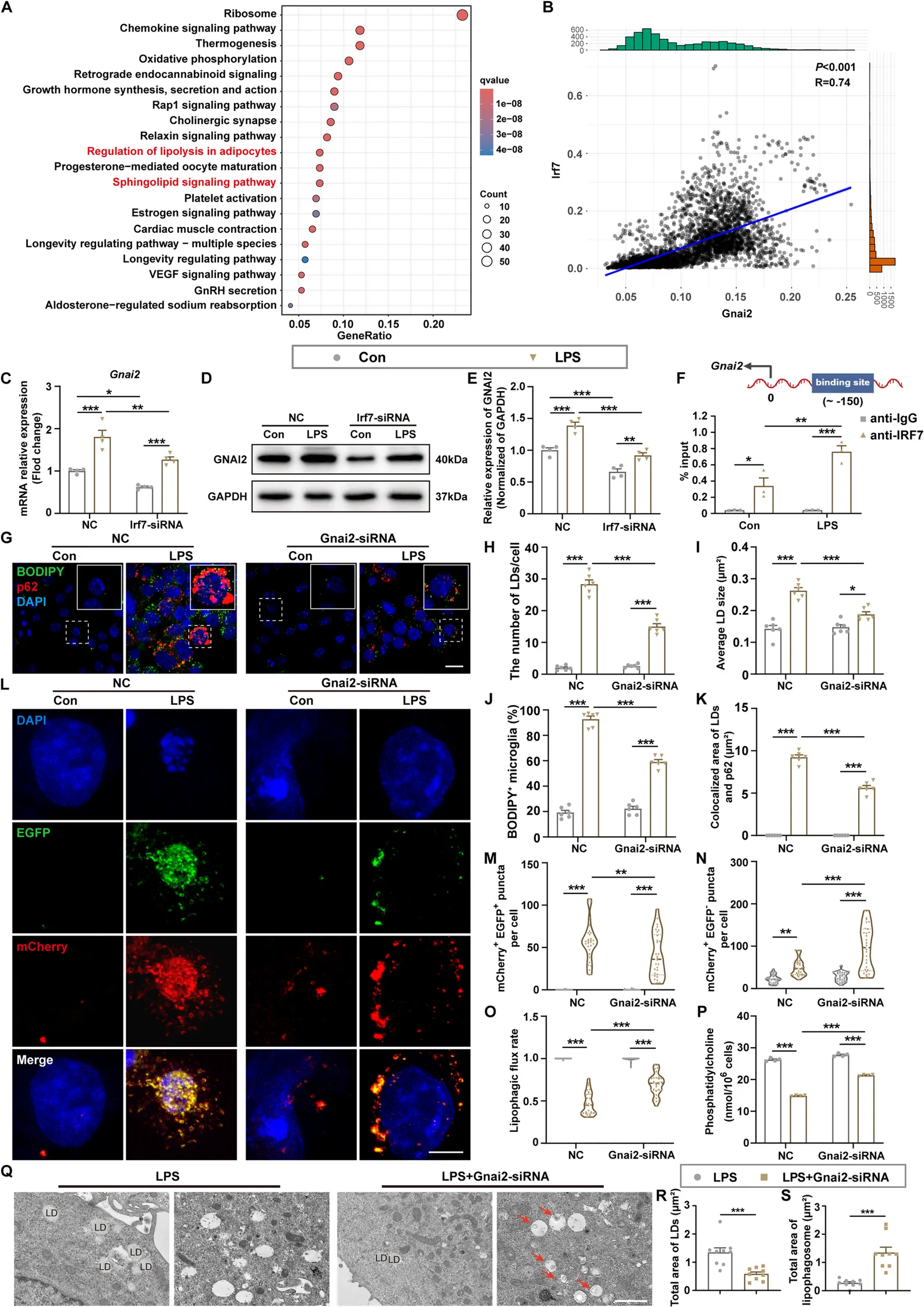
IRF7 drives *Gnai2* transcription to reduce PC levels and impair lipophagy in microglia.** A** Dot plot showing KEGG pathway enrichment of all genes positively correlated with *Irf7* in the MG1 subcluster based on scRNA-seq analysis. **B** Scatter plot showing the positive correlation between *Irf7* and *Gnai2* expression within the MG1 subcluster. **C** Quantification of *Gnai2* mRNA expression of BV2 cells following *Irf7* knockdown. *n* = 4 biologically independent cultures per group. **D** Representative western blot images of GNAI2 and GAPDH. **E** Quantification of GNAI2 levels, normalized to the NC-Con group. *n* = 4 biologically independent cultures per group. **F** ChIP-qPCR analysis showing the enrichment levels of IRF7 at the *Gnai2* gene promoter in BV2 cells, with IgG as a negative control. Data represent the percent of input. *n* = 3 biologically independent cultures per group. **G** Representative fluorescence images showing the colocalization of BODIPY and p62 in BV2 cells following *Gnai2* knockdown. Scale bar: 20 μm. **H**-**K** Quantification of LD number, size, the percentage of BODIPY⁺ cells and the colocalization area of BODIPY and p62. *n* = 6 biologically independent cultures per group. **L** Representative images of BV2 cells transfected with mCherry-EGFP-PLIN2. Scale bar: 20 μm. **M**-**O** Quantification of mCherry^+^EGFP^+^ puncta, mCherry^+^EGFP^−^ puncta and lipophagic flux rate. *n* = 30 cells from 3 biologically independent experiments per group. **P** The PC levels of BV2 cells following *Gnai2* knockdown. *n* = 4 biologically independent cultures per group. **Q** Representative TEM images of LPS-stimulated BV2 cells following *Gnai2* knockdown. Lipophagosome (red arrow) and lipid droplet (LD). Scale bars: 1 μm. **R**,** S** Quantification of the area of LDs and lipophagosome. *n* = 9 from 3 biologically independent cultures per group. ^*^P < 0.05, ^**^P < 0.01, ^***^P < 0.001 in (**C**), (**E**), (**F**), (**H**)-(**K**) and (**M**)-(**P**), two-way ANOVA with Tukey’s post hoc test. ^***^P < 0.001 in (**R**) and (**S**), unpaired t test. Data are presented as mean ± SEM. (**F**) was created with BioRender.com

**Fig. 7:**
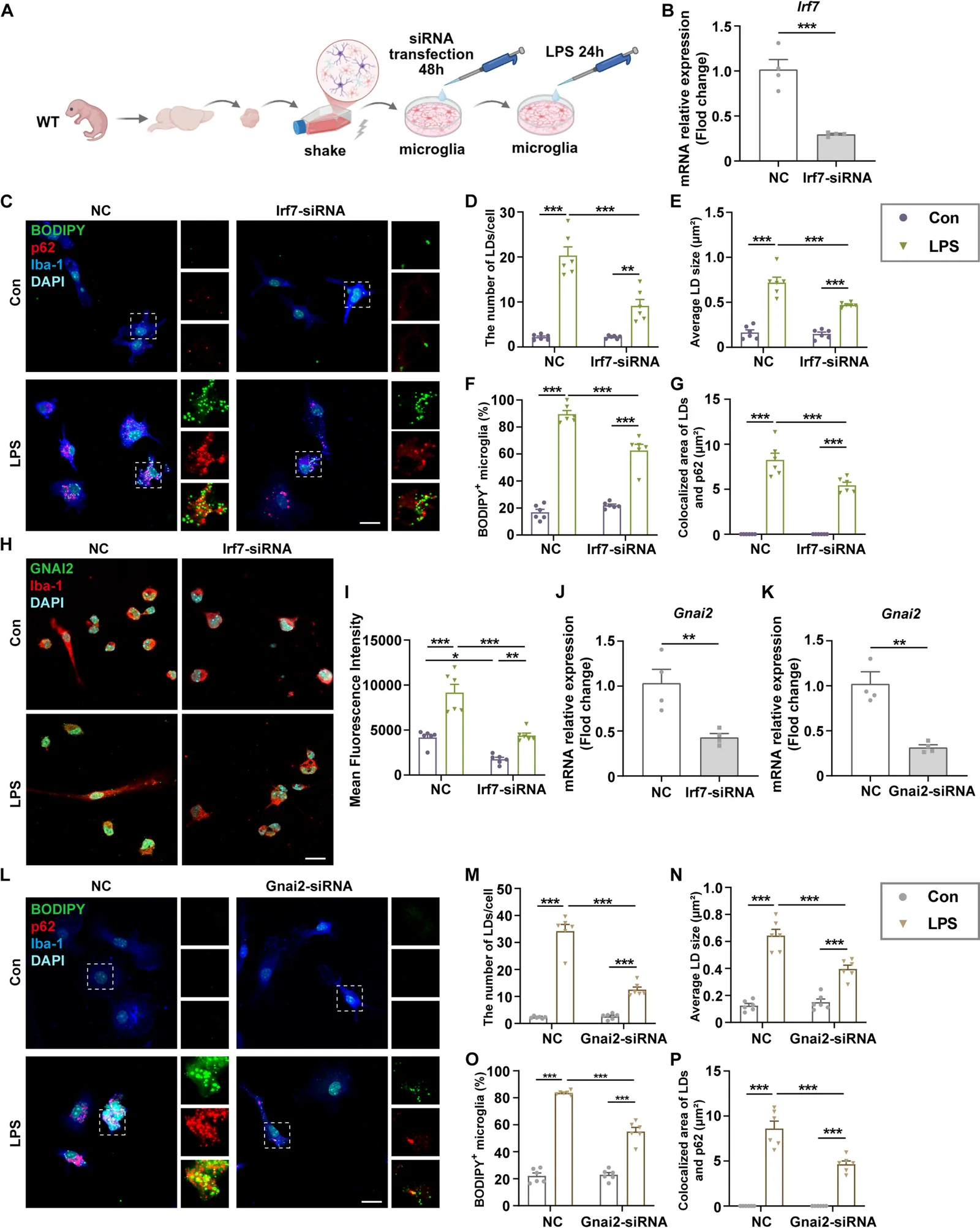
*Irf7* or *Gnai2* knockdown in primary microglia alleviates LPS-induced LD accumulation by restoring lipophagy.** A** Schematic illustration of the treatment of primary microglia. **B** Validation of *Irf7* knockdown efficiency. *n* = 4 biologically independent cultures per group. **C** Representative fluorescence images showing the colocalization of BODIPY and p62 in primary microglia following *Irf7* knockdown. Scale bar: 20 μm. **D**-**G** Quantification of LD number, size, the percentage of BODIPY⁺ cells and the colocalization area of BODIPY and p62. *n* = 6 biologically independent cultures per group. **H** Representative fluorescence images showing the GNAI2 mean fluorescence intensity. **I** Quantification of the GNAI2 mean fluorescence intensity. *n* = 6 biologically independent cultures per group. **J** *Gnai2* mRNA expression after *Irf7* knockdown. *n* = 4 biologically independent cultures per group. **K** Validation of *Gnai2* knockdown efficiency. *n* = 4 biologically independent cultures per group. **L** Representative fluorescence images showing the colocalization of BODIPY and p62 in primary microglia following *Gnai2* knockdown. Scale bar: 20 μm. **M**-**P** Quantification of LD number, size, the percentage of BODIPY⁺ cells and the colocalization area of BODIPY and p62. *n* = 6 biologically independent cultures per group. ^**^P < 0.01, ^***^P < 0.001 in (**B**), (**J**) and (**K**), unpaired t test. ^**^P < 0.01, ^***^P < 0.001 in (**D**)-(**G**), (**I**) and (**M**)-(**P**), two-way ANOVA with Tukey’s post hoc test. Data are presented as mean ± SEM. (**A**) were created with BioRender.com

Given the pivotal role of IRF7 in lipophagy impairment, identifying its downstream targets is essential for elucidating its underlying molecular mechanisms. Through reanalysis of the scRNA-seq dataset, we identified 416 genes within the MG1 subcluster that were strongly positively correlated with *Irf7* expression. KEGG enrichment analysis revealed that *Gnai2* was strongly associated with lipid metabolism pathways (Fig. 6A, B, Fig. S12). In addition, *Irf7* knockdown in BV2 cells reduced LPS-induced *Gnai2* mRNA and GNAI2 protein expression (Fig. 6C–E). Notably, the ChIP‒qPCR results revealed marked enrichment of IRF7 binding at the promoter region of the *Gnai2* gene, indicating that *Gnai2* is a direct transcriptional target of IRF7 (Fig. 6F). It has been reported that GNAI2 suppresses PC synthesis, thereby leading to impaired lipophagy in the liver [51]. Reduced PC levels promote the coalescence of small LDs into larger ones, hinder autophagosome formation and disrupt autophagic flux [52, 53]. To further investigate the role of *Gnai2*, this gene was knocked down in BV2 cells, and the si-3 sequence resulted in the most effective knockdown (Fig. S10D–F). *Gnai2* knockdown markedly reduced LPS-induced LD accumulation and the colocalized area of LDs and p62 (Fig. 6G–K), alleviated lipophagic flux blockade (Fig. 6L–O) and enhanced PC levels (Fig. 6P). TEM revealed that LPS stimulation increased the area of free LDs and decreased the area of lipophagosomes, which was alleviated by *Gnai2*-siRNA (Fig. 6Q–S). In addition, *Irf7* knockdown in primary microglia significantly reduced both the mRNA expression level of *Gnai2* and the mean fluorescence intensity of GNAI2 (Fig. 7H–J). *Gnai2* knockdown in primary microglia alleviated LPS-induced LD accumulation and reduced the colocalized area of LDs and p62 (Fig. 7K–P).

Moreover, *Irf7* knockdown in BV2 cells attenuated the LPS-induced reduction in PC levels (Fig. S13A). Meanwhile, and *Gnai2* mRNA levels remained elevated at days 3–14 after PTI (Fig. S13B), and PC levels were significantly reduced at day 14 poststroke, and this decrease was markedly reversed in *Irf7* cKO mice (Fig. S13C). More importantly, we administered CDP-choline, an intermediate in PC biosynthesis, every other day from day 3 to day 14 after PTI (Fig. S13D). The results showed that this intervention significantly restored PC levels in the peri-infarct region, alleviated the PLIN2 volume and the colocalization volume of PLIN2 and p62 in microglia, and improved motor function recovery at day 14 after PTI (Fig. S13E–J). These results suggest that microglial *Irf7* deletion elevates PC levels by suppressing *Gnai2* transcription, thereby alleviating the impairment of lipophagy and ultimately reducing LD accumulation.

### Delayed STING inhibitor administration alleviates lipophagy impairment and promotes functional recovery following ischemic stroke

**Fig. 8:**
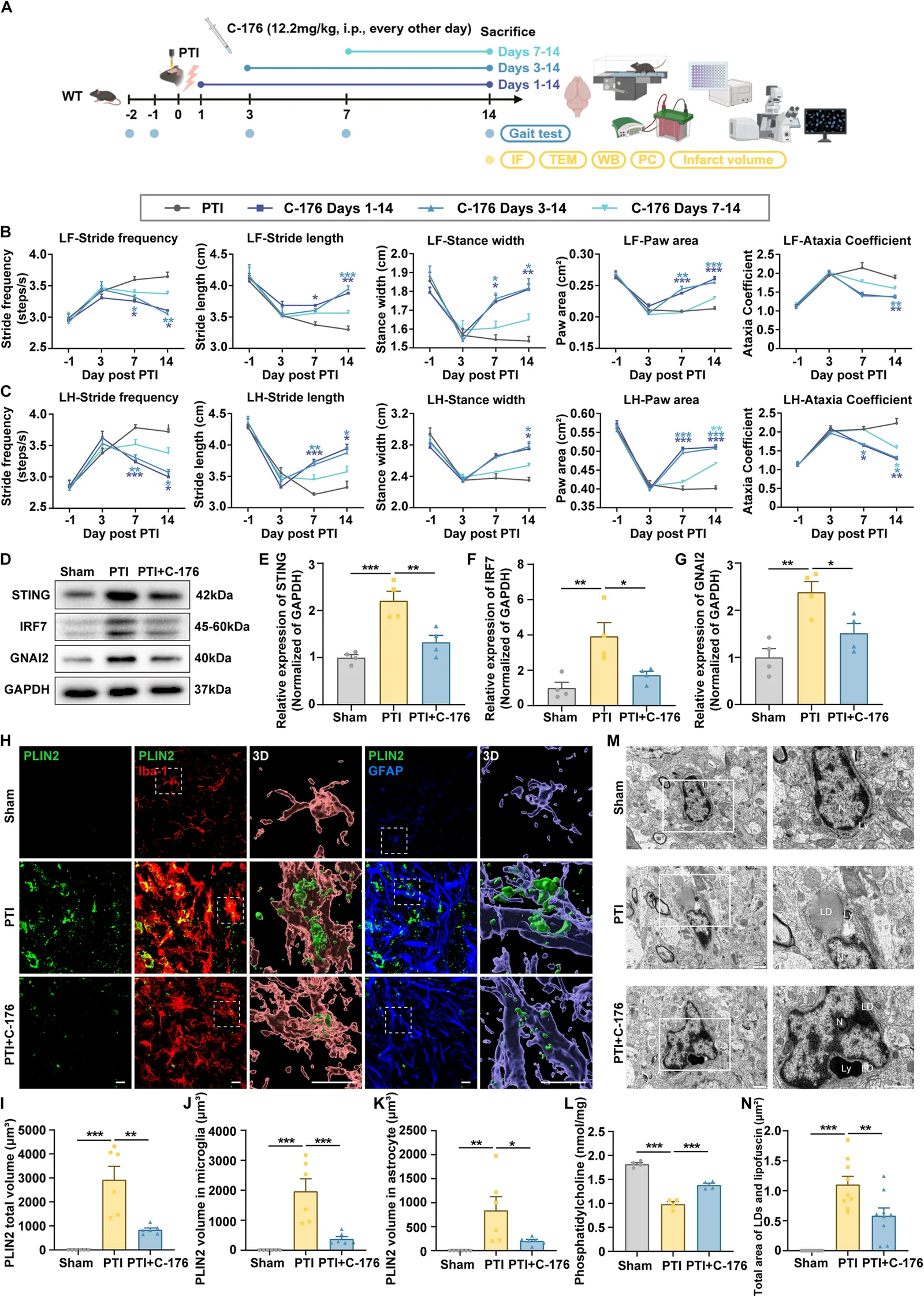
Delayed STING inhibitor administration alleviates lipophagy impairment and promotes functional recovery following ischemic stroke. **A** Schematic illustration of the experimental design for C-176 administration in WT mice. **B** Gait test of the left forelimb (LF), including stride frequency, stride length, stance width, paw area and ataxia coefficient. *n* = 6 mice per group. **C** Gait test of the left hindlimb (LH), including stride frequency, stride length, stance width, paw area and ataxia coefficient. *n* = 6 mice per group. **D** Representative western blot images of STING, IRF7, GNAI2 and GAPDH. **E**-**G** Quantification of STING, IRF7 and GNAI2 levels, normalized to the sham group. *n* = 4 mice per group. **H** Representative immunofluorescence images showing colocalization of PLIN2 (LDs) with Iba-1 (microglia) and GFAP (astrocytes) in the peri-infarct region from WT mice at day 14 after PTI, along with magnified 3D-reconstructed images. Scale bars: 10 μm. **I**-**K** Quantification of PLIN2 total volume and its volume separately in microglia and astrocytes. *n* = 6 mice per group. **L** The PC levels in WT mice at day 14 after PTI. *n* = 4 mice per group. **M** Representative TEM images captured at both low and high magnification. lipid droplet (LD) and lysosome (Ly). Scale bars: 1 μm. **N** Quantification of the total area of LDs and lipofuscin. *n* = 9 cells from 3 mice per group. ^*^P < 0.05, ^**^P < 0.01, ^***^P < 0.001 vs. PTI group in (**B**) and (**C**), two-way ANOVA with Tukey’s post hoc test. ^*^P < 0.05, ^**^P < 0.01, ^***^P < 0.001 in (**E**)-(**G**), (**I**)-(**L**) and (**N**), one-way ANOVA with Tukey’s post hoc test. Data are presented as mean ± SEM. (**A**) was created with BioRender.com

To achieve pharmacological inhibition of IRF7, we used C-176, a selective inhibitor of its upstream target STING. We first examined the spatiotemporal expression of STING in the peri-infarct cortex following ischemic stroke. STING expression peaked between days 3 and 14 after PTI and was specifically localized in microglia rather than astrocytes (Fig. S14). To determine the therapeutic time window of the STING inhibitor C-176, intraperitoneal injections were initiated at days 1, 3, or 7 after PTI, and the drugs were administered every other day until day 14 (Fig. 8A). Gait analysis revealed that the C-176 days 3–14 group had significantly improved motor outcomes, with effects comparable to those observed in the C-176 days 1–14 group (Fig. 8B, C). In contrast, the C-176 days 7–14 group exhibited modest functional improvement in certain motor parameters (Fig. 8B, C), which may be attributable to the reduced dosing frequency. Meanwhile, both the C-176 days 1–14 group and the C-176 days 3–14 group significantly reduced infarct volume after PTI, whereas the C-176 days 7–14 group showed only a mild reduction (Fig. S15A). These findings suggest that the effective therapeutic window for C-176 extends to at least 3–7 days poststroke. On the basis of these results, C-176 was administered from days 3 to 14 poststroke to further investigate its potential functions. We found that C-176 treatment markedly reduced the protein expression levels of STING, GNAI2, IRF7, p-IRF7, TBK1 and p-TBK1 at day 14 after PTI (Fig. 8D–G, Fig. S15B–H). Meanwhile, C-176 treatment reduced LD accumulation in both Iba-1^+^ microglia and GFAP^+^ astrocytes (Fig. 8H–K), decreased the number and volume of glial cells (Fig. S15I-N), and enhanced PC levels (Fig. 8L). TEM revealed that C-176 treatment reduced the area of free LDs and lipofuscin at day 14 after PTI (Fig. 8M, N). In addition, in vitro experiments using primary microglia (Fig. S16A–D) and BV2 cells (Fig. S16E–H) demonstrated that C-176 treatment significantly attenuated LPS-induced LD accumulation. These results suggest that delayed administration of the STING inhibitor C-176 after PTI improves motor performance and alleviates LD accumulation and lipophagic dysfunction, accompanied by the downregulation of IRF7 and GNAI2 expression and, importantly, the restoration of PC levels.

### Microglial IRF7 mediates the beneficial effects of the STING inhibitor C-176 following ischemic stroke

**Fig. 9:**
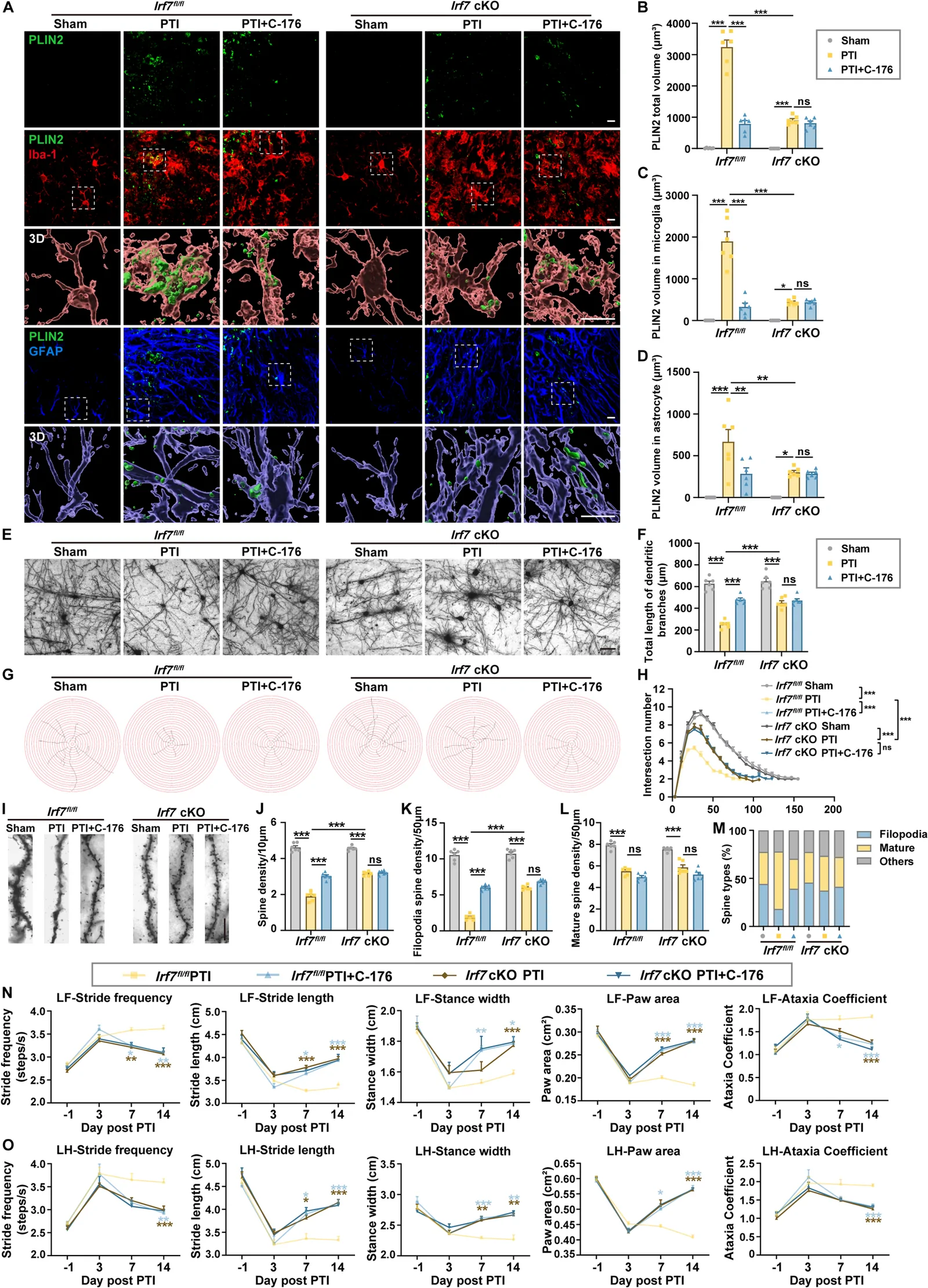
Microglial *Irf7* mediates the beneficial effects of the STING inhibitor C-176 following ischemic stroke.** A** Representative immunofluorescence images showing colocalization of PLIN2 (LDs) with Iba-1 (microglia) and GFAP (astrocytes) in the peri-infarct region from *Irf7*^*fl/fl*^ and *Irf7* cKO mice at day 14 after PTI, along with magnified 3D-reconstructed images. Scale bar: 10 μm. **B**-**D** Quantification of PLIN2 total volume and its volume separately in microglia and astrocytes. *n* = 6 mice per group. **E** Representative images of neuronal dendrites. Scale bar: 50 μm. **F** Quantification of total dendritic length. *n* = 6 mice per group. **G** Representative dendritic tracings used for Sholl analysis. **H** Quantification of intersection number. *n* = 6 mice per group. **I** Representative images of dendritic spines. Scale bar: 10 μm. **J**-**L** Quantification of spine density, filopodia-like spine density and mature spine density. *n* = 6 mice per group. **M** The percentage of different spine types. **N** Gait test of the left forelimb (LF), including stride frequency, stride length, stance width, paw area and ataxia coefficient. *n* = 6 mice per group. **O** Gait test of the left hindlimb (LH), including stride frequency, stride length, stance width, paw area and ataxia coefficient. *n* = 6 mice per group. ^*^P < 0.05, ^**^P < 0.01, ^***^P < 0.001 in (**B**)-(**D**), (**F**), (**H**) and (**J**)-(**L**), two-way ANOVA with Tukey’s post hoc test. ^*^P < 0.05, ^**^P < 0.01, ^***^P < 0.001 vs. *Irf7*^*fl/fl*^ PTI group in (**N**) and (**O**), two-way ANOVA with Tukey’s post hoc test. Data are presented as mean ± SEM

To further determine whether microglial IRF7 is a key target that mediates the effects of the STING inhibitor C-176, we treated *Irf7* cKO mice with C-176 after PTI. Consistent with the above findings, both delayed C-176 administration in *Irf7*^*fl/fl*^ mice and in untreated *Irf7* cKO mice markedly reduced LD accumulation within the peri-infarct region (Fig. 9A–D). However, C-176 treatment did not lead to any further reduction in LD burden in *Irf7* cKO mice compared with untreated *Irf7* cKO mice poststroke (Fig. 9A–D). Similar results were also observed in Golgi-Cox staining and gait behavioral analyses: delayed C-176 administration to *Irf7* cKO mice failed to further increase total dendritic length (Fig. 9E, F), intersection number (Fig. 9G, H), spine density (Fig. 9I, J) or the density of filopodia-like (immature) spines (Fig. 9K–M) within the peri-infarct cortex at day 14 after PTI. Similarly, no additional improvement in motor function was observed (Fig. 9N, O). Collectively, these results indicate that microglial IRF7 serves as a key target through which C-176 exerts its beneficial effects, and that pharmacological inhibition of STING fully recapitulates the therapeutic outcomes of microglial *Irf7* deletion.

## Discussion

Ischemic stroke triggers a cascade of complex metabolic and inflammatory responses that profoundly reprogram microglial function and the neuroinflammatory microenvironment. Increasing evidence indicates that aberrant LD turnover, particularly impaired lipophagy, represents a critical event contributing to disrupted lipid homeostasis and exacerbated neuroinflammation and neuronal injury [15, 54]. In this study, we demonstrated for the first time that microglial lipophagy is persistently impaired following ischemic stroke from the subacute phase to the chronic phase. This phenomenon partly explains the long-term LD accumulation observed in microglia. We reanalyzed publicly available scRNA-seq datasets from poststroke mouse brains and found that *Irf7* was predominantly upregulated in the microglial subcluster MG1. Further subdivision of MG1 into *Irf7*^*high*^ and *Irf7*^*low*^ subclusters followed by GO enrichment analysis revealed significant enrichment of genes related to autophagy regulation, lipid droplet and autophagosome membrane. Notably, the conditional deletion of microglial *Irf7* alleviated stroke-induced lipophagy impairment and LD accumulation in microglia, enhanced neuronal plasticity and promoted motor function recovery. Furthermore, we revealed that *Gnai2* was a key downstream target of IRF7 and disrupted PC metabolism in microglia. Collectively, these findings suggest that microglial IRF7 is a promising target for regulating abnormal lipid metabolism following ischemic stroke, thereby offering a novel strategy for promoting neurological recovery.

LD accumulation in microglia has been reported to persist for up to 90 days following MCAO within the ischemic hemisphere [12, 55–57]. LDAM exhibits a markedly proinflammatory profile during the subacute phase poststroke [12]. The inhibition of microglial LD formation with TrC (an acyl-CoA synthetase inhibitor) attenuated the OGD- or LPS-induced upregulation of proinflammatory cytokines and OGD/R-induced neural injury [12]. Moreover, the reduction in LDs within microglia was accompanied by enhanced motor function in the MCAO model [55, 57]. Similarly, we found that LD accumulation peaked between 3 and 14 days after PTI (corresponding to the subacute phase), particularly within microglia, and persisted into the chronic phase (up to 21 days). To provide direct evidence linking LD overload to functional recovery poststroke, we depleted cerebral LDs with TrC at 3–14 days poststroke, and found that it effectively improved motor function recovery as well as attenuated microglial overactivation and LD overload. Therefore, excessive accumulation of LDs from the subacute phase to the chronic phase poststroke serves as a critical barrier to functional recovery. Furthermore, the abnormal aggregation of LDs is strongly associated with impaired lipophagy [54]. Previous studies have shown that impaired lipophagy in macrophages during atherosclerosis leads to LD accumulation and accelerates disease progression [58–60]. Similarly, in diabetes-associated cognitive impairment and demyelination models, lipophagy dysfunction in microglia prevents effective LD degradation, resulting in excessive accumulation [10, 16]. However, whether microglial lipophagy is disrupted during the long-term recovery phase following ischemic stroke remains unclear. Here, we demonstrated that microglial lipophagy was persistently impaired from days 3 to 21 after PTI, as evidenced by LD accumulation, an increased colocalization volume of PLIN2 and p62, and the presence of free LDs and lipofuscin as observed by TEM. This dysfunction coincides temporally with the accumulation of LDs and represents a key contributing factor to LD aggregation. Therefore, restoring normal lipophagic activity in microglia is crucial for reducing LD overload. However, effective molecular targets and therapeutic strategies for modulating this process following ischemic stroke are lacking.

A series of evidences indicate that, in addition to its canonical role in immune regulation, IRF7 participates in the regulation of glucose and lipid metabolism, insulin resistance [19–22] and autophagy [23, 24] in peripheral tissues. Moreover, several omics-based datasets have consistently shown that *Irf7* expression is significantly elevated in microglia at multiple time points after MCAO, including 24 h [32], day 3 [33], day 4 [61] and day 7 [34]. Reanalysis of two publicly available scRNA-seq datasets showed that *Irf7* was highly expressed in the MG1 microglial subcluster at day 14 after both pdMCAO and tMCAO/R. Comparative analysis of *Irf7*^*high*^ versus *Irf7*^*low*^ microglia within the MG1 subcluster revealed significant enrichment in autophagy-related biological processes and in lipid droplet- and autophagosome membrane-associated cellular components. Meanwhile, *Irf7* expression remained persistently elevated throughout the subacute phase following PTI, and was temporally correlated with LD accumulation and lipophagic dysfunction, suggesting a potential pathological link between IRF7 and these processes. We further investigated the role of microglial IRF7 in regulating lipophagy after ischemic stroke. As an autophagic substrate, p62 can bind to LDs and deliver them to lysosomes for codegradation; thus, increased colocalization of p62 with LDs is commonly regarded as an indicator of impaired lipophagic flux [48]. In this study, marked increases in LD accumulation, p62–LD colocalization, lipofuscin deposition and free LDs were observed in microglia within the peri-infarct region after PTI. Notably, these parameters were significantly reduced in *Irf7* cKO mice. Similarly, in BV2 cells and primary microglia, *Irf7* knockdown effectively attenuated the LPS-induced increase in LD accumulation, the area of p62–LD colocalization, and impaired lipophagy flux. Furthermore, TEM revealed a reduction in free LDs accompanied by an increase in the area of lipophagosomes in LPS-stimulated BV2 cells following *Irf7* knockdown. These findings indicate that microglial IRF7 is a critical contributor to lipophagy impairment following ischemic stroke and that its inhibition can alleviate this dysfunction and diminish LD aggregation, which is consistent with our observations from the reanalysis of scRNA-seq datasets. Interestingly, a reduction in LDs was also observed in the astrocytes of *Irf7* cKO mice, suggesting that microglial IRF7 functions as a regulatory “leverage point” that reshapes the poststroke brain microenvironment. Additionally, at day 3 after PTI, *Irf7* cKO mice failed to show improvement in motor function, which is consistent with the findings of a previous study in which *Irf7* KO mice were used in the MCAO model [62]; however, *Irf7* cKO mice exhibited markedly improved motor function and enhanced synaptic plasticity at day 14 postinjury. The enhancement of synaptic plasticity in *Irf7* cKO mice was primarily manifested by increased dendritic branch length, intersections and dendritic spine density postinjury, particularly the number of filopodia-like spines, which are recognized as indicators of active synaptic remodeling and neuronal growth [49, 50]. In addition, we also observed beneficial effects in *Irf7* cKO mice in the 15-month-old dMCAO model. These findings indicated that the role of microglial IRF7 is broadly relevant to the pathogenesis of ischemic stroke, highlighting its translational potential as a therapeutic target for promoting recovery in clinically heterogeneous stroke populations. Notably, on the basis of our findings that reducing LD accumulation promotes motor function recovery, the beneficial effects of microglial *Irf7* deletion are produced, at least in part, through the restoration of lipophagic flux, which alleviates LD overload during the subacute phase of ischemic stroke.

Through the reanalysis of scRNA-seq data combined with ChIP‒qPCR, we identified *Gnai2* as a direct downstream target of IRF7, which plays an essential role in regulating lipid metabolism. G protein subunit alpha i2 (GNAI2) belongs to the inhibitory class of G protein α subunits and plays pivotal roles in insulin sensitivity, glucose metabolism and lipolysis [63, 64]. Notably, Zhang et al. reported that hepatic *Gnai2* deletion enhances PC synthesis and subsequently promotes lipophagy by facilitating the interaction between peroxiredoxin-1 and glycerophosphodiester phosphodiesterase domain-containing 5 [51]. PC is the most abundant phospholipid in cellular membranes, including those of LDs and autophagosome membranes [52, 53]. Previous studies have shown that PC plays a unique role in stabilizing LDs by acting as a surfactant that prevents small LDs from coalescing into larger ones, thereby facilitating their more efficient entry into the lipophagy pathway for degradation [53, 54]. On the other hand, disruption of PC *de novo* synthesis severely impairs autophagic flux, resulting in the accumulation of unsealed cup-shaped structures, whereas restoration of PC biosynthesis promotes autophagosome formation and autophagic flux [52, 65–67]. Therefore, PCs provide a shared structural and functional basis for LD stabilization and lipophagy, thereby coordinating lipophagic flux with LD clearance and ultimately preserving intracellular lipid homeostasis. Moreover, PC levels are reduced in patient serum during both acute and chronic poststroke stages, paralleling findings in rat brains one month after stroke [68–70], whereas exogenous PC supplementation has anti-inflammatory effects [71, 72]. In the PTI model, we detected a marked upregulation of *Gnai2* accompanied by reduced PC levels. In BV2 cells and primary microglia, *Gnai2* knockdown effectively alleviated the LPS-induced decrease in PC, LD accumulation, the area of p62–LD colocalization, and impaired lipophagy flux. Moreover, TEM revealed a reduction in free LDs accompanied by an increase in lipophagosomes in LPS-stimulated BV2 cells following *Gnai2* knockdown. Interestingly, the size of the LDs was also reduced, which may be attributed to the role of PCs in maintaining LD stability. Consistent with these findings, *Irf7* knockdown in LPS-stimulated BV2 cells reduced *Gnai2* expression and increased PC levels. Similarly, the PC levels in the peri-infarct tissue were also restored in *Irf7* cKO mice poststroke, and further beneficial effects were observed following exogenous supplementation with CDP-choline, an intermediate in PC biosynthesis, after in vivo PTI injury. Thus, the IRF7-mediated upregulation of GNAI2, which suppresses PC levels, serves as a key mechanism linking microglial IRF7 to impaired lipophagy and LD accumulation after ischemic stroke, ultimately disrupting intracellular lipid homeostasis. However, given that GNAI2 is ubiquitously expressed across various tissues [73, 74], and that PC is widely involved in cellular membrane composition, GNAI2-mediated regulation of PC may affect not only lipophagy but also other forms of autophagy in a non-specific manner, thereby influencing LD homeostasis. Direct targeting of GNAI2 may suppress its physiological functions in other cells or tissues, thereby causing unintended side effects. Since IRF7 is inducibly upregulated under stroke conditions and consequently drives pathological elevation of GNAI2, targeting IRF7 may provide a more selective and therapeutically viable strategy to restore microglial lipophagy and mitigate impaired LD turnover, with a potentially superior safety profile compared to direct GNAI2 inhibition. In addition, reanalysis of an additional tMCAO/R scRNA-seq dataset produced findings consistent with those from the pdMCAO dataset, further suggesting that the *Irf7*–*Gnai2*–LD axis may represent a common microglial response across different ischemic stroke models.

To identify a pharmacological approach that suppresses microglial IRF7, we targeted its upstream STING signaling. Activation of STING results in the recruitment and activation of TBK1, which phosphorylates IRF7 and drives its nuclear translocation to regulate downstream gene transcription [18]. Notably, STING is persistently and selectively expressed in microglia, but not in astrocytes or neurons, following ischemic stroke [38, 75, 76], which aligns with our findings. Extensive evidence has shown that STING inhibitors exert neuroprotective effects during the acute phase poststroke, primarily by modulating microglial polarization, pyroptosis and synaptic engulfment [38, 75, 76]. In the present study, we further expanded the therapeutic window of the STING inhibitor C-176 to the subacute phase of stroke: delayed administration, even when initiated at day 3 or day 7 poststroke, still markedly improved functional outcomes and synaptic plasticity. Moreover, C-176 alleviated poststroke LD accumulation and lipophagy impairment, which was accompanied by decreased IRF7, p-IRF7, TBK1, p-TBK1 and GNAI2 expression and increased PC levels. More importantly, the beneficial effects of STING signaling inhibition could not be further enhanced in *Irf7* cKO mice. These results suggest that the suppression of IRF7 and GNAI2 constitutes a novel mechanism underlying the therapeutic effects of STING inhibition. Additionally, the microglia-specific expression of STING, together with the efficacy of delayed treatment, highlights the translational potential of STING inhibitors for rehabilitation after stroke.

Despite unveiling the crucial role of the IRF7 in microglial lipophagy impairment during the subacute phase of ischemic stroke, several limitations and future directions remain. First, although the majority of Iba-1⁺ cells in the peri-infarct region during the subacute phase were microglia, the use of the constitutive *Cx3cr1-Cre* system does not completely exclude the potential contribution of infiltrating macrophages after stroke. Second, although PC levels were measured at the tissue level, microglia-specific lipid alterations following ischemic stroke, particularly those regulated by the IRF7/GNAI2, were not determined *in vivo/in situ*. Therefore, the cell-type resolution of current observations remains limited, and future studies are warranted to validate these findings with cell-type-resolved lipidomic approaches. Third, in addition to impaired lipophagy, LD accumulation may also be influenced by increased lipid uptake, enhanced LD biogenesis, and lipolysis. These possibilities need further investigation in future studies. Finally, only male mice were included in this study, and potential sex-dependent differences therefore remain to be investigated.

## Conclusion

**Fig. 10:**
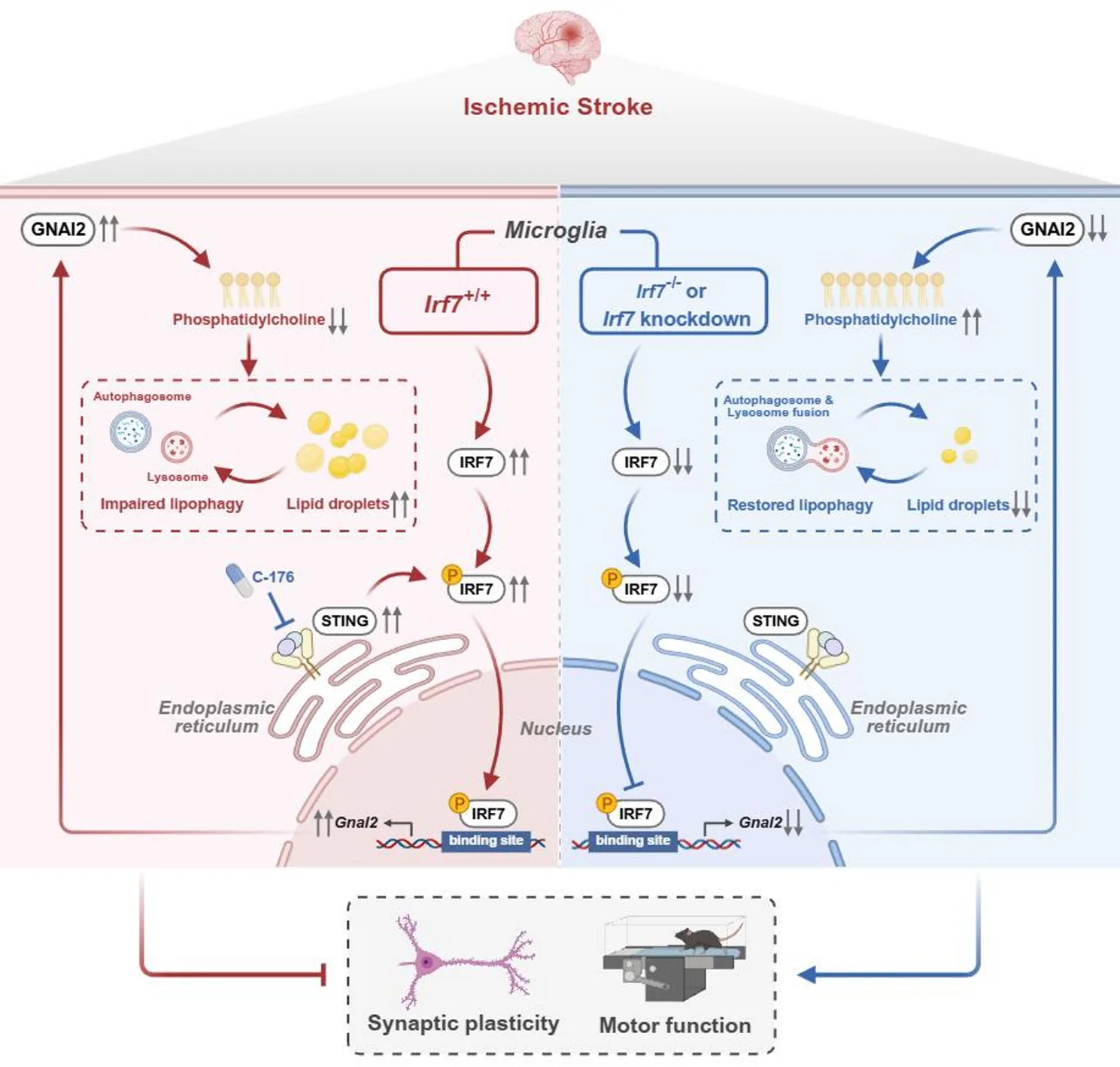
A schematic diagram illustrating the role and mechanism of microglial IRF7 following ischemic stroke. Created with BioRender.com

In conclusion, sustained impairment of microglial lipophagy is a hallmark of neuroinflammation after ischemic stroke from the subacute stage to the chronic stage. Upregulated IRF7 drives this process by transcriptionally activating *Gnai2* and thereby reducing PC levels (Fig. 10). Targeting the STING-IRF7 axis in microglia effectively attenuated lipophagy impairment and LD accumulation, and improved synaptic plasticity and functional recovery. Thus, this study not only uncovers a novel mechanism through which immune signaling disrupts lipid metabolism in microglia, but also provides a promising therapeutic strategy for neurological recovery after stroke.

## Supplementary Information

Additional file 1. Fig. S1 Generation and genotyping of conditional knockout mice. Fig. S2 Spatiotemporal distribution of microglia and macrophages in the peri-infarct region following ischemic stroke. Fig. S3 TrC reduces LDs and promotes motor function recovery following ischemic stroke. Fig. S4 Time-course changes in autophagy levels following ischemic stroke. Fig. S5 QC of scRNA-seq data from sham and pdMCAO groups. Fig. S6 *Irf7*^*high*^ microglia subcluster engages autophagy and lipid metabolism pathways following ischemic stroke. Fig. S7 Microglial *Irf7* deficiency reduces gliosis and infarct volume following ischemic stroke. Fig. S8 Microglial *Irf7* deficiency exerts beneficial effects following ischemic stroke in 15-month-old mice. Fig. S9 Time- and dose-dependent changes in LDs, autophagy and IRF7 in LPS-stimulated BV2 cells. Fig. S10 Validation of *Irf7* knockdown and *Gnai2* knockdown efficiency in BV2 cells. Fig. S11 Rapamycin enhances autophagic flux to reduce LD accumulation in LPS-stimulated BV2 cells. Fig. S12 Correlation between *Irf7* and *Gnai2*. Fig. S13 Exogenous CDP-choline supplementation alleviates LD accumulation and promotes motor function recovery following ischemic stroke. Fig. S14 Spatiotemporal changes of STING signaling expression at different time points after PTI. Fig. S15 Effects of C-176 on infarct volume, downstream STING signaling proteins and gliosis. Fig. S16 The STING inhibitor C-176 reduces LD accumulation in LPS-stimulated primary microglia and BV2 cells.

Additional file 2. Table S1. A list of primers sequences for mouse genotyping. Table S2. A list of primers sequences for RT–qPCR. Table S3. A list of primary and secondary antibodies for western blot. Table S4. A list of primary and secondary antibodies for immunofluorescence. Table S5. A list of siRNA target sequences for gene knockdown. Table S6. A list of primers sequences for ChIP–qPCR.

Additional file 3. Original western blot images.

Additional file 4. DEGs list for *Irf7*^*high*^ vs. *Irf7*^*low*^ microglia in the MG1 subcluster (GSE261494).

Additional file 5. DEGs list for *Irf7*^*high*^ vs. *Irf7*^*low*^ microglia in the MG1 subcluster (GSE225948).

## Data Availability

The datasets analyzed in this study are publicly available. The single-cell RNA sequencing data were obtained from the Gene Expression Omnibus (GEO) database under accession number GSE261494, GSE225948. Other data supporting the findings of this study are available from the corresponding author upon reasonable request.

## Abbreviations

LD: Lipid droplet
IRF7: Interferon regulatory factor 7
PTI: Photothrombotic ischemia
*Irf7* cKO: Microglial *Irf7* conditional knockout
PC: Phosphatidylcholine
STING: Stimulator of interferon genes
GNAI2: G protein subunit alpha i2
CNS: Central nervous system
LDAM: Lipid droplet-accumulating microglia
TrC: Triacsin C
OGD: Oxygen-glucose deprivation
scRNA-seq: Single-cell RNA sequencing
WT: Wild-type
LF: Left forelimb
LH: Left hindlimb
PLIN2: Perilipin 2
Iba-1: Ionized calcium binding adaptor molecule-1
GFAP: Glial fibrillary acidic protein
RT-qPCR: Real-time quantitative polymerase chain reaction
GAPDH: Glyceraldehyde-3-phosphate dehydrogenase
TEM: Transmission electron microscope
LPS: Lipopolysaccharide
NC: Negative control
ChIP: Chromatin Immunoprecipitation
pdMCAO: Permanent distal middle cerebral artery occlusion
QC: Quality control
UMAP: Uniform manifold approximation and projection
GO: Gene Ontology
KEGG: Kyoto Encyclopedia of Genes and Genomes
