# Supplementary material for: Microglial IRF7-induced lipophagy impairment aggravates lipid droplet overload and impedes neurological recovery after ischemic stroke
Source: J Neuroinflammation. 2026 Jun 17;23:287. doi: 10.1186/s12974-026-03902-3 (PMC13508275; doi:10.1186/s12974-026-03902-3)

Original western blot images

Boxed regions indicate the areas cropped for presentation in the main figures.

Fig. 5G

Beclin 1 (60kDa)

Lane information (From left to right): NC-Con, NC-LPS, Irf7-siRNA-Con, Irf7-siRNA-LPS; Each biological replicate set was separated by a molecular weight marker lane.

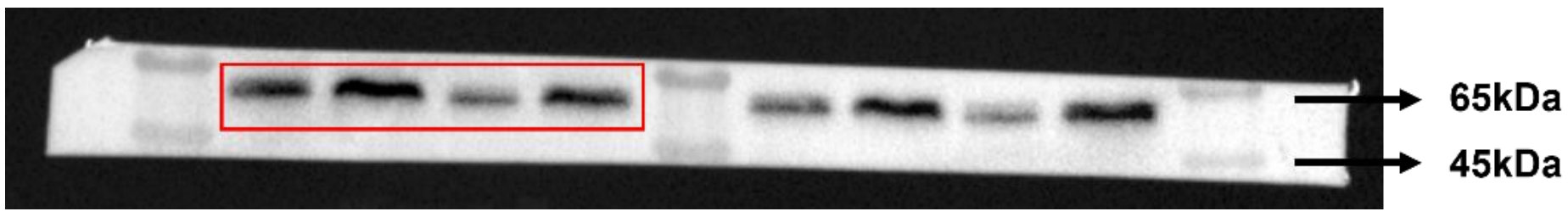

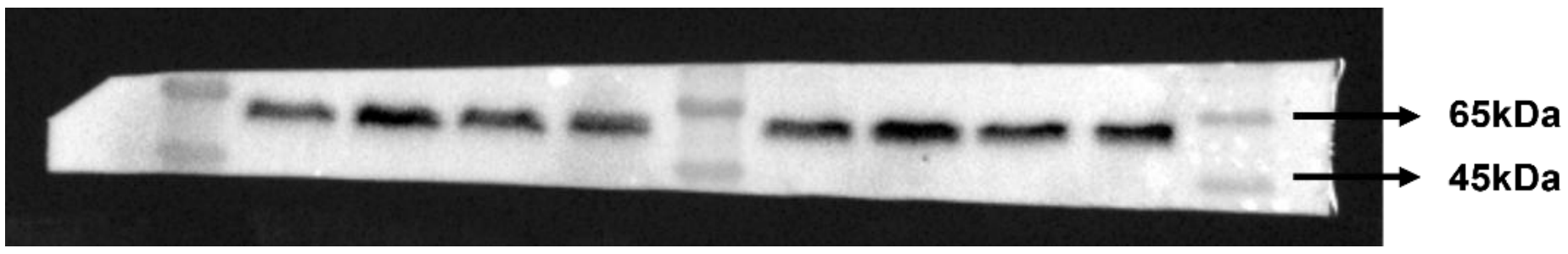

LC3 II/I (12, 10kDa)

Lane information (From left to right): NC-Con, NC-LPS, Irf7-siRNA-Con, Irf7-siRNA-LPS; Each biological replicate set was separated by a molecular weight marker lane.

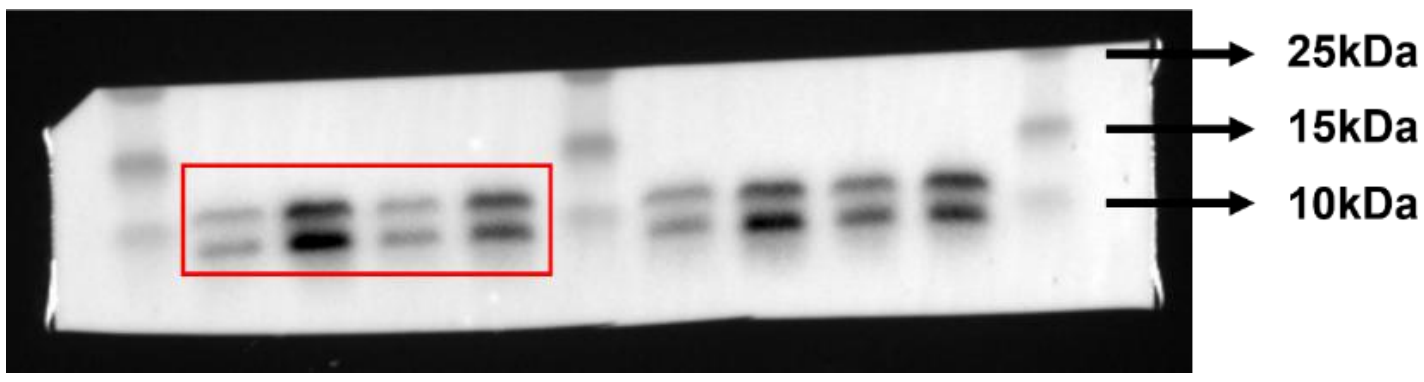

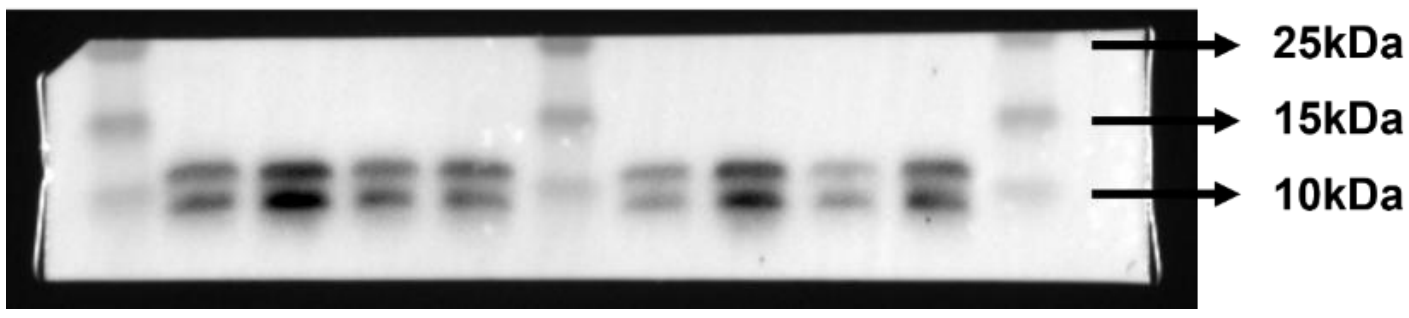

p62 (62kDa)

Lane information (From left to right): NC-Con, NC-LPS, Irf7-siRNA-Con, Irf7-siRNA-LPS; Each biological replicate set was separated by a molecular weight marker lane.

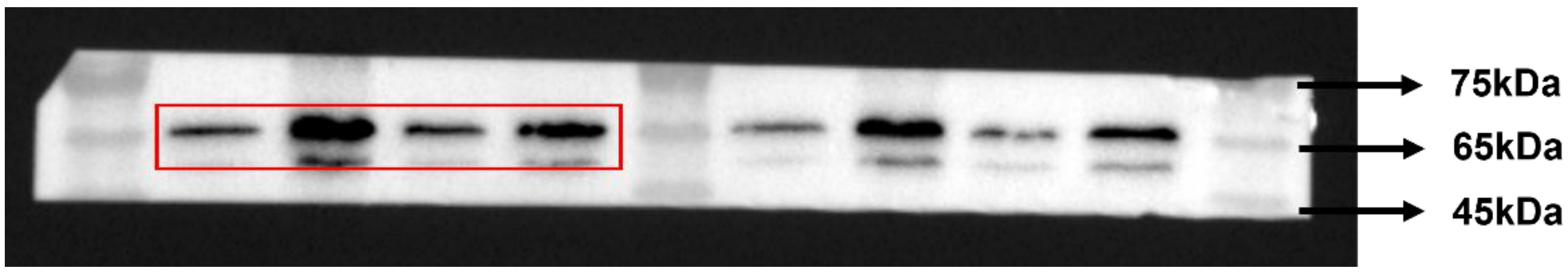

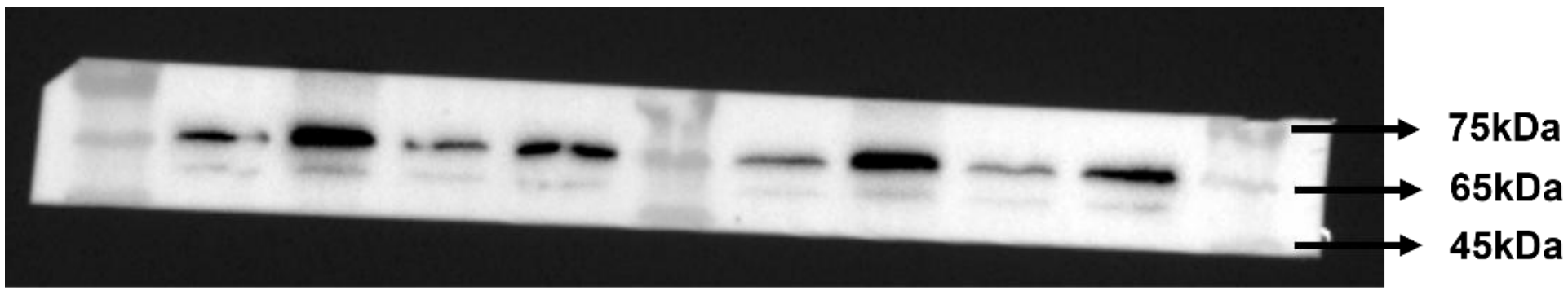

GAPDH (37kDa)

Lane information (From left to right): NC-Con, NC-LPS, Irf7-siRNA-Con, Irf7-siRNA-LPS; Each biological replicate set was separated by a molecular weight marker lane.

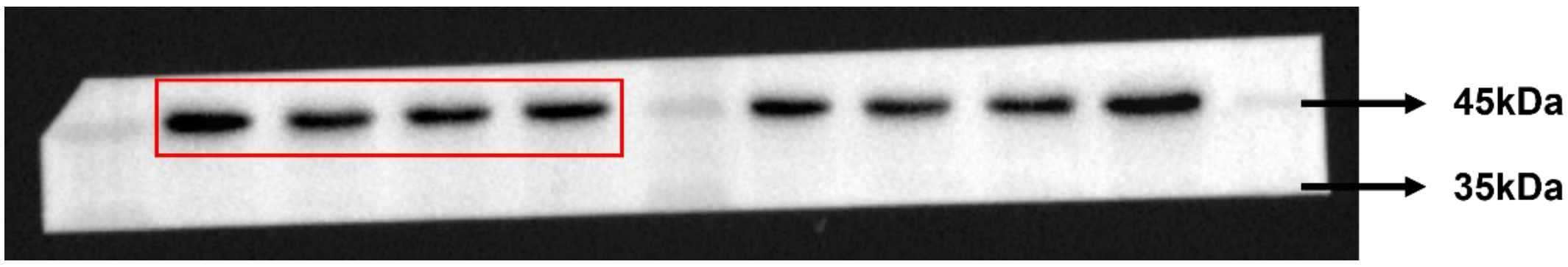

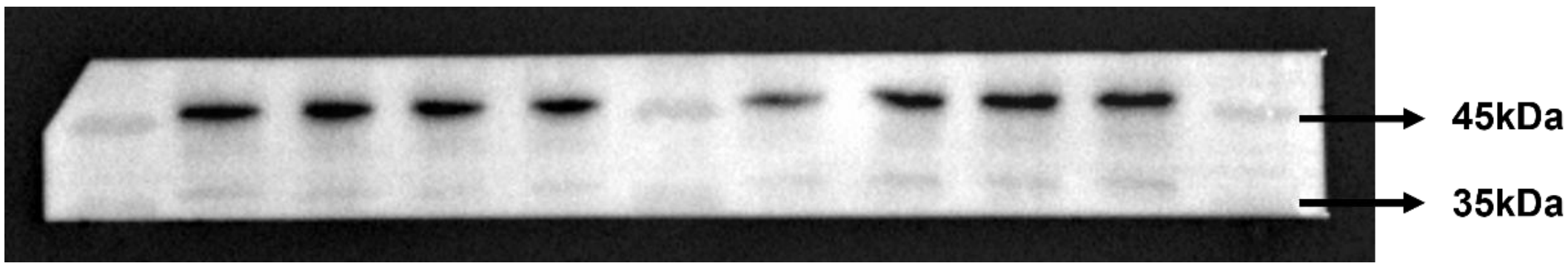

Fig. 6D

GNAI2 (40kDa)

Lane information (From left to right): NC-Con, NC-LPS, Irf7-siRNA-Con, Irf7-siRNA-LPS; Each biological replicate set was separated by a molecular weight marker lane.

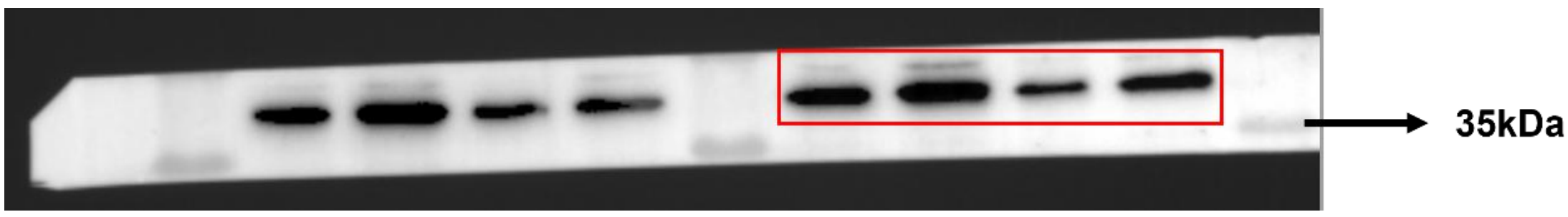

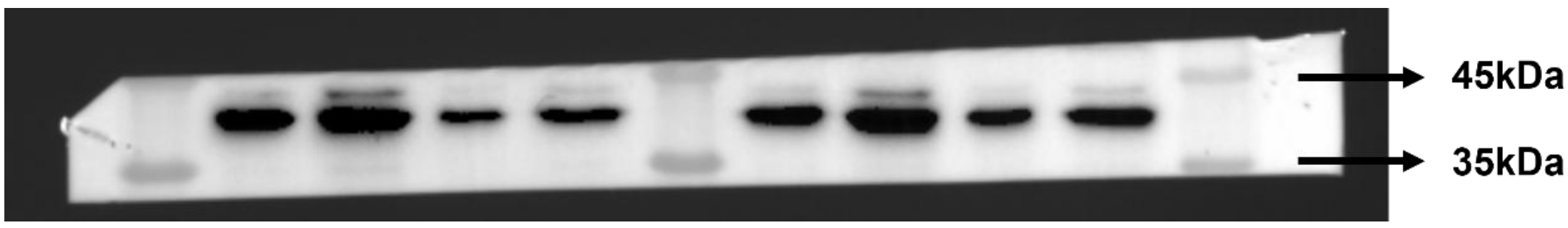

GAPDH (37kDa)

Lane information (From left to right): NC-Con, NC-LPS, Irf7-siRNA-Con, Irf7-siRNA-LPS; Each biological replicate set was separated by a molecular weight

marker lane.

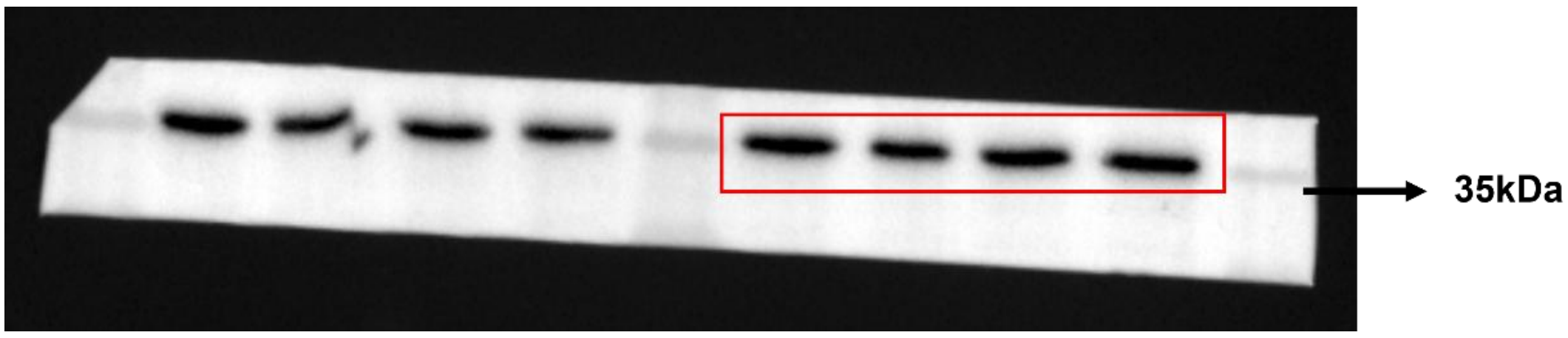

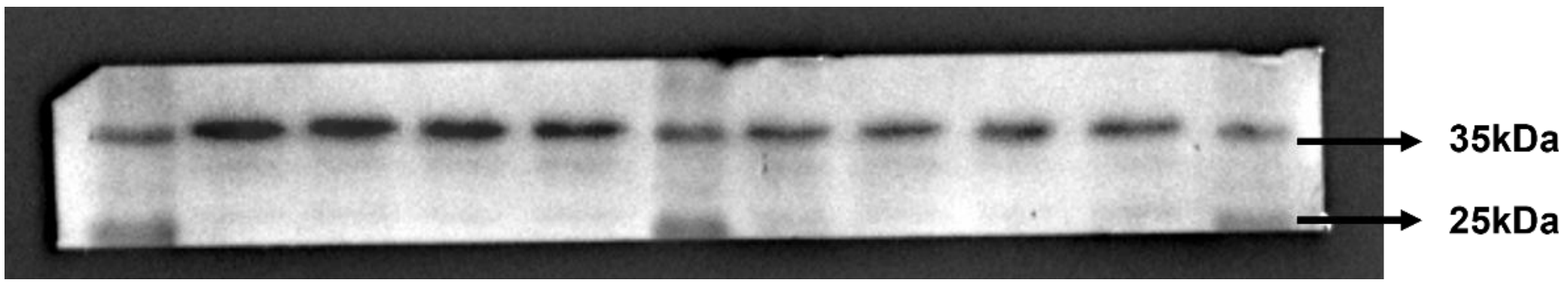

Fig. 8D

STING (42kDa)

Lane information (From left to right): Sham, PTI, PTI+C-176; Each biological replicate set was separated by a molecular weight marker lane.

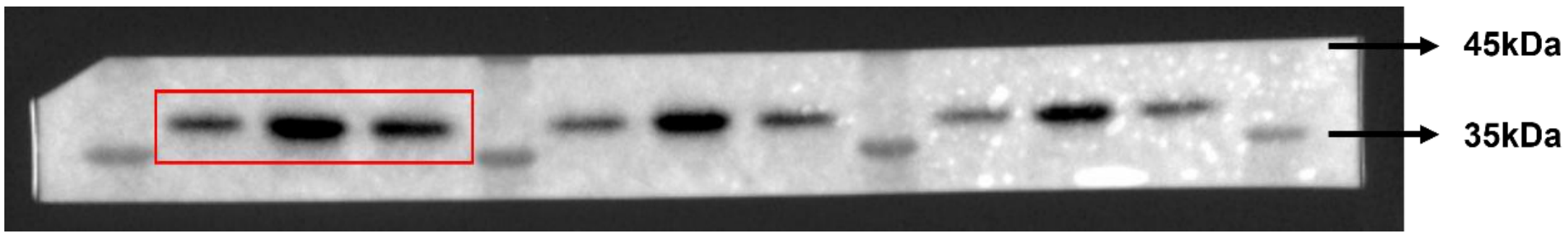

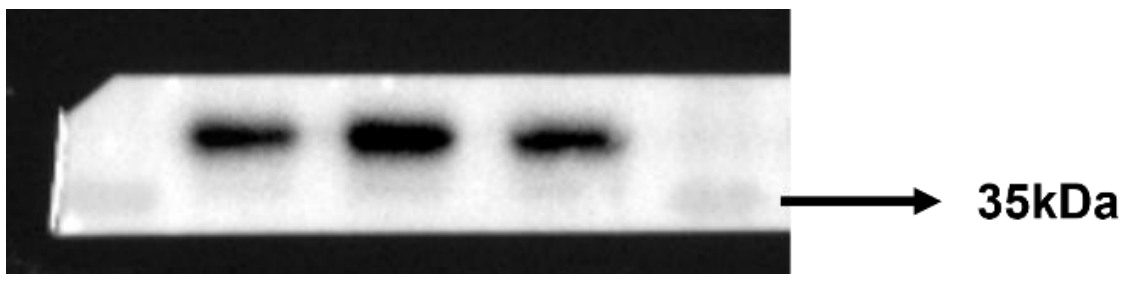

IRF7 (45-60kDa)

Lane information (From left to right): Sham, PTI, PTI+C-176; Each biological replicate set was separated by a molecular weight marker lane.

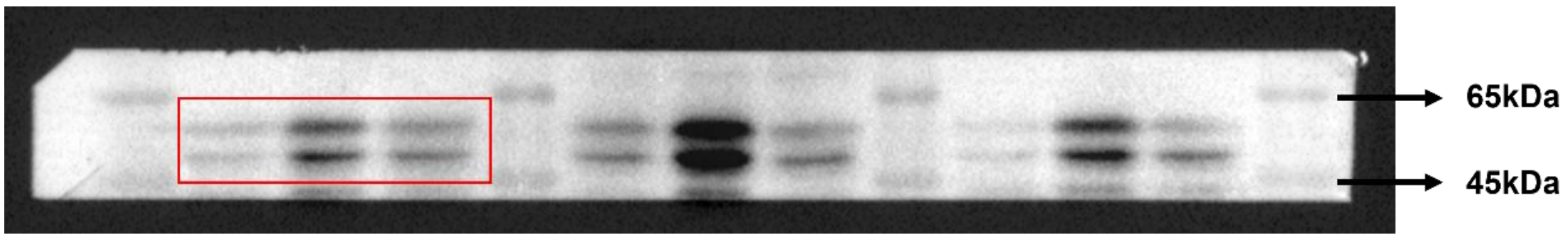

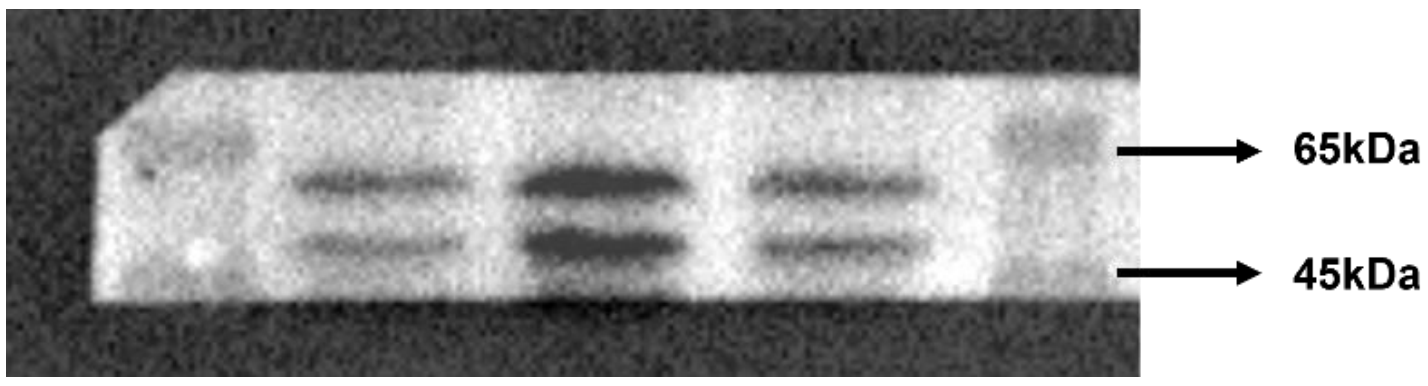

GNAI2 (40kDa)

Lane information (From left to right): Sham, PTI, PTI+C-176; Each biological replicate set was separated by a molecular weight marker lane.

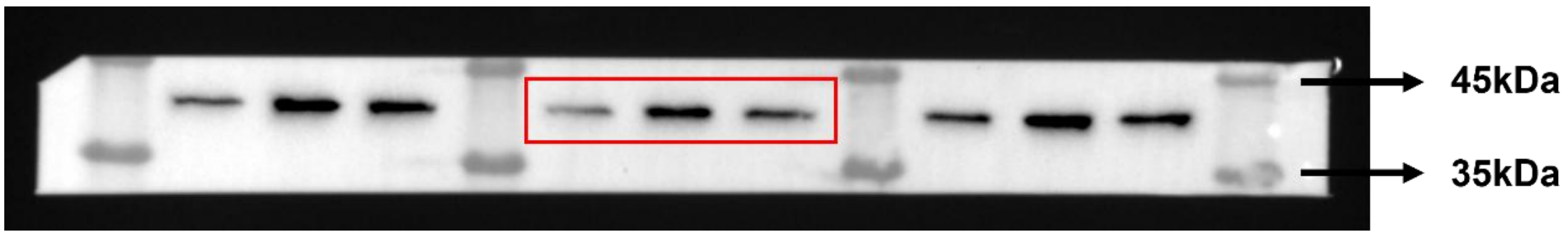

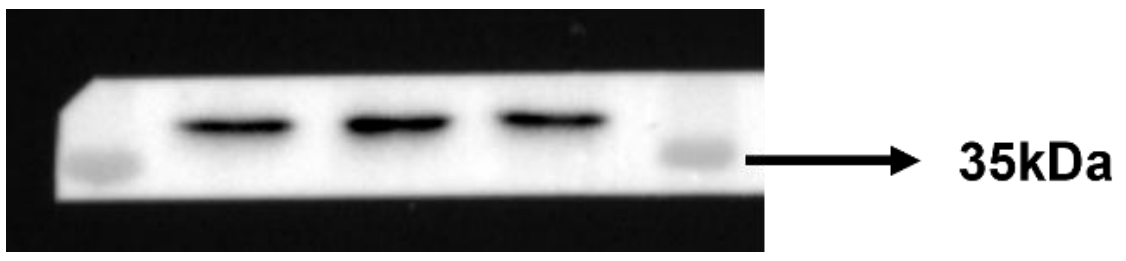

GAPDH (37kDa)

Lane information (From left to right): Sham, PTI, PTI+C-176; Each biological replicate set was separated by a molecular weight marker lane.

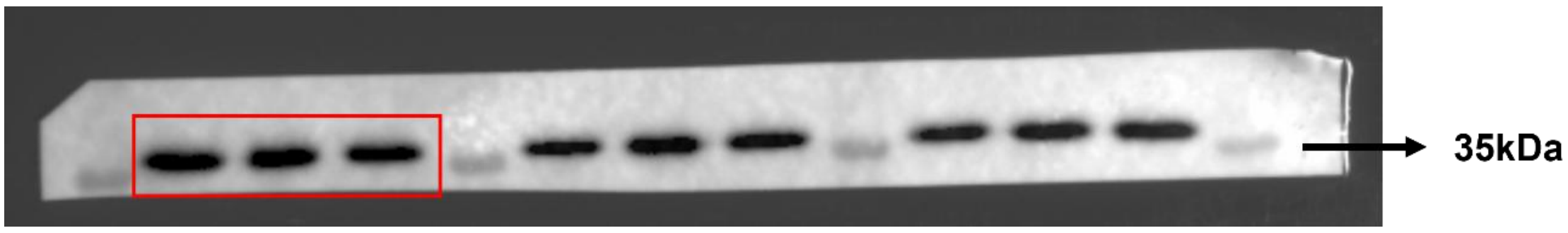

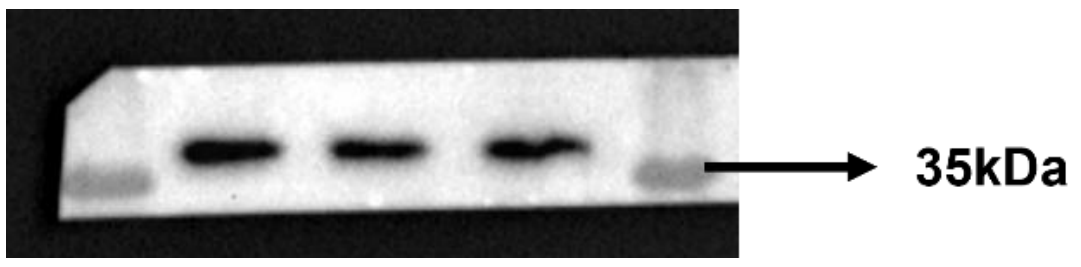

Fig. S4A

Beclin 1 (60kDa)

Lane information (From left to right): Sham, 1d, 3d, 7d,14d, 21d; Each biological replicate set was separated by a molecular weight marker lane.

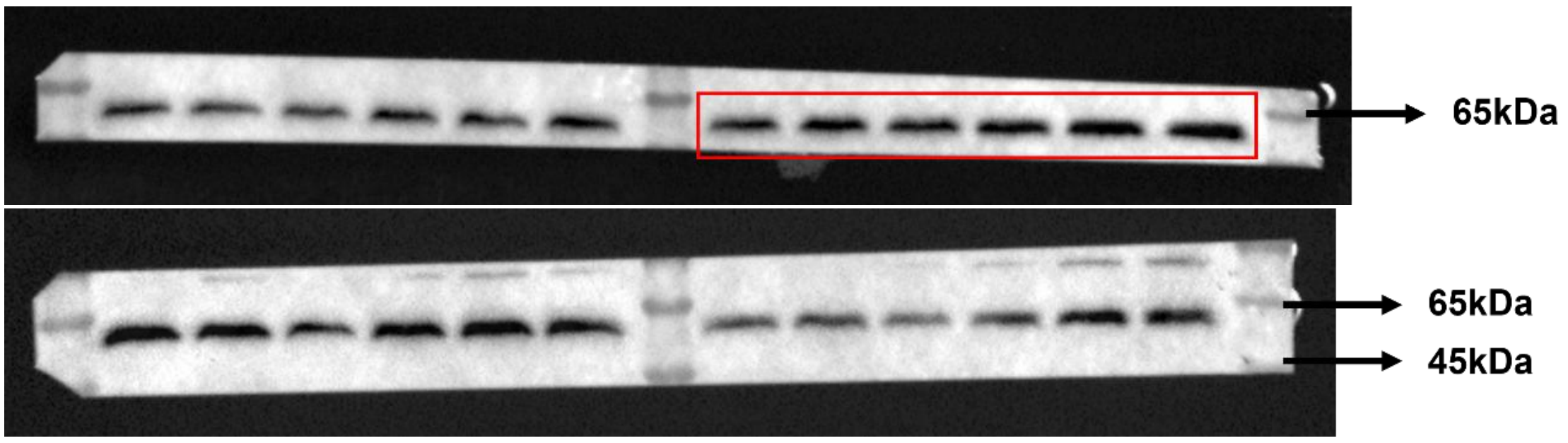

LC3 II/I (12, 10kDa)

Lane information (From left to right): Sham, 1d, 3d, 7d,14d, 21d; Each biological replicate set was separated by a molecular weight marker lane.

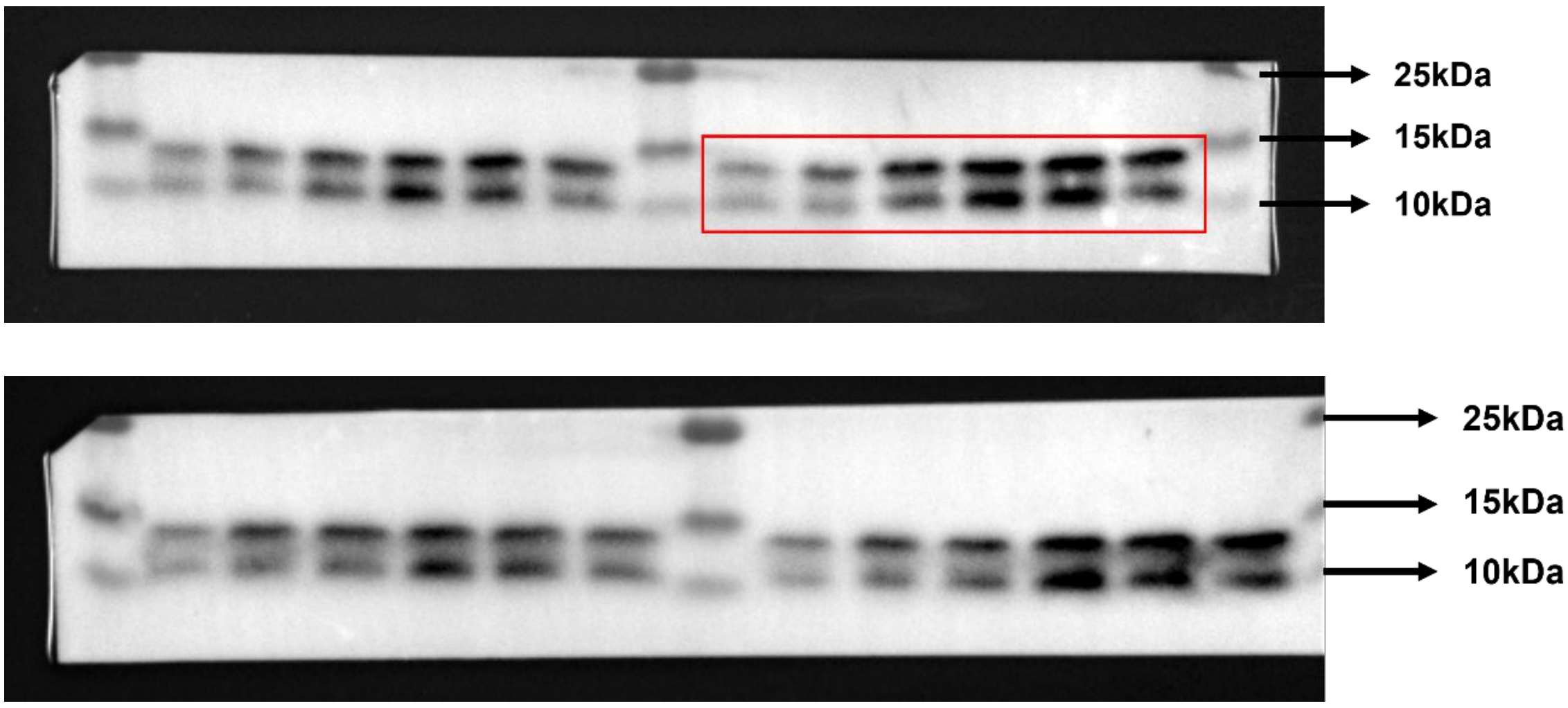

p62 (62kDa)

Lane information (From left to right): Sham, 1d, 3d, 7d,14d, 21d; Each biological replicate set was separated by a molecular weight marker lane.

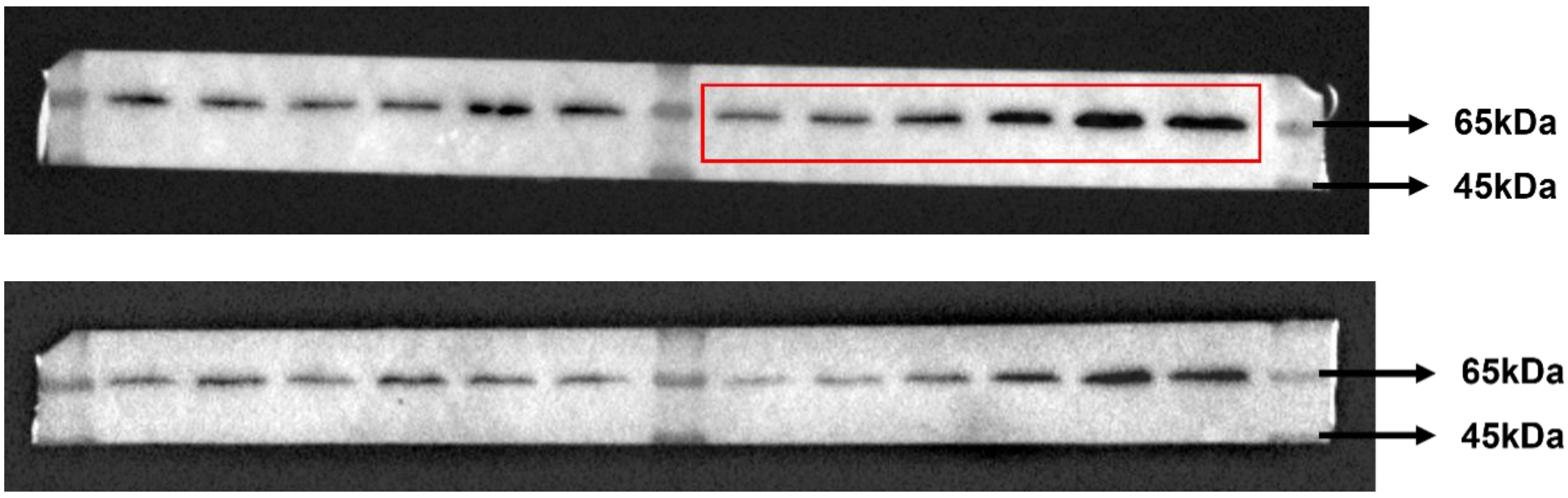

GAPDH (37kDa)

Lane information (From left to right): Sham, 1d, 3d, 7d,14d, 21d; Each biological replicate set was separated by a molecular weight marker lane.

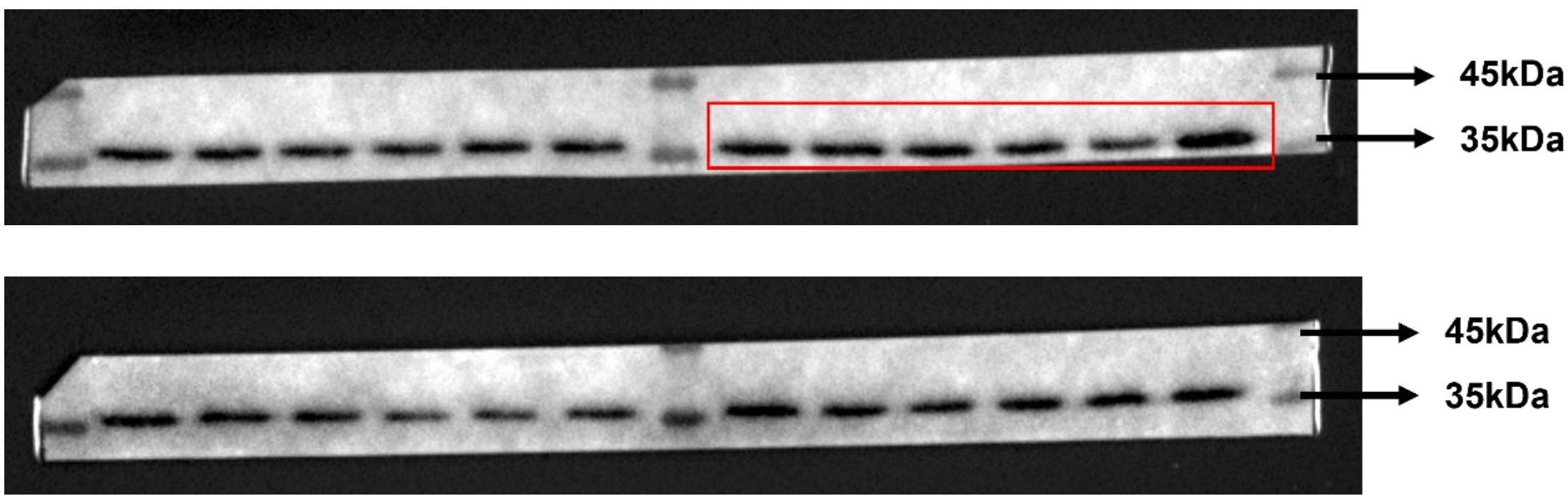

Fig. S9F

IRF7 (45-60kDa)

Lane information (From left to right): Control, LPS 6-10, 6-100, 6-1000, 12-10, 12-100, 12-1000, 24-10, 24-100, 24-1000.

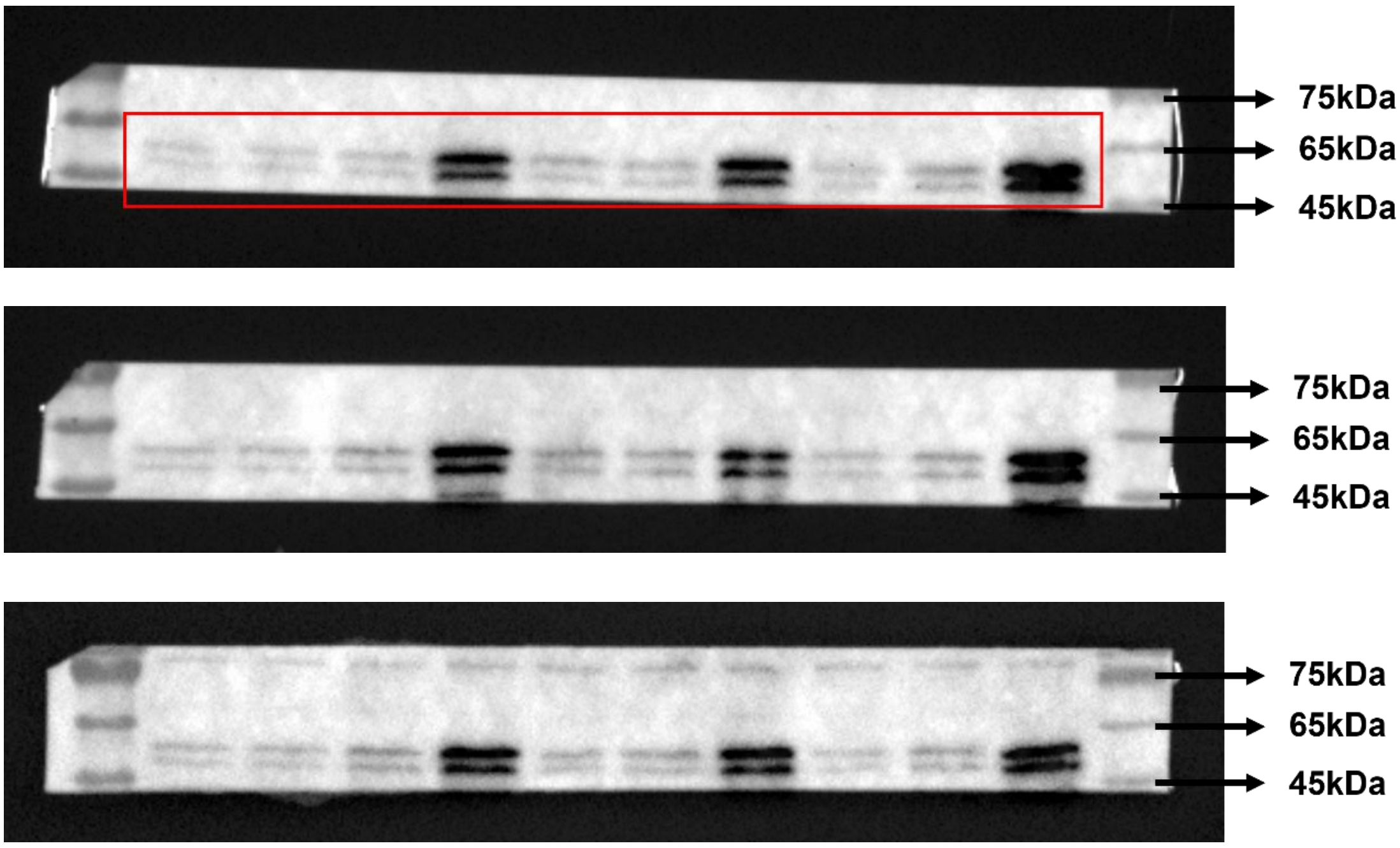

Beclin 1 (60kDa)

Lane information (From left to right): Control, LPS 6-10, 6-100, 6-1000, 12-10, 12-100, 12-1000, 24-10, 24-100, 24-1000.

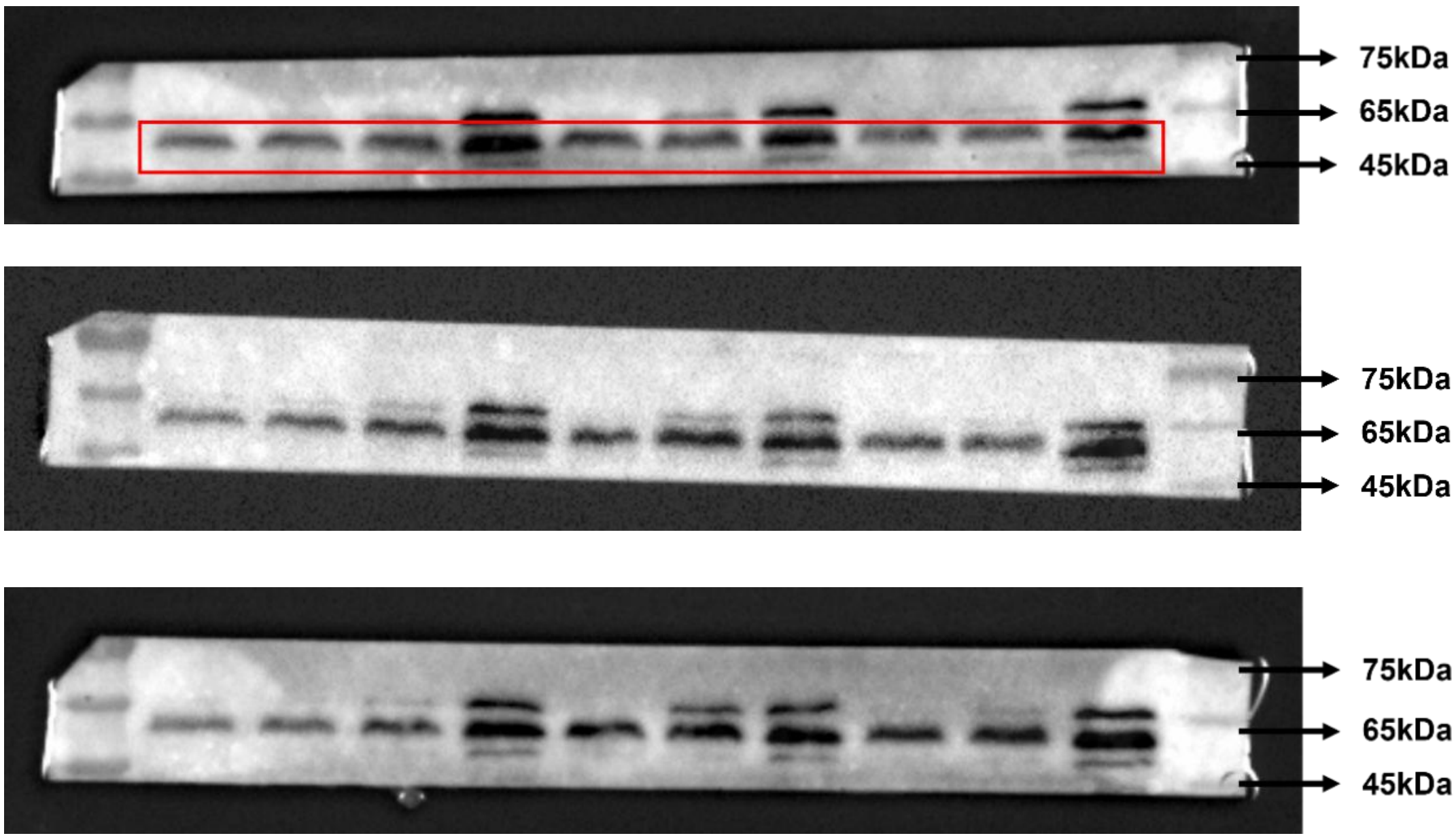

LC3 II/I (12, 10kDa)

Lane information (From left to right): Control, LPS 6-10, 6-100, 6-1000, 12-10, 12-100, 12-1000, 24-10, 24-100, 24-1000.

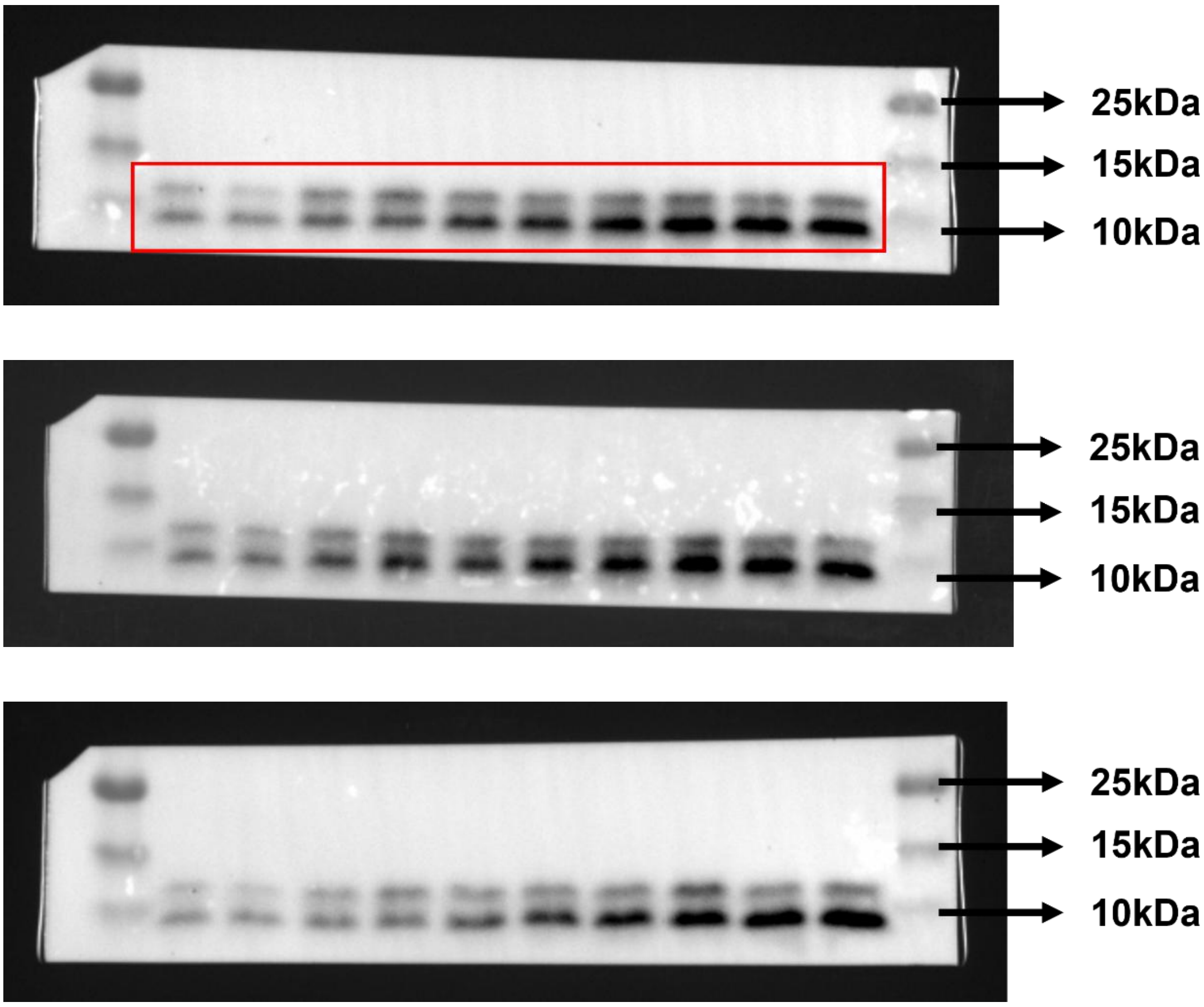

p62 (62kDa)

Lane information (From left to right): Control, LPS 6-10, 6-100, 6-1000, 12-10, 12-100, 12-1000, 24-10, 24-100, 24-1000.

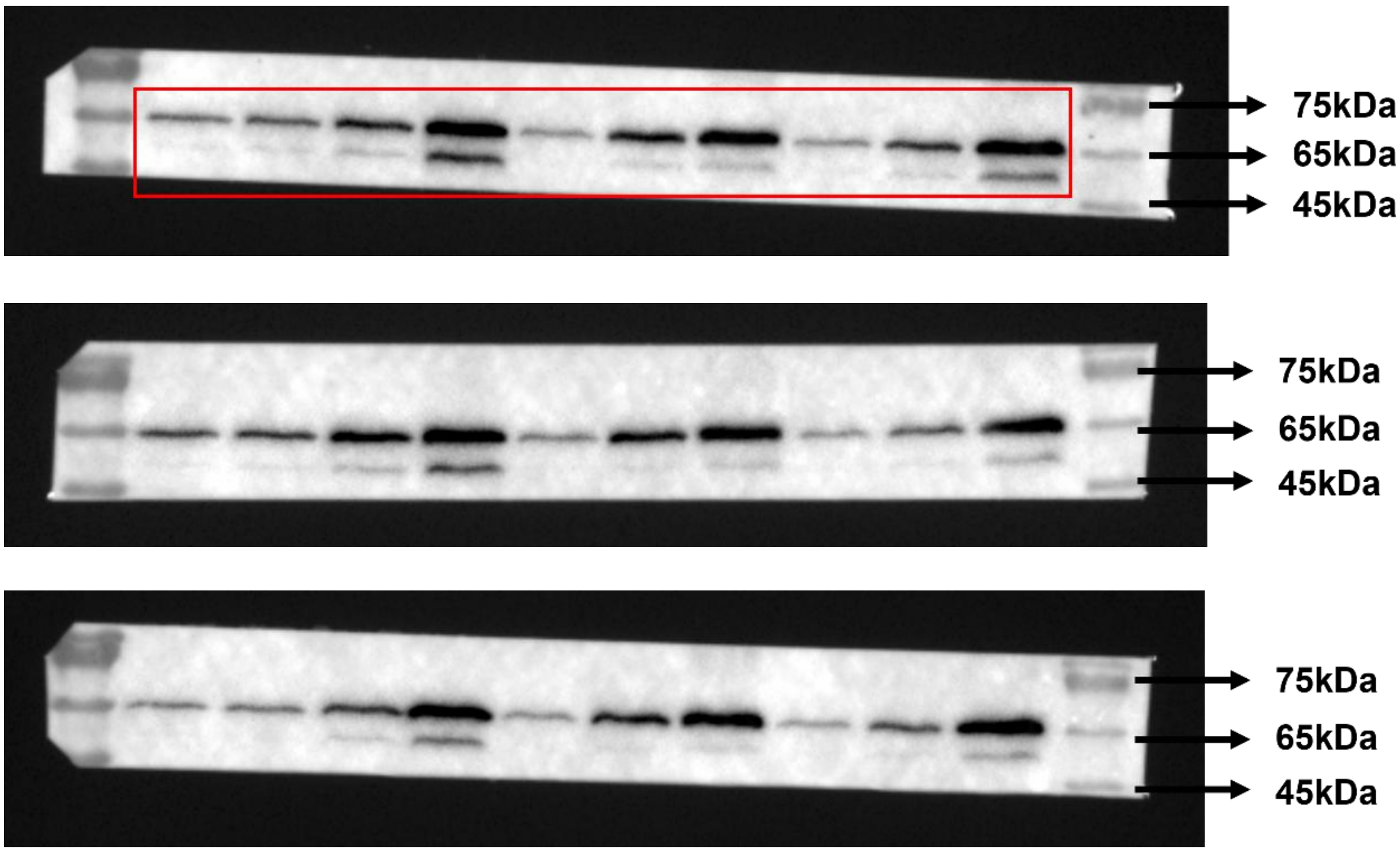

GAPDH (37kDa)

Lane information (From left to right): Control, LPS 6-10, 6-100, 6-1000, 12-10, 12-100, 12-1000, 24-10, 24-100, 24-1000.

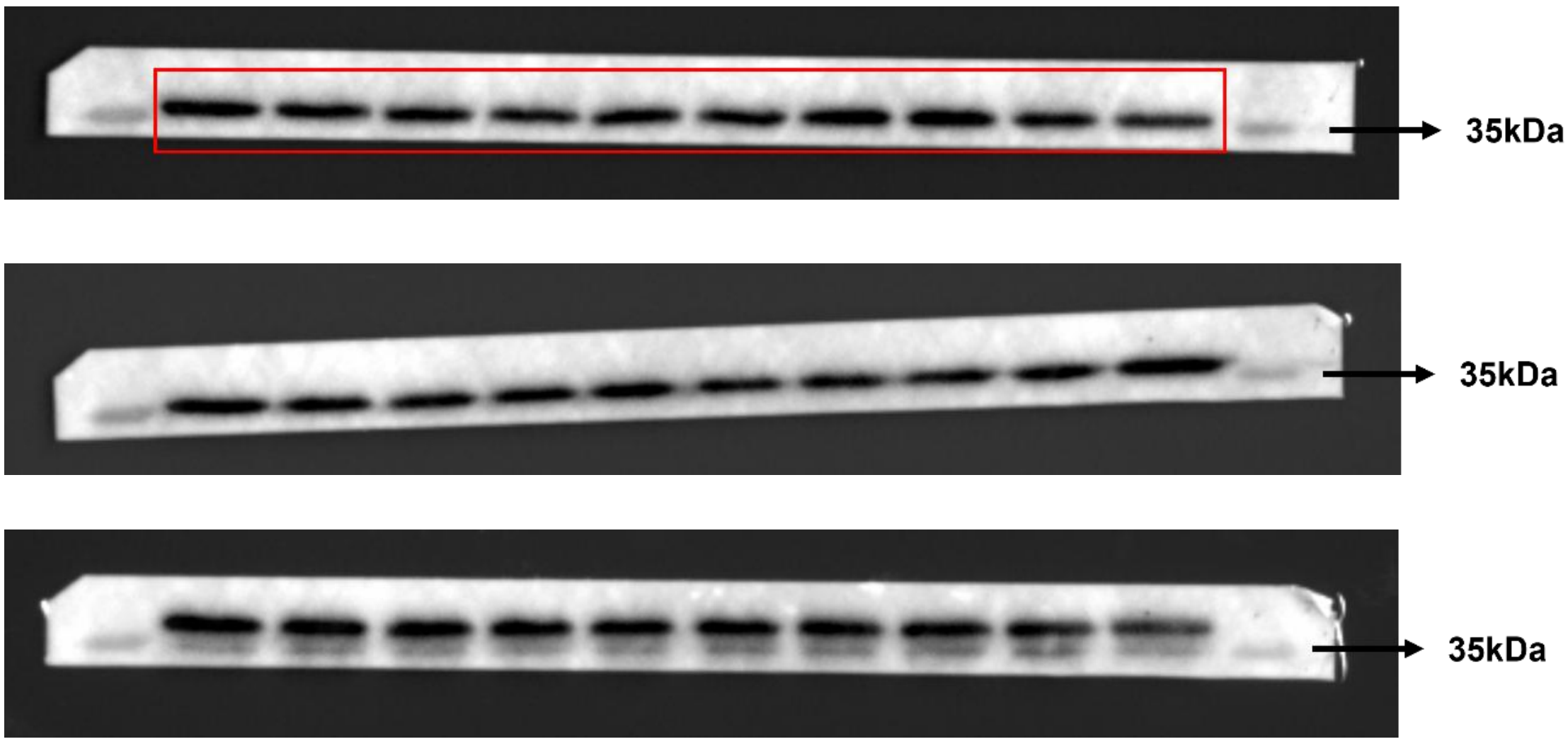

Fig. S10B

IRF7 (45-60kDa)

Lane information (From left to right): NC, si-1, si-2, si-3; Each biological replicate set was separated by a molecular weight marker lane.

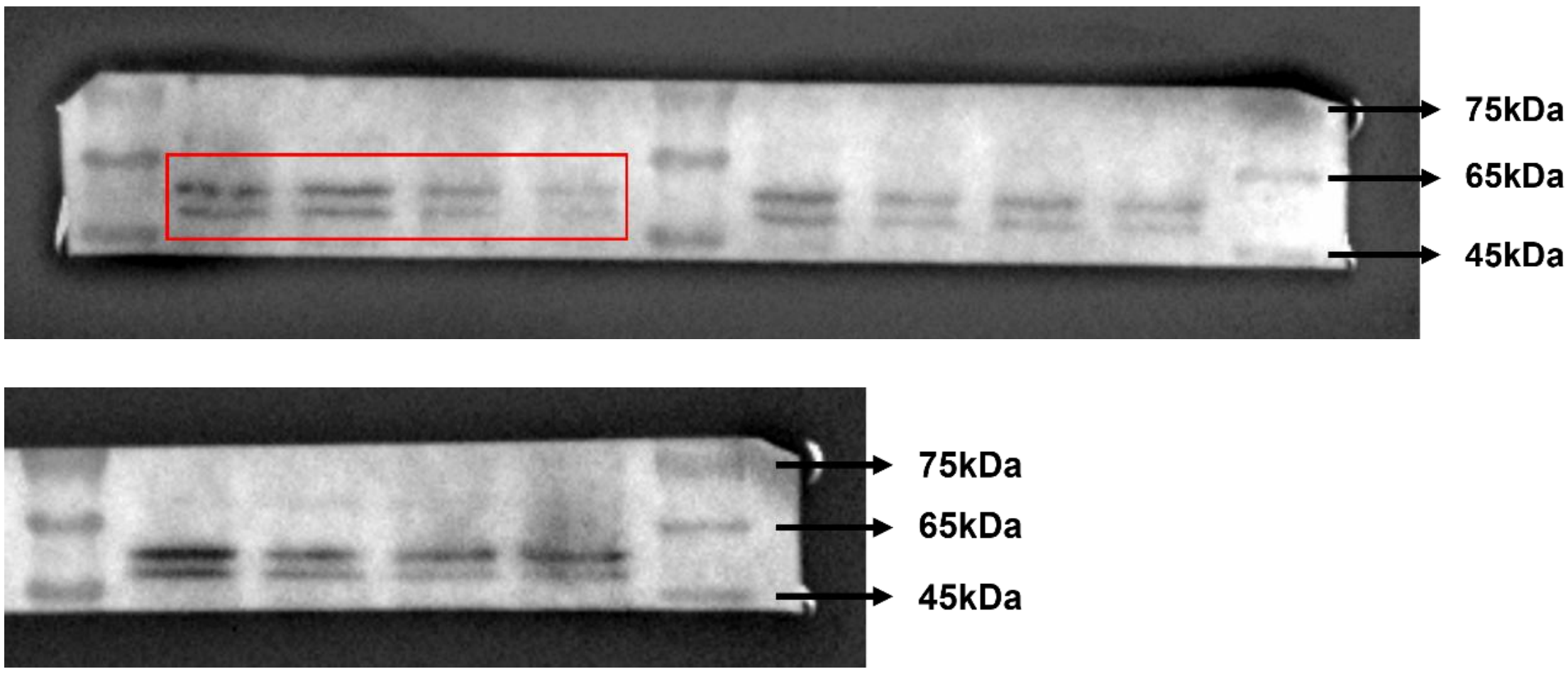

GAPDH (37kDa)

Lane information (From left to right): NC, si-1, si-2, si-3; Each biological replicate set was separated by a molecular weight marker lane.

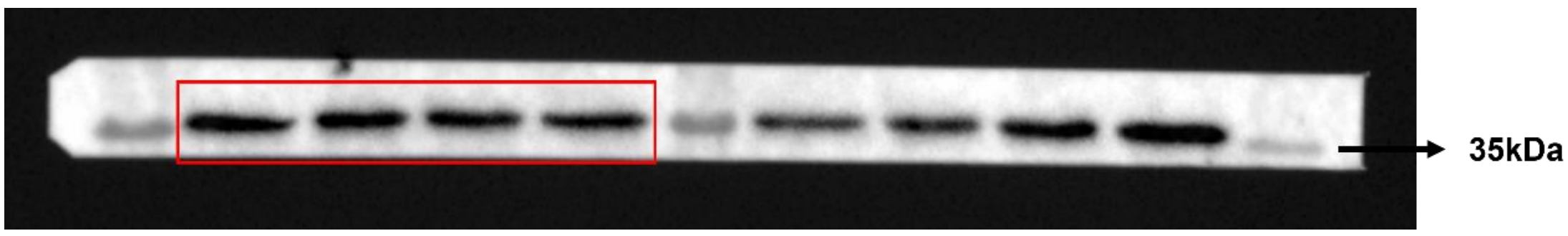

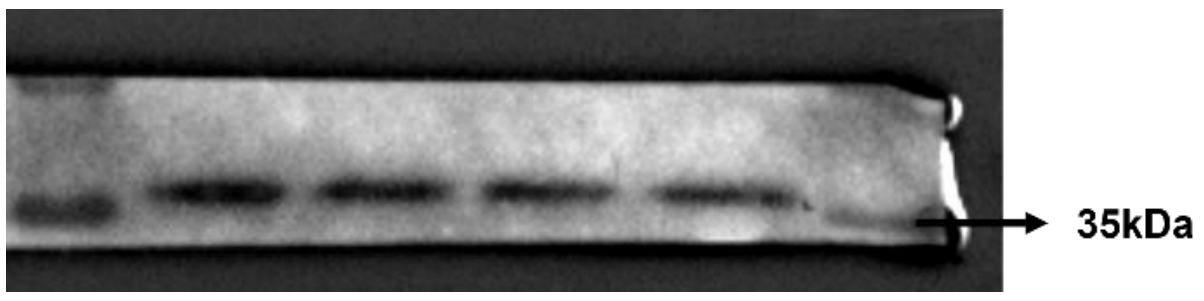

Fig. S10E

GNAI2 (40kDa)

Lane information (From left to right): NC, si-1, si-2, si-3; Each biological replicate set was separated by a molecular weight marker lane.

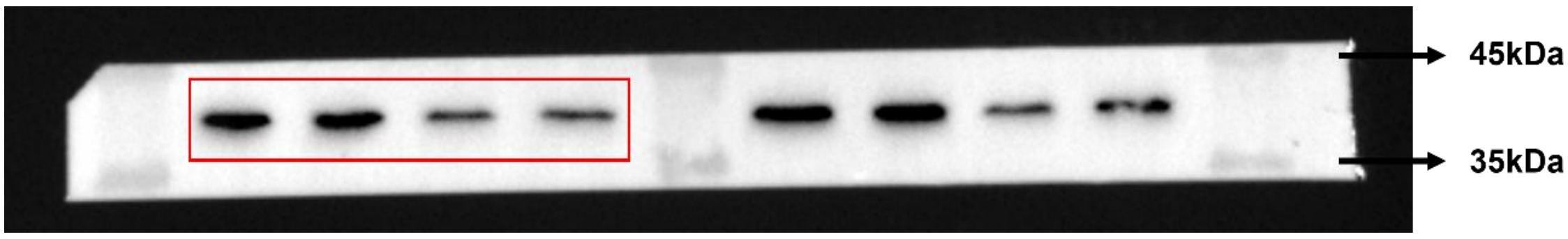

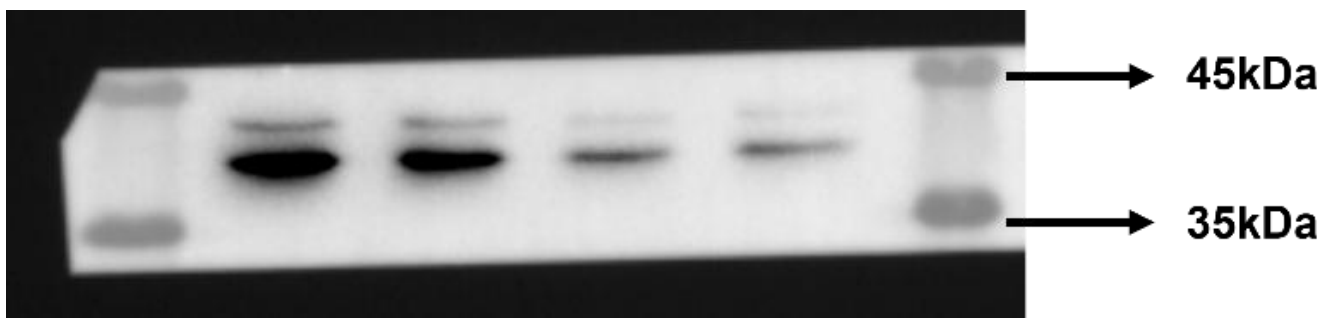

GAPDH (37kDa)

Lane information (From left to right): NC, si-1, si-2, si-3; Each biological replicate set was separated by a molecular weight marker lane.

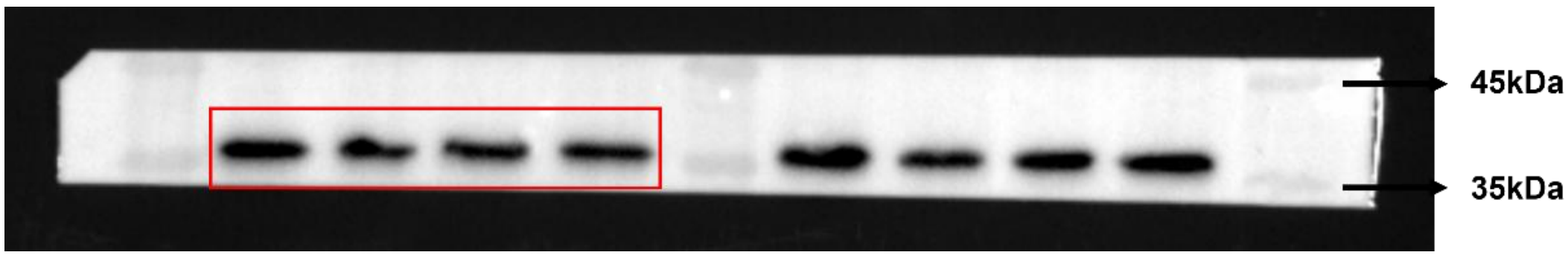

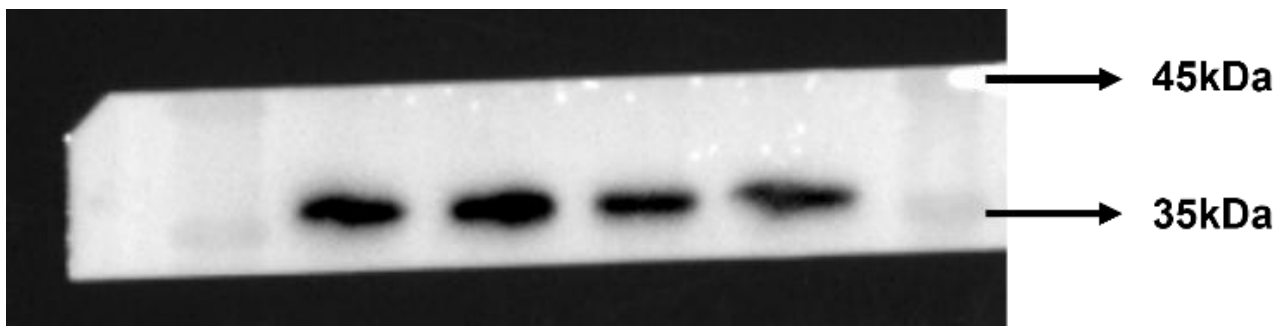

Fig. S14B

STING (42kDa)

Lane information (From left to right): Sham, 1d, 3d, 7d,14d, 21d; Each biological replicate set was separated by a molecular weight marker lane.

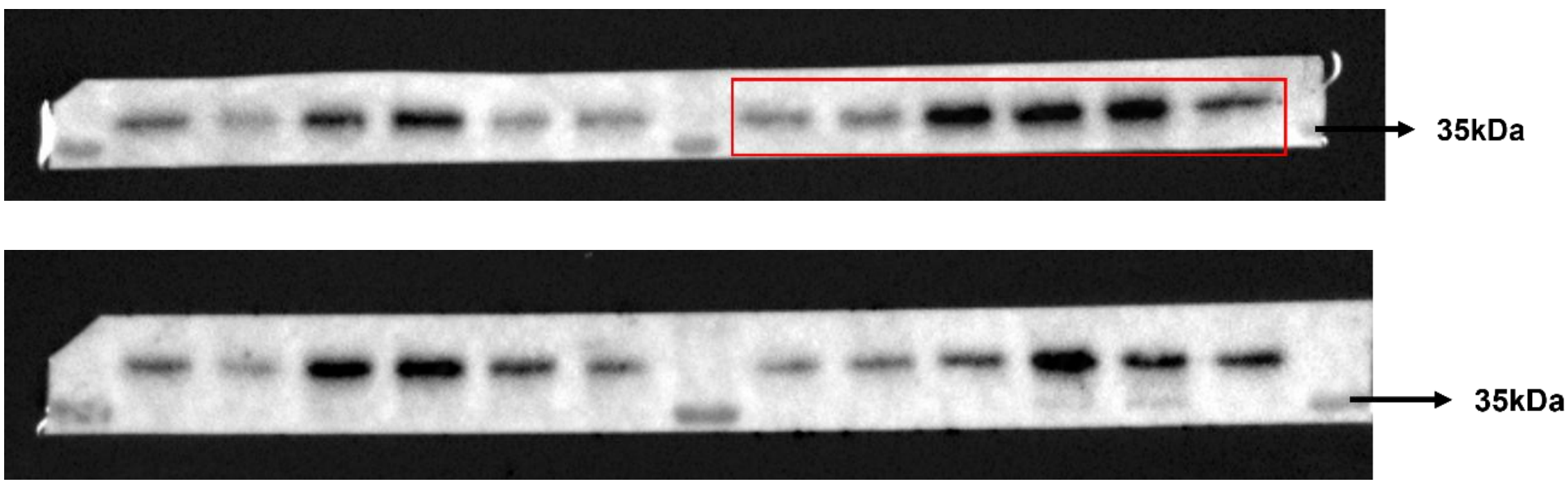

GAPDH (37kDa)

Lane information (From left to right): Sham, 1d, 3d, 7d,14d, 21d; Each biological replicate set was separated by a molecular weight marker lane.

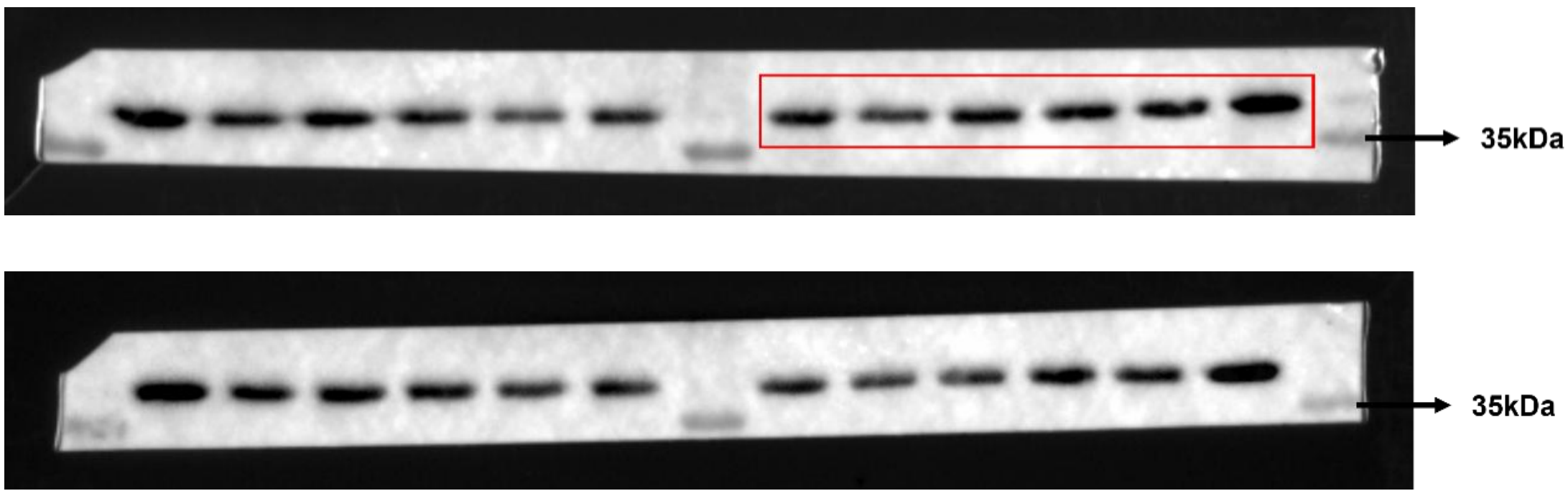

Fig. S15B

p-TBK1 (84kDa)

Lane information (From left to right): Sham, PTI, PTI+C-176; Each biological replicate set was separated by a molecular weight marker lane

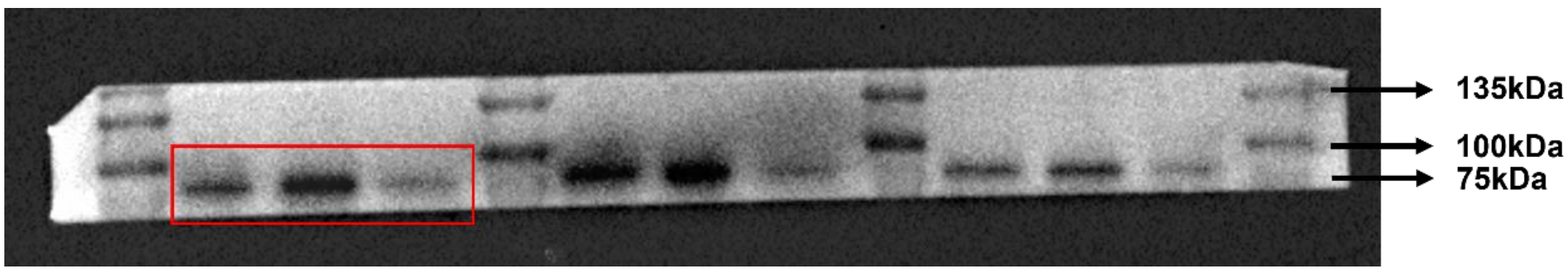

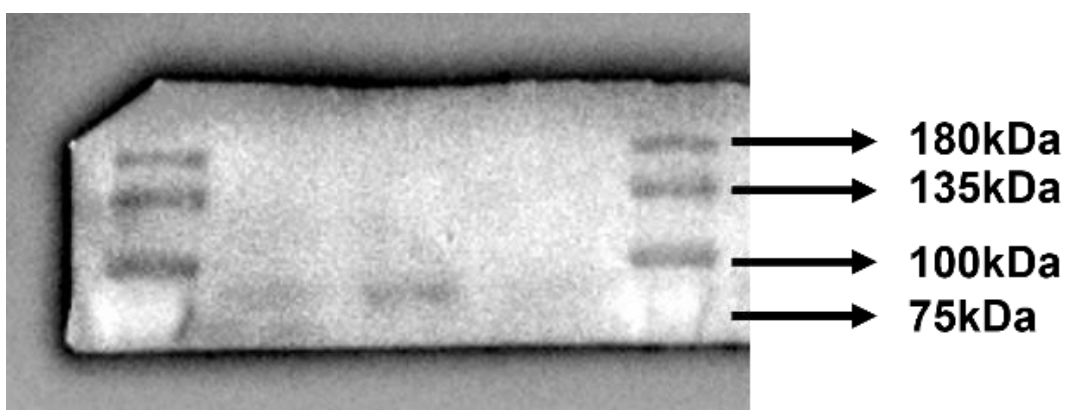

TBK1 (84kDa)

Lane information (From left to right): Sham, PTI, PTI+C-176; Each biological replicate set was separated by a molecular weight marker lane.

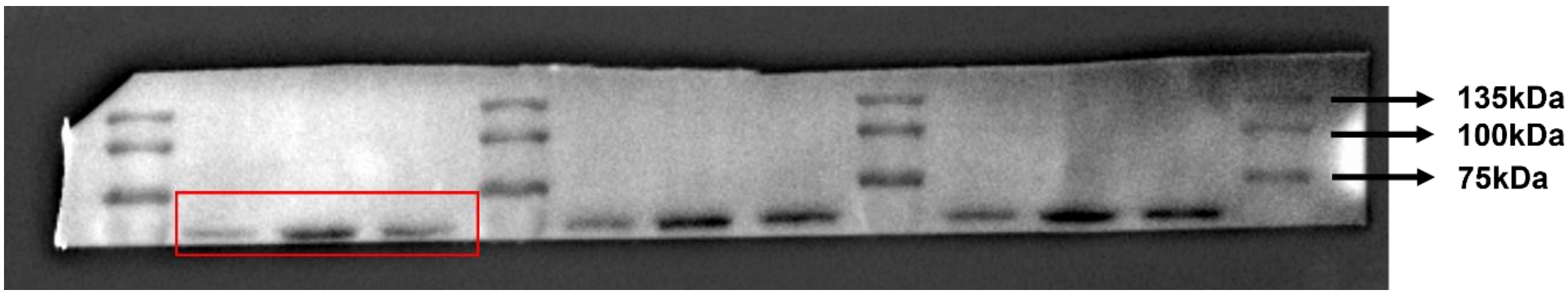

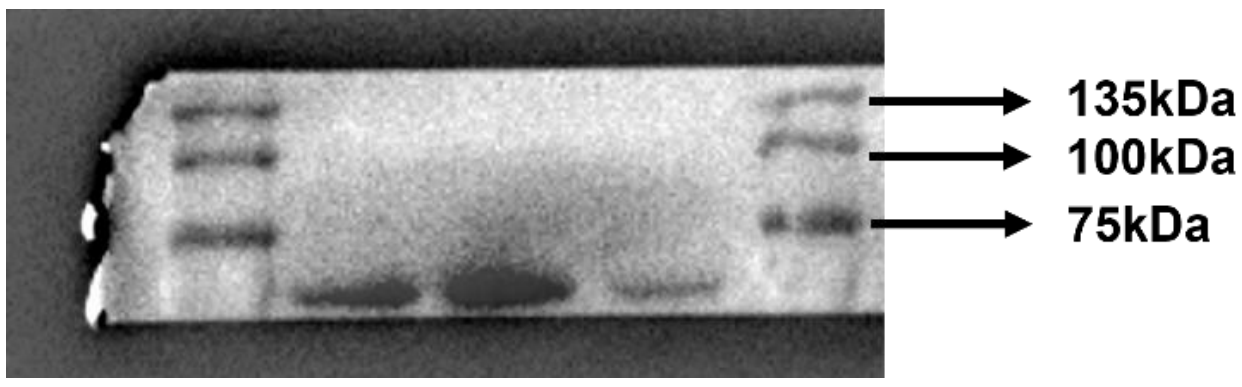

p-IRF7 (55kDa)

Lane information (From left to right): Sham, PTI, PTI+C-176; Each biological replicate set was separated by a molecular weight marker lane.

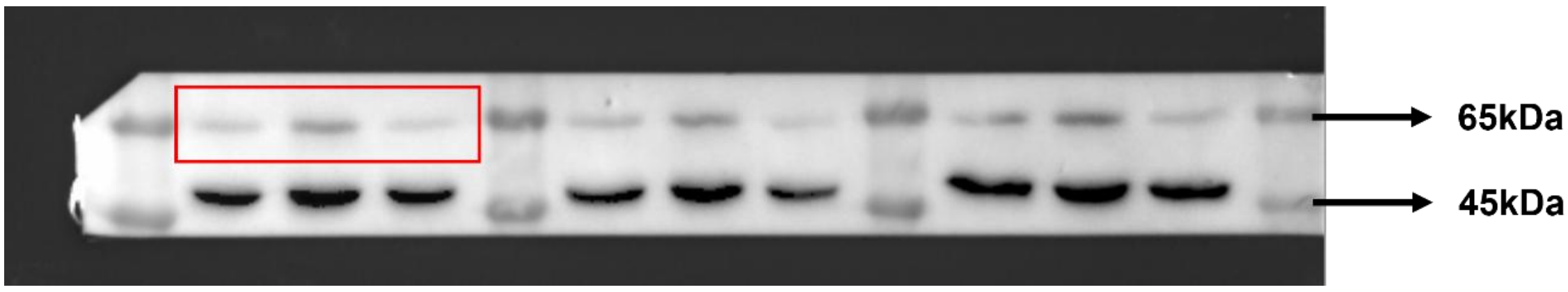

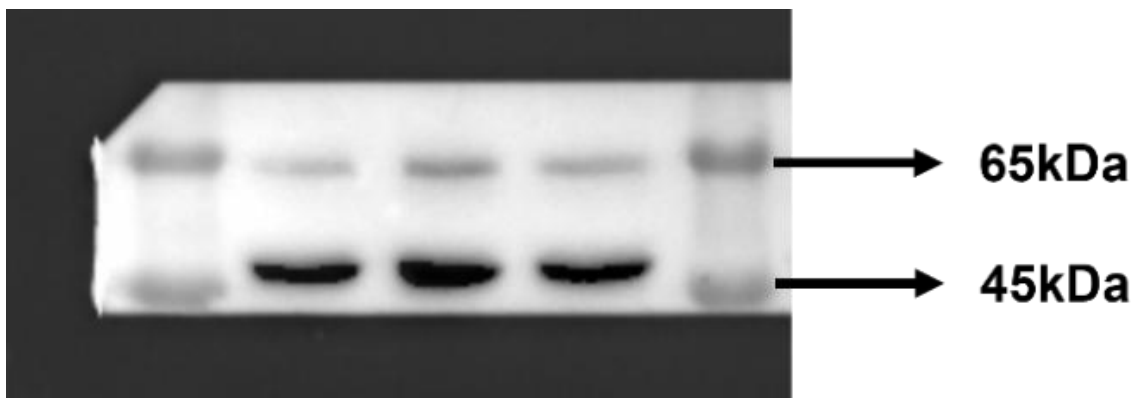

IRF7 (45-60kDa)

Lane information (From left to right): Sham, PTI, PTI+C-176; Each biological replicate set was separated by a molecular weight marker lane.

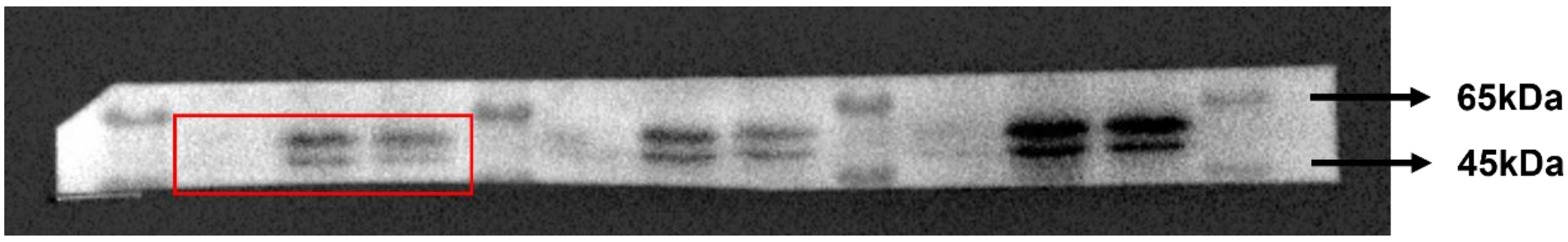

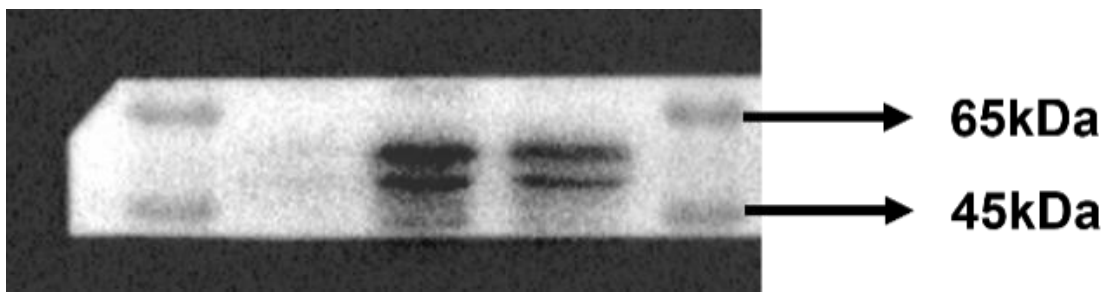

GAPDH (37kDa)

Lane information (From left to right): Sham, PTI, PTI+C-176; Each biological replicate set was separated by a molecular weight marker lane.

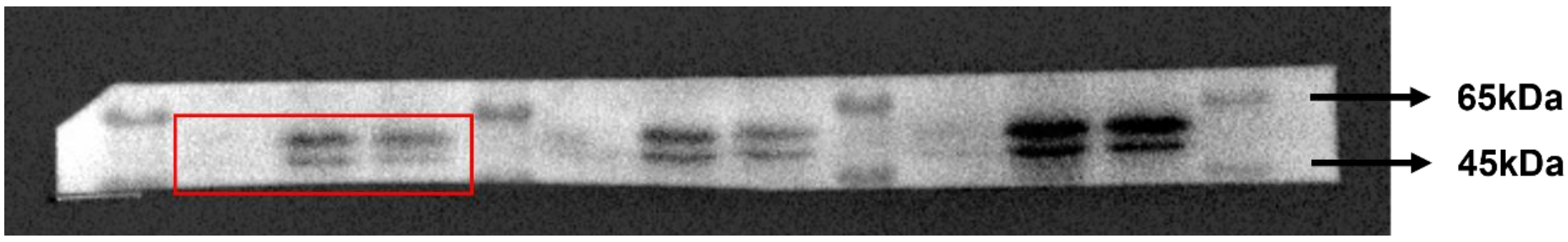

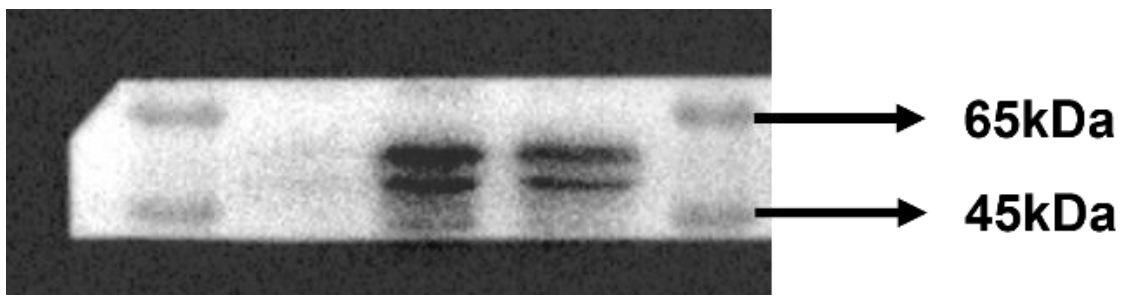

Supplement: Supplementary file 1 [file 12974_2026_3902_MOESM3_ESM.pdf]
