# Supplementary material for: Microglial IRF7-induced lipophagy impairment aggravates lipid droplet overload and impedes neurological recovery after ischemic stroke
Source: J Neuroinflammation. 2026 Jun 17;23:287. doi: 10.1186/s12974-026-03902-3 (PMC13508275; doi:10.1186/s12974-026-03902-3)
Supplement: Supplementary file 2 [file 12974_2026_3902_MOESM1_ESM.docx]

**Fig. S1 Generation and genotyping of conditional knockout mice. A** Schematic diagram of the hybrid strategy of generating *Cx3cr1-Cre; Irf7^fl/fl^* mice and *Irf7^fl/fl^* littermates. **B** Genotypic identification of *Irf7^fl/fl^* mice. *Irf7^+/+^*: one band with 124 bp, *Irf7^fl/fl^* homozygous: one band with 203 bp, *Irf7^fl/+^* heterozygous: two bands with 203 bp and 124 bp. **C** Genotypic identification of *Cx3cr1-Cre* mice. WT: no band, *Cx3cr1-Cre*: one band with 380 bp. (**A**) was created with BioRender.com.

**Fig. S2** **Spatiotemporal distribution of microglia and macrophages in the peri-infarct region following ischemic stroke. A** Representative immunofluorescence images showing colocalization of TMEM119 and Iba-1. Scale bars: 10 μm. **B** Percentage of TMEM119^+^Iba-1^+^ cells among Iba-1^+^ cells. n=4 mice per group. ^***^*P*<0.05 vs. sham group in (**B**), one-way ANOVA with Tukey’s post hoc test. Data are presented as mean ± SEM.

**Fig. S3** **TrC reduces LDs and promotes motor function recovery following ischemic stroke.** **A** Schematic illustration of the experimental design for TrC administration in WT mice. **B** Representative immunofluorescence images showing colocalization of PLIN2 (LDs) with Iba-1 (microglia) in the peri-infarct region from WT mice at day 14 after PTI. Scale bars: 10 μm. **C**-**F** Quantification of PLIN2 total volume, the number of Iba-1^+^ cells, Iba-1^+^ total volume and average Iba-1^+^ volume. n=6 mice per group. **G** Gait test of the left forelimb (LF), including stride frequency, stride length, stance width, paw area and ataxia coefficient. n=6 mice per group. **H** Gait test of the left hindlimb (LH), including stride frequency, stride length, stance width, paw area and ataxia coefficient. n=6 mice per group. ^*^*P*<0.05, ^***^*P*<0.001 in (**C**)-(**F**), one-way ANOVA with Tukey’s post hoc test. ^*^*P*<0.05, ^**^*P*<0.01, ^***^*P*<0.001 vs. PTI group in (**G**) and (**H**), two-way ANOVA with Bonferroni’s post hoc test. Data are presented as mean ± SEM. (**A**) was created with BioRender.com.

**Fig. S4** **Time-course changes in autophagy levels following ischemic stroke. A** Representative western blot images of beclin 1, LC3Ⅱ/Ⅰ, p62 and GAPDH. **B**-**E** Quantification of beclin 1, LC3Ⅱ/Ⅰ ratio, LC3Ⅱ and p62 levels, normalized to the sham group. n=4 mice per group. ^*^*P*<0.05, ^**^*P*<0.01, ^***^*P*<0.001 vs. sham group in (**B**)-(**E**), one-way ANOVA with Tukey’s post hoc test. Data are presented as mean ± SEM.

**Fig. S5 QC of scRNA-seq data from sham and pdMCAO groups.** **A**-**F** Scatter plots showing the number of genes, total gene expression counts and the percentage of mitochondrial genes in each individual sample. Sham group is shown in (**A**) and (**B**) (n=2), and pdMCAO group is shown in (**C**)-(**F**) (n=4).

**Fig. S6 *Irf7^high^* microglia subcluster engages autophagy and lipid metabolism pathways following ischemic stroke. A** UMAP visualization of major cell types identified by scRNA-seq from sham and tMCAO/R groups. **B** Pie plots showing the percentage of each cluster. **C** Dot plot showing the expression of marker genes across cell types shown in (**A**). **D** UMAP visualization of microglia subclusters from sham and tMCAO/R groups. **E** Pie plots showing the percentage of each microglia subcluster. **F** Dot plot showing the expression of marker genes across microglia subclusters in (**D**). **G** UMAP visualization illustrating the distribution of *Irf7* expression in microglia. **H** Violin plots showing *Irf7* expression across individual microglia subclusters in sham and tMCAO/R groups. **I** Dot plot showing GO enrichment analysis comparing *Irf7^high^* vs. *Irf7^low^* microglia within the MG1 subcluster.

**Fig. S7 Microglial *Irf7* deficiency reduces gliosis and infarct volume following ischemic stroke. A**-**C** Quantification of the number, volume, and average volume per cell of Iba-1^+^ microglia. n=6 mice per group. **D**-**F** Quantification of the number, volume, and average volume per cell of GFAP^+^ astrocytes. n=6 mice per group. **G** Representative images and quantitative analysis of infarct volume. n=6 mice per group. ^*^*P*<0.05, ^**^*P*<0.01, ^***^*P*<0.001 in (**A**)-(**F**), two-way ANOVA with Tukey’s post hoc test. ^**^*P*<0.01 in (**G**), unpaired t test. Data are presented as mean ± SEM.

**Fig. S8 Microglial *Irf7* deficiency exerts beneficial effects following ischemic stroke in 15-month-old mice. A** Schematic illustration of the experimental design for 15-month-old *Irf7^fl/fl^* and *Irf7* cKO mice. **B** Representative images of cerebral blood flow after dMCAO. **C** Representative immunofluorescence images showing colocalization of p62 and PLIN2 in microglia within the peri-infarct region from 15-month-old *Irf7^fl/fl^* and *Irf7* cKO mice at day 14 after dMCAO, along with magnified 3D-reconstructed images. Scale bars: 10 μm. **D** Quantification of PLIN2 volume in microglia. n=6 mice per group. **E** Quantification of the colocalization volume of PLIN2 and p62 in microglia. n=6 mice per group. **F** Representative images and quantitative analysis of infarct volume. n=6 mice per group. **G** Quantification of neurological deficit scores. n=6 mice per group. **H** Gait test of the right forelimb (RF), including stride frequency, stride length, stance width, paw area and ataxia coefficient. n=6 mice per group. **I** Gait test of the right hindlimb (RH), including stride frequency, stride length, stance width, paw area and ataxia coefficient. n=6 mice per group. ^*^*P*<0.05, ^***^*P*<0.001 in (**D**) and (**E**), two-way ANOVA with Tukey’s post hoc test. ^*^*P*<0.05 in (**F**), unpaired t test. ^*^*P*<0.05, ^**^*P*<0.01, ^***^*P*<0.001 vs. 15-month-old *Irf7^fl/fl^* PTI group in (**G**)-(**I**), two-way ANOVA with Bonferroni’s post hoc test. Data are presented as mean ± SEM. (**A**) was created with BioRender.com.

**Fig. S9 Time- and dose-dependent changes in LDs, autophagy and IRF7 in LPS-stimulated BV2 cells. A** Representative BODIPY (LDs) staining images of BV2 cells. Scale bars: 20 μm. **B**-**D** Quantification of LD number, size and the percentage of BODIPY⁺ cells. n=3 biologically independent cultures per group. **E** Quantification of *Irf7* mRNA expression. n=3 biologically independent cultures per group. **F** Representative western blot images of IRF7, Beclin 1, LC3Ⅱ/Ⅰ, p62 and GAPDH. **G**-**K** Quantification of IRF7, Beclin 1, LC3Ⅱ/Ⅰ ratio, LC3Ⅱ and p62 levels, normalized to the control group. n=3 biologically independent cultures per group. ^*^*P*<0.05, ^**^*P*<0.01, ^***^*P*<0.001 vs. control group, one-way ANOVA with Tukey’s post hoc test. Data are presented as mean ± SEM.

**Fig. S10 Validation of *Irf7* knockdown and *Gnai2* knockdown efficiency in BV2 cells. A** Quantification of *Irf7* mRNA expression. n=4 biologically independent cultures per group. **B** Representative western blot images of IRF7 and GAPDH. **C** Quantification of IRF7 levels, normalized to the NC group. n=3 biologically independent cultures per group. **D** Quantification of *Gnai2* mRNA expression. n=4 biologically independent cultures per group. **E** Representative western blot images of GNAI2 and GAPDH. **F** Quantification of GNAI2 levels, normalized to the NC group. n=3 biologically independent cultures per group. ^*^*P*<0.05, ^**^*P*<0.01, ^***^*P*<0.001 vs. NC group, one-way ANOVA with Tukey’s post hoc test. Data are presented as mean ± SEM.

**Fig. S11 Rapamycin enhances autophagic flux to reduce LD accumulation in LPS-stimulated BV2 cells. A** Representative images of BV2 cells transfected with mCherry-EGFP-LC3. Autophagosomes appear yellow, autolysosomes appear red. Rapa: rapamycin. Scale bar: 20 μm. **B**-**D** Quantification of mCherry^+^EGFP^+^ puncta, mCherry^+^EGFP^-^ puncta and autophagic flux rate. n=30 cells from 3 biologically independent experiments per group. **E** Representative BODIPY (LDs) staining images of BV2 cells. Scale bar: 20 μm. **F**-**H** Quantification of LD number, size and the percentage of BODIPY⁺ cells. n=6 biologically independent cultures per group. ^*^*P*<0.05, ^**^*P*<0.01, ^***^*P*<0.001 vs. control group, one-way ANOVA with Tukey’s post hoc test. Data are presented as mean ± SEM.

**Fig. S12 Correlation between *Irf7* and *Gnai2*.** Scatter plot showing the positive correlation between *Irf7* and *Gnai2* expression within the MG1 subcluster.

**Fig. S13 Exogenous CDP-choline supplementation alleviates LD accumulation and promotes motor function recovery following ischemic stroke.** **A** The PC levels of BV2 cells following *Irf7* knockdown. n=4 biologically independent cultures per group. **B** Quantification of *Gnai2* mRNA expression at different time points after PTI. n=4 mice per group. **C** The PC levels of *Irf7^fl/fl^* and *Irf7* cKO mice at day 14 after PTI. n=4 mice per group. **D** Schematic illustration of the experimental design for CDP-choline administration in WT mice. **E** The PC levels in WT mice at day 14 after PTI. n=4 mice per group. **F** Representative immunofluorescence images showing colocalization of p62 and PLIN2 in microglia within the peri-infarct region from WT mice at day 14 after PTI, along with magnified 3D-reconstructed images. Scale bars: 10 μm. **G** Quantification of PLIN2 volume in microglia. n=6 mice per group. **H** Quantification of the colocalization volume of PLIN2 and p62 in microglia. n=6 mice per group. **I** Gait test of the left forelimb (LF), including stride frequency, stride length, stance width, paw area and ataxia coefficient. n=6 mice per group. **J** Gait test of the left hindlimb (LH), including stride frequency, stride length, stance width, paw area and ataxia coefficient. n=6 mice per group. ^*^*P*<0.05, ^**^*P*<0.01, ^***^*P*<0.001 in (**B**), (**E**), (**G**) and (**H**), one-way ANOVA with Tukey’s post hoc test. ^**^*P*<0.01, ^***^*P*<0.001 in (**A**) and (**C**), two-way ANOVA with Tukey’s post hoc test. ^*^*P*<0.05, ^**^*P*<0.01, ^***^*P*<0.001 vs. PTI group in (**I**) and (**J**), two-way ANOVA with Bonferroni’s post hoc test. Data are presented as mean ± SEM. (**D**) was created with BioRender.com.

**Fig. S14 Spatiotemporal changes of STING signaling expression at different time points after PTI. A** Quantification of *Sting* mRNA expression. n=4 mice per group. **B** Representative western blot images of STING and GAPDH. **C** Quantification of STING levels, normalized to the sham group. n=4 mice per group. **D** Representative immunofluorescence images showing colocalization of STING with Iba-1 (microglia) and GFAP (astrocytes) in the peri-infarct region. Scale bars: 50 μm. ^*^*P*<0.05, ^**^*P*<0.01, ^***^*P*<0.001 vs. sham group, one-way ANOVA with Tukey’s post hoc test. Data are presented as mean ± SEM.

**Fig. S15 Effects of C-176 on infarct volume, downstream STING signaling proteins and gliosis. A** Representative images and quantitative analysis of infarct volume. n=6 mice per group. **B** Representative western blot images of p-TBK1, TBK1, p-IRF7, IRF7 and GAPDH. **C**-**H** Quantification of p-TBK1, TBK1, p-IRF7, IRF7 levels, normalized to the Sham group. n=4 mice per group. **I**-**K** Quantification of the number, volume, and average volume per cell of Iba-1^+^ microglia. n=6 mice per group. **L**-**N** Quantification of the number, volume, and average volume per cell of GFAP^+^ astrocytes. n=6 mice per group. ^*^*P*<0.05, ^**^*P*<0.01, ^***^*P*<0.001 vs. sham group, one-way ANOVA with Tukey’s post hoc test. Data are presented as mean ± SEM.

**Fig. S16 The STING inhibitor C-176 reduces LD accumulation in LPS-stimulated primary microglia and BV2 cells. A** Representative BODIPY (LDs) staining images of primary microglia. Scale bar: 20 μm. **B**-**D** Quantification of LD number, size and the percentage of BODIPY⁺ cells. n=6 biologically independent cultures per group. **E** Representative BODIPY (LDs) staining images of BV2 cells. Scale bar: 20 μm. **F**-**H** Quantification of LD number, size and the percentage of BODIPY⁺ cells. n=6 biologically independent cultures per group. ^*^*P*<0.05, ^**^*P*<0.01, ^***^*P*<0.001, one-way ANOVA with Tukey’s post hoc test. Data are presented as mean ± SEM.
