# Supplementary material for: Microglial IRF7-induced lipophagy impairment aggravates lipid droplet overload and impedes neurological recovery after ischemic stroke
Source: J Neuroinflammation. 2026 Jun 17;23:287. doi: 10.1186/s12974-026-03902-3 (PMC13508275; doi:10.1186/s12974-026-03902-3)
Supplement: Supplementary file 3 [file 12974_2026_3902_MOESM2_ESM.docx]

**Table S1. A list of primers sequences for mouse genotyping.**

| Genes | Forward (5’-3’) | Reverse (5’-3’) |
| --- | --- | --- |
| *Irf7^fl/fl^* | ACTGGACACAAACACTCTAAGACA | CTGTCAGGAGACAGGACATAGTG |
| *Cx3cr1-Cre* | GACATTTGCCTTGCTGGAC | GCAGGGAAATCTGATGCAAG |

**Table S2. A list of primers sequences for RT–qPCR.**

| Genes | Forward (5’-3’) | Reverse (5’-3’) |
| --- | --- | --- |
| *Gapdh* | GTCGGTGTGAACGGATTTGG | GCTCCTGGAAGATGGTGATGG |
| *Irf7* | AGTGCTGTTTGGAGACTGGC | ACAGCCCAGGCCTTGAAGA |
| *Gnai2* | TGGCTGAGGATGAGGAGATGAAC | TTGAGGAAGAGGATGATGGAGGTG |
| *Sting* | CGTGCTGGCATCAAGAATCG | ACTCCAGGATACAGACGCCT |

**Table S3. A list of primary and secondary antibodies for western blot.**

| Antibody | Host | Dilution | Product Number, Company, Country |
| --- | --- | --- | --- |
| GAPDH | rabbit | 1:6000 | Cat# AP0063; Bioworld, China |
| beclin 1 | rabbit | 1:1200 | Cat# 3495; Cell Signaling Technology, USA |
| LC3B | rabbit | 1:1200 | Cat# T55992; Abmart, China |
| SQSTM1/p62 | rabbit | 1:1200 | Cat# 23214; Cell Signaling Technology, USA |
| STING | rabbit | 1:1200 | Cat# ab288157; Abcam, USA |
| IRF7 | rabbit | 1:1200 | Cat# 72073; Cell Signaling Technology, USA |
| GNAI2 | rabbit | 1:1200 | Cat# TN26813; Abmart, China |
| Phospho-IRF7 (Ser437/438) | rabbit | 1:1000 | Cat# 24129; Cell Signaling Technology, USA |
| TBK1 | rabbit | 1:1000 | Cat# 3504; Cell Signaling Technology, USA |
| Phospho-TBK1 (Ser172) | rabbit | 1:1000 | Cat# 5483; Cell Signaling Technology, USA |
| HRP-conjugated AffiniPure goat anti-rabbit IgG |  | 1:6000 | Cat# BA1054; Boster, China |

**Table S4. A list of primary and secondary antibodies for immunofluorescence.**

| Antibody | Host | Dilution | Product Number, Company, Country |
| --- | --- | --- | --- |
| PLIN2 | guinea pig | 1:100 | Cat# GP42; PROGEN, Germany |
| Iba-1 | goat | 1:200 (tissue)  1:50 (cell) | Cat# NB100-1028; Novus Biologicals, USA |
| GFAP | rabbit | 1:1800 | Cat# ab7260; Abcam, USA |
| SQSTM1/p62 | rabbit | 1:1600 | Cat# 23214; Cell Signaling Technology, USA |
| STING | rabbit | 1:500 | Cat# ab288157; Abcam, USA |
| TMEM119 | rabbit | 1:100 | Cat# ab209064; Abcam, USA |
| GNAI2 | rabbit | 1:100 | Cat# TN26813; Abmart, China |
| YSFluor™488 Donkey Anti-Guinea Pig IgG (H+L) |  | 1:400 | Cat# 34506ES60; Yeasen, China |
| YSFluor™ 594 Donkey Anti-Goat IgG (H+L) |  | 1:400 | Cat# 34312ES60; Yeasen, China |
| YSFluor™ 647 Donkey Anti-Rabbit IgG (H+L) |  | 1:400 | Cat# 34213ES60; Yeasen, China |

**Table S5. A list of siRNA target sequences for gene knockdown.**

| Genes | Target sequence (5’-3’) |
| --- | --- |
| *Irf7*-siRNA-1 | GCATAAGGTGTACGAACTT |
| *Irf7*-siRNA-2 | CCGAGAACTGGAGGAGTTT |
| *Irf7*-siRNA-3 | TCTGGATGAAGAAGATGCA |
| *Gnai2*-siRNA-1 | GGATCTAAATAAGCGCAAA |
| *Gnai2*-siRNA-2 | CCAGCTACATCCAGAGCAA |
| *Gnai2*-siRNA-3 | CCAAGAACGTGCAGTTTGT |

**Table S6. A list of primers sequences for ChIP–qPCR.**

| Genes | Forward (5’-3’) | Reverse (5’-3’) |
| --- | --- | --- |
| *Gnai2* | GTTAGCTCTACCCTTGGTGTCTTC | TTAGGGATGGTGATTGGTTGGAG |
